# Photoreforming of solid waste on 1 m^2^ scale using single-source precursor-derived co-catalyst films

**DOI:** 10.1038/s44286-026-00406-y

**Published:** 2026-06-24

**Authors:** Ariffin Bin Mohamad Annuar, Yongpeng Liu, Subhajit Bhattacharjee, Jonathan Slaughter, Iuliia Mikulska, Farheen N. Sayed, Clare P. Grey, Dominic S. Wright, Erwin Reisner

**Affiliations:** 1 Yusuf Hamied Department of Chemistry, University of Cambridge, Cambridge, UK; 2 Faraday Institution, Quad 1, Harwell Science and Innovation Campus, Didcot, UK; 3 Diamond Light Source, Harwell Science and Innovation Campus, Didcot, UK

**Keywords:** Photocatalysis, Chemical engineering

## Abstract

Photocatalytic reforming offers a route to valorize biomass and plastic waste into clean H_2_ under ambient conditions. However, the scalability of photocatalyst sheets is limited by high-temperature processing, binders and complex co-catalyst deposition. Here a [Co_4_Zr_2_O(O^*n*^Pr)_10_(acac)_4_] single-source precursor is deposited onto Al-doped SrTiO_3_ and immobilized on glass substrates to fabricate photocatalyst sheets using multiple techniques, including high-throughput spray coating. The resulting sheets enable photoreforming of cellulose- and polyethylene terephthalate-derived feedstocks, producing H_2_ alongside value-added organics such as formate, acetate, glycolate and glycolaldehyde dimer. The system is demonstrated from the centimeter- to meter-squared scale, culminating in a 1-m^2^ outdoor reactor operating under natural sunlight. After 6 h, H_2_ yields reached 5.24 and 1.51 mmol m^−2^ for glucose and pretreated cellulose, respectively, with concurrent formation of oxygenates. Techno-economic analysis based on real-world data estimates an H_2_ cost of £0.93 mmol^−1^. This work advances scalable photocatalytic reforming and provides a step towards practical deployment.

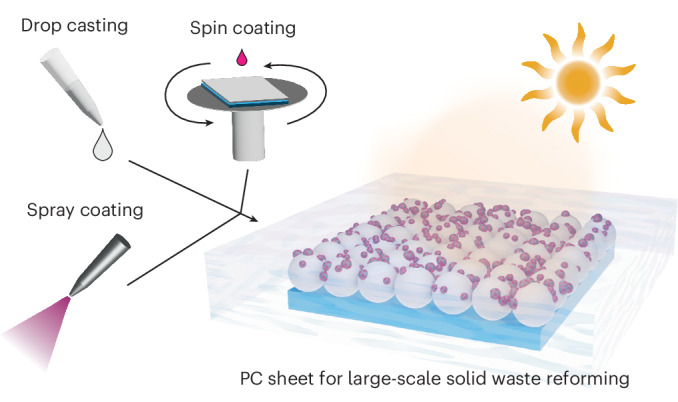

## Abstract (web summary)

This study demonstrates scalable photocatalytic reforming by leveraging single-source precursor chemistry and versatile deposition techniques, including drop casting, spin coating and high-throughput spray coating, to fabricate photocatalyst sheets from the centimeter- to meter-squared scale. Outdoor deployment of large-scale solid waste reforming advances practical solar-driven systems for sustainable hydrogen production and waste valorization.

## Main

Hydrogen (H_2_) is a key clean energy vector and industrial feedstock, used in fuel cells and for the production of chemicals such as ammonia, methanol and steel, among others^1–3^. Its global demand has nearly doubled over the past two decades, yet over 95% of current H_2_ production remains fossil-derived or obtained as a byproduct of petrochemical processes^4^. The electrolysis of water, alongside emerging photoelectrochemical (PEC) and photocatalytic water splitting technologies, offers promising routes toward low-emission H_2_ production. However, these approaches are constrained by the large thermodynamic barrier of overall water splitting (Gibbs free energy change, Δ*G*° = +237.2 kJ mol^−1^) and the sluggish kinetics of the four-electron water oxidation reaction^5,6^. Despite advances in water oxidation co-catalysts, including IrO_2_, RuO_2_ and Co-based systems, challenges such as high overpotentials, limited stability and high cost persist^7–9^. In parallel, the generation and inadequate management of waste, particularly biomass and plastics, presents a major environmental challenge. These materials are often disposed of via landfilling, incineration or energy-intensive processes such as pyrolysis and gasification, which are typically nonselective and carbon intensive^10,11^. Converting waste streams into valuable products using clean energy sources is therefore an attractive strategy to address both energy and environmental concerns.

Solar reforming has emerged as a class of sunlight-driven technologies capable of converting waste-derived substrates into fuels and chemicals. By utilizing substrates such as glucose or ethylene glycol (EG), derived from lignocellulosic biomass or polyethylene terephthalate (PET), as electron donors, solar reforming enables a more energetically favorable oxidation pathway compared with water oxidation^12,13^. Photocatalytic reforming, based on semiconductor photocatalysts (PCs), offers a simple, one-pot approach for solar fuel production^14^. In some cases, photothermal systems producing liquid organic hydrogen carriers, such as formic acid, have been integrated with photocatalysis to enhance overall hydrogen yields from complex substrates^15^. A major limitation to the practical deployment of photocatalytic reforming lies in the fabrication of simple, scalable and reusable PC systems. In this context, PC sheets (developed by immobilizing PC composites onto suitable support panels) offer an effective way to scale up PC processes, promote catalyst reuse, decrease product contamination and minimize shielding or scattering of incident light^16^. While a limited number of studies have demonstrated m^2^-scale hydrogen generation using PC sheets, including a 100-m^2^ prototype, these systems typically utilize distilled water or model substrates rather than real waste streams^17^. Nevertheless, they highlight the potential of PC sheets for scalable solar-to-chemical conversion.

Despite this promise, the fabrication of PC sheets remains challenging. Conventional approaches often require high-temperature annealing to ensure mechanical stability and prevent catalyst detachment^18^. In addition, co-catalysts are typically required to enhance charge transport and suppress recombination^19^, but their deposition commonly involves wet chemical methods or photodeposition, which may require ultraviolet (UV) irradiation, high temperatures or excess precursor use^20,21^. These factors limit scalability and increase cost and complexity.

Single-source precursor (SSP) chemistry offers a promising alternative for scalable catalyst deposition. SSPs can be synthesized via simple synthetic routes and deposited onto substrates to form catalytically active mixed-metal films with controlled composition and loading^22,23^. Their compatibility with a range of deposition techniques, from simple drop casting to high-throughput spray coating, makes them particularly attractive for large-area fabrication^24^. SSP-derived films have been applied in electrochemical and PEC systems, including water splitting and alcohol oxidation^23,25,26^. However, their application in purely photocatalytic systems, particularly for waste reforming, remains largely unexplored. Existing studies are typically limited to laboratory-scale systems and often still rely on annealing or binders^23,26^, highlighting the need for high-throughput SSP deposition methods for large-area PC sheets.

Here, we introduce SSP chemistry as a scalable strategy for fabricating PC sheets and demonstrate its application in solid-waste reforming at the meter-squared scale. The PC sheets consist of Al-doped SrTiO_3_ (Al:SrTiO_3_) deposited on glass substrates as the light-absorbing layer, over which a thin Co–Zr mixed-metal co-catalyst layer is formed via deposition of a [Co_4_Zr_2_O(O^*n*^Pr)_10_(acac)_4_] SSP. This layer enhances charge extraction and improves photocatalytic performance. The composite sheets were fabricated across multiple length scales, from centimeter-squared to meter-squared, using drop casting, spin coating and spray coating, demonstrating their versatility and scalability. The performance of the PC sheets was evaluated for H_2_ evolution and the reforming of pretreated polymeric waste streams, including cellulose and real-world PET bottles, yielding value-added products alongside H_2_. A 1-m^2^ photocatalytic reforming system is demonstrated under natural sunlight using a custom-built reactor. Finally, a techno-economic analysis (TEA) is conducted based on experimental data to assess the feasibility of the process. Overall, this work demonstrates the potential of SSPs in the large-scale fabrication of robust PC sheets and serves as a foundation for developing photocatalytic reforming systems for H_2_ generation coupled to waste valorization under outdoor conditions on a meter-squared scale.

## Results

### Synthesis and characterization of the SSP

The Co–Zr SSP species, [Co_4_Zr_2_O(O^*n*^Pr)_10_(acac)_4_] (O^*n*^Pr, *n*-propoxide; acac, acetylacetonate), was synthesized by reacting cobalt(II) acetylacetonate with zirconium *n*-propoxide in *n*-propanol and heating under reflux in toluene for 1 h under dry and O_2_-free N_2_. The solvents were removed under vacuum, and the purple residue was redissolved in *n*-hexane and stored at –30 °C for 16 h to produce large purple cubic crystals of a Co–Zr SSP. This method is an improvement of a previously reported synthesis, involving much shorter reaction times and high yields^27^.

**Fig. 1:**
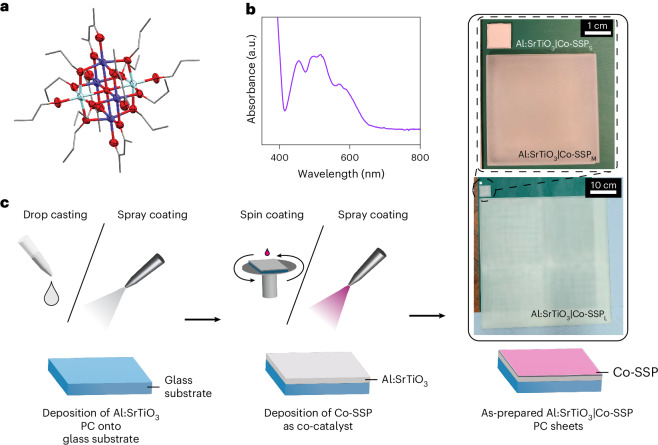
Characterization of SSP and fabrication procedure for the Al:SrTiO_3_|Co-SSP system. **a**, Molecular structure of Co_4_Zr_2_O(O^*n*^Pr)_10_(acac)_4_. H-atoms and minor disorder of some *n*-propoxide ligands omitted. Blue, cyan and red ellipsoids correspond to Co, Zr and O, respectively. Organic ligands are represented as gray sticks. **b**, Solution UV−vis spectrum of Co-SSP solution. The UV−vis spectrum was recorded in THF (0.04 mM) in a quartz cuvette with path length 1 cm. **c**, Fabrication procedure of Al:SrTiO_3_|Co-SSP with different dimensions and photographs showing the size comparison between Al:SrTiO_3_|Co-SSP_S_, Al:SrTiO_3_|Co-SSP_M_ and Al:SrTiO_3_|Co-SSP_L_. S, M and L indicate small (1 cm^2^), medium (20.25 cm^2^) and large (0.25 m^2^) PC sheet, respectively.

The Co–Zr SSP crystals were analyzed by single-crystal X-ray diffraction (XRD). The crystal structure of the SSP is shown in Fig. 1a (a more detailed figure including selected bond lengths and angles is shown in Supplementary Fig. 1; details on the crystallographic data are presented in Supplementary Table 1). This structure is a redetermination of a previously reported structure, but the earlier measurement was performed at room temperature and did not include hydrogen atoms^27^. The SSP cage has a hexanuclear structure, with four cobalt atoms in one plane and two zirconium atoms above and below this plane. The six metals form an octahedral arrangement, with the metals bridged by O^*n*^Pr or acac ligands. In addition, there is a μ_6_-oxo-ligand in the middle of the cage bonding to all the metals. It is assumed that this is a result of the partial hydrolysis of the starting material Zr(O^*n*^Pr)_4_ in ^*n*^PrOH (ref. ^28^). Thermogravimetric analysis (TGA) indicates that the thermal decomposition of the Co–Zr SSP crystals occurs in one combined step, with the main weight loss occurring at ~200−400 °C being attributed to the loss of organic ligands (Supplementary Fig. 2). The Co–Zr SSP crystals were solubilized in tetrahydrofuran (THF) to form the Co-SSP deposition solution. The solution UV−visible (vis) spectrum of the Co-SSP solution shows the *d*–*d* transitions corresponding to the transitions of Co^2+^ ions (Fig. 1b).

### Assembly and characterization of Al:SrTiO_3_|Co-SSP PC sheets

The PC sheets consist of Al:SrTiO_3_ nanoparticles as the light absorber, which were synthesized using a previously reported flux method (see the Methods for details)^29^. The Al:SrTiO_3_ powders are inexpensive and stable, have good photo-activity and are easy to synthesize and immobilize, serving as an ideal candidate for scalable applications. For the fabrication of the PC sheets, the synthesized Al:SrTiO_3_ powders were dispersed in isopropanol and deposited using either drop casting or spray coating onto glass supports (Fig. 1c and Supplementary Table 2). While drop casting is more suitable for small-scale (1 cm^2^) screening and optimization experiments, spray coating allows facile and practical deposition on larger supports (for example, 1 m^2^, as discussed later). Thus, both deposition methods were explored in this work.

**Fig. 2:**
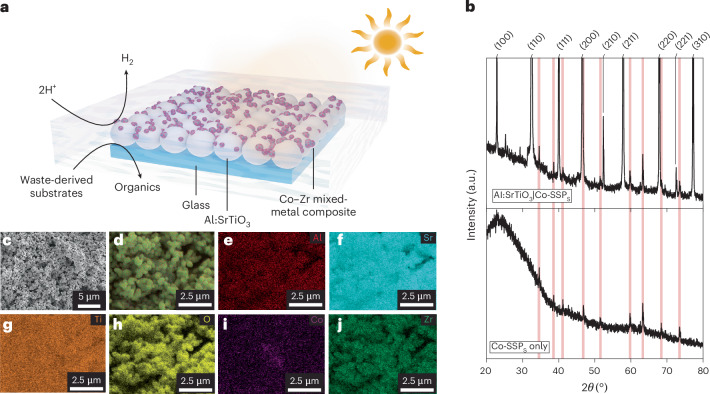
Working principle of the PC sheet and characterization of Al:SrTiO_3_|Co-SSP_S_. **a**, Illustration of PC sheet architecture. **b**, Powder XRD patterns for Al:SrTiO_3_|Co-SSP_S_ and only Co-SSP deposited directly onto glass. The sharp intense peaks due to SrTiO_3_ are labeled with their Miller indices. The red bands indicate reflections of Co-SSP that are attributed to the presence of Co-SSP in Al:SrTiO_3_|Co-SSP_S_. **c**, Top-view SEM image of Al:SrTiO_3_|Co-SSP_S_. **d**–**j**, Overall (**d**), aluminum (**e**), strontium (**f**), titanium (**g**), oxygen (**h**), cobalt (**i**) and zirconium (**j**) EDX elemental mapping.

The H_2_ evolution co-catalyst layer was next deposited over the Al:SrTiO_3_ light absorber. In this regard, SSP chemistry enables the facile and uniform deposition of active mixed-metal composite catalytic films at room temperature. The Co-SSP was chosen to form the co-catalyst film as Co has been well reported as an abundant, noble metal-free hydrogen evolution co-catalyst, while Zr acts as a robust, inert matrix upon which the Co species are decorated^30^. Upon deposition of the SSP solution on a support using standard techniques (discussed later) under ambient conditions, a Co–Zr mixed-metal composite layer is formed that is catalytically active. The one-step synthetic process of SSPs and the simple deposition routes to obtain catalyst films make it appealing for practical and scalable applications, which is demonstrated through this work. The as-prepared SSP solution was spin coated (on drop-casted Al:SrTiO_3_ sheets) or spray coated (on spray-coated Al:SrTiO_3_ sheets) to form a thin, semi-transparent layer of Co–Zr mixed-metal composite (Figs. 1c and 2a and Supplementary Table 2). This mixed-metal composite layer (referred to as the Co-SSP layer) acts as the H_2_ evolution co-catalyst, while the Al:SrTiO_3_ is capable of organic substrate oxidation^31^, thus forming an integrated photocatalytic reforming catalyst (Fig. 2a). The Al:SrTiO_3_|Co-SSP PC sheets prepared by drop casting Al:SrTiO_3_ and spin coating Co-SSP (referred to as Al:SrTiO_3_|Co-SSP_S_) were used for the 1-cm^2^ small-scale experiments involving optimization of reaction parameters and substrate screening as drop casting and spin coating allow better control of catalyst deposition at small scales with more precise loading variation.

The powder XRD pattern of Al:SrTiO_3_|Co-SSP_S_ with optimized catalyst loading revealed the characteristic reflections of SrTiO_3_ (ICDD 00-035-0734) as seen in previous reports^32^, along with weak reflections indexed to CoO_*x*_ and ZrO_2_ peaks (Fig. 2b and Supplementary Fig. 3), indicating the presence of the co-catalyst layer. The morphology of the optimized PC sheets was determined using scanning electron microscopy (SEM) as shown in Fig. 2c, where the corresponding energy-dispersive X-ray (EDX) elemental maps further attested to the successful deposition of Co-SSP over the Al:SrTiO_3_ PC layer (Fig. 2d–j and Supplementary Fig. 4). Transmission electron microscopy (TEM) images, bright-field scanning TEM and corresponding EDX elemental maps also show the dispersion of the Co-SSP on the Al:SrTiO_3_ particles (Supplementary Figs. 5 and 6). X-ray photoelectron spectroscopy (XPS) of the as-prepared PC sheets further confirmed the presence of each of the expected elements (Supplementary Fig. 7). There was also no change in the structure of the deposited Co-SSP with time, as indicated by UV−vis diffuse reflectance spectroscopy and Fourier-transform infrared (FTIR) spectroscopy of freshly deposited Co-SSP and the Co-SSP after 24-h storage under ambient conditions (Supplementary Figs. 8 and 9).

### Photocatalytic reforming of model and polymeric substrates

The Al:SrTiO_3_|Co-SSP_S_ sheets (involving drop-casted Al:SrTiO_3_ and spin-coated Co-SSP) with an area of 1 cm^2^ were first used for the photocatalytic reforming experiments using different substrates. The small-scale photocatalytic experiments were conducted in air-tight top-irradiation-type photoreactors (solution volume 10 ml, headspace 40 ml) under simulated solar irradiation (AM1.5G, 100 mW cm^‒2^) at room temperature (Supplementary Fig. 10).

**Fig. 3:**
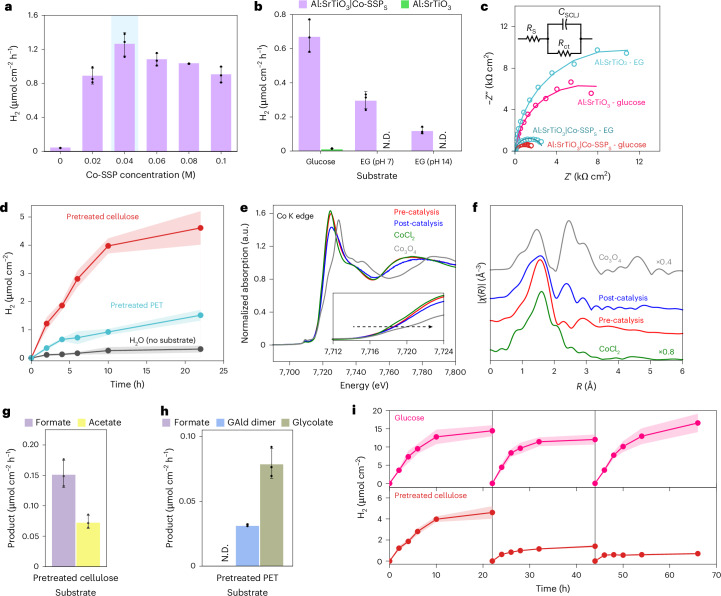
Performance of Al:SrTiO_3_|Co-SSP_S_ for photocatalytic reforming. **a**, Loading optimization of Co-SSP on the Al:SrTiO_3_ PC sheets. **b**, H_2_ evolution using Al:SrTiO_3_|Co-SSP_S_ and Al:SrTiO_3_ only with glucose and EG as model substrates. Experiments at pH 7 and 14 were conducted in ultrapure water and 1.0 M KOH solution, respectively. **c**, Nyquist plots of the PEIS response (open circles) recorded with frequency ranging from 1 MHz to 0.5 Hz and a 15-mV sinusoidal A.C. perturbation amplitude at –0.4 V versus reversible hydrogen electrode (RHE) as well as the corresponding fitting curves (solid lines). Inset: proposed Randles equivalent circuit, *C*_*S*CLJ_ was replaced by a constant phase element (CPE) during fitting to account for the non-ideal capacitive behaviors of the photoelectrodes. Measurements were performed in 30 ml stirred electrolyte containing 0.1 M Na_2_SO_4_ and 0.1 M substrate (glucose or EG) under AM1.5G illumination. **d**, Time-course H_2_ evolution of Al:SrTiO_3_|Co-SSP_S_ with waste-derived substrates and without substrates. **e**,**f**, Co K-edge XANES spectra (**e**) and corresponding Fourier-transformed EXAFS spectra (**f**). The Fourier-transformed spectra were shifted vertically for clarity. **g**,**h**, Oxidation products of Al:SrTiO_3_|Co-SSP_S_ using pretreated cellulose (**g**) and pretreated PET (**h**) as substrate. **i**, PC reusability tests. Vertical lines indicate redeposition of the Co-SSP co-catalyst on the same Al:SrTiO_3_ PC sheets after 22 h and 44 h. Conditions (**a**, **b**, **d**, **g–i**): The photocatalytic experiments were performed under AM1.5G illumination at room temperature in an N_2_ atmosphere. For the PC reusability test, photocatalytic experiments were performed under AM1.5G illumination for 22 h at room temperature each cycle. N.D., not detected. The data in **a**, **b**, **d** and **g**–**i** are presented as mean values ± s.d. for reactions performed in triplicate (*n* = 3; individual data points represented as dots).

Before testing different substrates for photocatalytic reforming, the optimum loading of the Co-SSP co-catalyst layer was determined by spin coating 30 µl of Co-SSP solution (in THF) at different concentrations over the Al:SrTiO_3_ layer (having an optimized loading of 2 mg cm^‒2^; Supplementary Fig. 11 and Supplementary Table 3)^33^, and carrying out photocatalytic reactions using triethanolamine (TEOA) as the electron donor in aqueous medium. TEOA was selected for initial screening as it has been widely employed as a sacrificial substrate for photocatalytic studies^18^. The amount of H_2_ evolved after the photocatalytic reforming experiments was determined using gas chromatography (GC) with 2% CH_4_ as an internal standard (see calibration curve in Supplementary Fig. 12). Inductively coupled plasma optical emission spectroscopy (ICP-OES) showed Co and Zr loadings in the range of 0–1.97% and 0–1.76%, respectively, after spin coating different concentrations of Co-SSP solution (Supplementary Table 4). A concentration of 0.04 M Co-SSP showed the highest H_2_ production rate of 1.26 ± 0.14 μmol cm^−2^ h^−1^ and was therefore used as the optimized concentration for subsequent experiments (Fig. 3a and Supplementary Table 5). Increasing and decreasing the Co-SSP concentration above or below this optimized concentration was detrimental to performance, probably because lower Co-SSP loadings result in a smaller number of active sites for proton reduction. Conversely, higher loadings possibly hinder light absorption by Al:SrTiO_3_, leading to lower H_2_ production. This light-shielding effect was also the reason for avoiding drop casting the Co-SSP co-catalyst onto the PC sheets as it results in thicker, light-blocking films (as measured from SEM images in Supplementary Fig. 13), leading to a drastic drop in photocatalytic performance (Supplementary Fig. 14 and Supplementary Table 6). This was further confirmed in internal quantum efficiency (IQE) measurements where the PC sheets with drop-casted Co-SSP had a lower IQE at 350 nm than those with spin-coated Co-SSP (2.29% and 3.75% for the former and latter, respectively; Supplementary Fig. 15 and Supplementary Table 7). Control experiments also show that the PC sheets with Co-SSP alone had no activity, necessitating both the Al:SrTiO_3_ and Co-SSP as light absorber and co-catalyst, respectively, for the PC system to function (Supplementary Fig. 16 and Supplementary Table 8).

After optimizing the Al:SrTiO_3_|Co-SSP_S_ PC sheets and before the use of polymeric biomass and plastic substrates, the sheets were employed for the photocatalytic reforming of model substrates to realize the maximum efficiency of the process in the absence of depolymerized byproducts associated with pretreatment steps (for details on substrate selection, see Supplementary Discussion 1). Figure 3b shows that the H_2_ production rates obtained were 0.67 ± 0.09, 0.30 ± 0.05 and 0.12 ± 0.02 µmol cm^‒2^ h^‒1^ for glucose, EG (pH ~7) and EG (pH ~14), respectively, whereas only trace amounts of H_2_ were produced from pristine Al:SrTiO_3_ (without any Co-SSP layer; Supplementary Table 9). Particularly for glucose, the amount of H_2_ produced using the Co–Zr transition element-based SSP-derived co-catalyst was comparable to that obtained using a benchmark RhCrO_*x*_ co-catalyst (Supplementary Discussion 2).

To gain insights into the catalytic effects of Co-SSP, PEC impedance spectroscopy (PEIS) was performed on an Al:SrTiO_3_ PC sheet acting as the working electrode under various conditions to study the charge carrier dynamics of the photocatalytic system (the suitability of the Al:SrTiO_3_ photoelectrode is indicated by the photocurrent response in Supplementary Fig. 17). The Nyquist plot of the impedance response (Fig. 3c) was modeled using a Randles circuit^34^ (Fig. 3c, inset) consisting of a series resistor (${R}_{{\rm{S}}}$), a charge transfer resistor (${R}_{\mathrm{ct}}$) and a capacitor representing the semiconductor–liquid junction (${C}_{\mathrm{SCLJ}}$). In the presence of 0.1 M glucose, the addition of Co-SSP reduced ${R}_{\mathrm{ct}}$ from 13.4 to 1.6 kΩ. From the values of ${R}_{\mathrm{ct}}$ and ${C}_{\mathrm{SCLJ}}$, the pseudo first-order rate constants for charge transfer (${k}_{\mathrm{ct}}$) for the hydrogen evolution reaction (HER)^35^ were calculated to be 3.7 and 20.7 s^−1^ for Al:SrTiO_3_ and Al:SrTiO_3_|Co-SSP, respectively. Similarly, for EG oxidation coupled with HER, the incorporation of Co-SSP led to an 86% reduction in ${R}_{\mathrm{ct}}$ and a 370% increase in ${k}_{\mathrm{ct}}$ compared with pristine Al:SrTiO_3_, yielding values of 2.7 kΩ and 16.0 s^−1^, respectively (Supplementary Table 10). A similar trend in ${R}_{\mathrm{ct}}$ and ${k}_{\mathrm{ct}}$ values was observed with 0.1 M TEOA as the oxidation substrate (Supplementary Fig. 18 and Supplementary Table 10). These PEIS results demonstrate that Co-SSP serves as an effective co-catalyst for HER, enhancing the charge transfer process and drastically improving reaction kinetics. The differences in ${R}_{\mathrm{ct}}$ and ${k}_{\mathrm{ct}}$ values obtained from glucose and EG oxidation also support the better performance of Al:SrTiO_3_|Co-SSP_S_ when glucose versus EG was used as the substrate (Fig. 3b). The catalytic role of the Co-SSP co-catalyst is discussed further in Supplementary Discussion 3.

The polymeric waste substrates, cellulose and a real-world PET plastic bottle, were next utilized for the photocatalytic reforming experiments following a pretreatment step (hydrolysis using the enzyme cellulase and alkaline treatment for cellulose and PET, respectively; Supplementary Discussion 1). Figure 3d shows the evolution of H_2_ with time in the presence of pretreated polymeric substrates (Supplementary Table 11). After 22 h of reaction, 4.61 ± 0.59 and 1.51 ± 0.19 μmol cm^−2^ of H_2_ (corresponding to 0.21 ± 0.03 and 0.07 ± 0.01 μmol cm^−2^ h^−1^) were produced when using pretreated cellulose and PET, respectively, as the electron donor substrate. In the absence of any substrate (Fig. 3d and Supplementary Tables 11 and 12), the H_2_ produced in the same period was only 0.32 ± 0.12 μmol cm^−2^. It is noted, however, that the overall rate of product formation of Al:SrTiO_3_|Co-SSP_S_ using pretreated polymeric substrates was lower than that observed in the case of model substrates (Supplementary Table 9 and 13), consistent with previous reports^14^. Residual fragments or oligomers in the pretreated solution can block catalytically active sites in the absence of stirring or scatter incident light, thereby lowering the production rates.

X-ray absorption spectroscopy (XAS) at the Co K edge was carried out to track changes in the Co-SSP after photocatalysis (see the Methods for details). The X-ray absorption near-edge structure (XANES) spectra (Fig. 3e) show that the Co K edge slightly shifts to a higher energy after catalysis, indicating that Co becomes more oxidized during the reaction. These findings were further validated through linear combination fitting analysis. The XANES profile of the post-catalysis sample was accurately reproduced using reference spectra from the pre-catalysis sample and two reference compounds, Co_3_O_4_ and CoCl_2_ (Supplementary Fig. 19). These compounds were selected because they represent materials containing tetrahedral Co^2+^ and octahedral Co^3+^ ions (Co_3_O_4_), and octahedrally coordinated Co^2+^ (CoCl_2_). This indicates that the Co species before and after photocatalysis exhibit distinct electronic structures.

The corresponding Fourier-transformed extended X-ray absorption fine structure (EXAFS) spectra (Fig. 3f) further reveal changes in the local environment of Co, especially within the first and second coordination shells. Comparison with Co_3_O_4_, CoCl_2_ and reference samples shows that the first coordination shell peak of the pre-catalysis Co-SSP is similar to that of CoCl_2_. After catalysis, the first coordination shell contains local environments similar to those found in CoCl_2_ and Co_3_O_4_, that is, a mixture of Co^2+^ and Co^3+^ ions. This peak also becomes asymmetric, suggesting two types of neighboring atoms located at distinct Fourier-transformed peak distances of 1.45 Å and 1.55 Å. In the second coordination shell, the peak at 2.3 Å gradually increases in intensity from the pre-catalysis sample to the post-catalysis sample, while its position shifts from 2.3 Å before catalysis to 2.4 Å after catalysis. These results indicate that local environments resembling those found in Co_3_O_4_ form during photocatalysis and confirm that Co participates directly in the oxidation process.

Besides H_2_ evolution, the oxidation products from the photocatalytic reforming process were also analyzed using ion chromatography and high-performance liquid chromatography (HPLC). The major oxidation products detected after 22 h were formate and acetate (from pretreated cellulose), as well as glycolaldehyde (GAld) dimer and glycolate (from pretreated PET), respectively, as shown in Fig. 3g,h (Supplementary Table 14). Oxidation of model substrates yielded similar products, with the presence or absence of glycolate from EG depending on the reaction pH (Supplementary Fig. 20 and Supplementary Table 15). The post-photocatalysis solution from the experiments using glucose as the substrate was also analyzed by liquid chromatography–mass spectrometry (LC–MS) and proton nuclear magnetic resonance spectroscopy, revealing key reaction intermediates and products for glucose oxidation (Supplementary Fig. 21). FTIR spectroscopy further confirms that no detectable CO_2_ was produced from the glucose oxidation reaction (Supplementary Fig. 22). Meanwhile, from the EG oxidation products at pH 14 (Supplementary Fig. 20), it is likely that EG oxidation occurs via the known pathway from GAld (which can self-dimerize under the reaction conditions)^36^ to glycolate and finally to formate^37^. Interestingly, formate was absent when pretreated PET was used as a substrate but present when EG was used. This was potentially due to the inhibition of further glycolate oxidation to formate by competitively adsorbed terephthalate on the active sites of the PC^38^.

ICP-OES analyses of the post-catalytic solution showed considerable leaching of the Co co-catalyst (~60%) after 22 h (Supplementary Table 4). To demonstrate the reusability of the sheets, Co-SSP was redeposited onto the used sheets. In glucose, the performance of the sheet was completely restored, indicating the reusability of the Al:SrTiO_3_ layer (Fig. 3i and Supplementary Table 16). Further experiments also show the potential of extending PC sheet stability by the in situ redeposition of leached Co (Supplementary Discussion 4), while the use of a binder and annealing led to only a modest improvement in stability (Supplementary Discussion 5). Conversely, performance was only slightly restored in the pretreated cellulose solution. This was due to the presence of intermediates from cellulose pretreatment consisting of cellobiose and other low degree of polymerization cello-oligosaccharides such as cellotriose and cellotetraose, confirmed by LC–MS^39^ (Supplementary Fig. 23). These intermediates, which are not easily oxidized due to their structural complexity and more stable hydrogen bonding, adsorb onto the PC, as observed by SEM (Supplementary Fig. 24a), thereby poisoning the PC^40^. Nevertheless, there are promising approaches available to enhance the performance and stability of the Al:SrTiO_3_|Co-SSP system using pretreated cellulose, as discussed in detail in Supplementary Discussion 6.

Toward operation under natural sunlight conditions, the performance of Al:SrTiO_3_|Co-SSP_S_ at lower light intensities was also tested. In pretreated cellulose, the PC sheets still exhibited decent performance under low-intensity light irradiation, with the H_2_ evolution rate increasing linearly from 0.01 ± 0.006 to 0.21 ± 0.03 μmol cm^−2^ h^−1^ under 0.1–1.0 Sun irradiation (Supplementary Fig. 25 and Supplementary Table 17). A similar trend was also observed in the oxidation products under the light intensities tested (Supplementary Fig. 26 and Supplementary Table 18). The reusability and low-light-intensity experiments demonstrate that the present PC system is suitable for operation under realistic outdoor conditions. In addition, Al:SrTiO_3_|Co-SSP_S_ maintained their performance at temperatures ranging from 25 °C to 70 °C (Supplementary Fig. 27a and Supplementary Table 19), thus showing that the PC system can operate without a loss in performance under temperatures reasonably expected from solar heating.

### Scale-up of the PC sheets

**Fig. 4:**
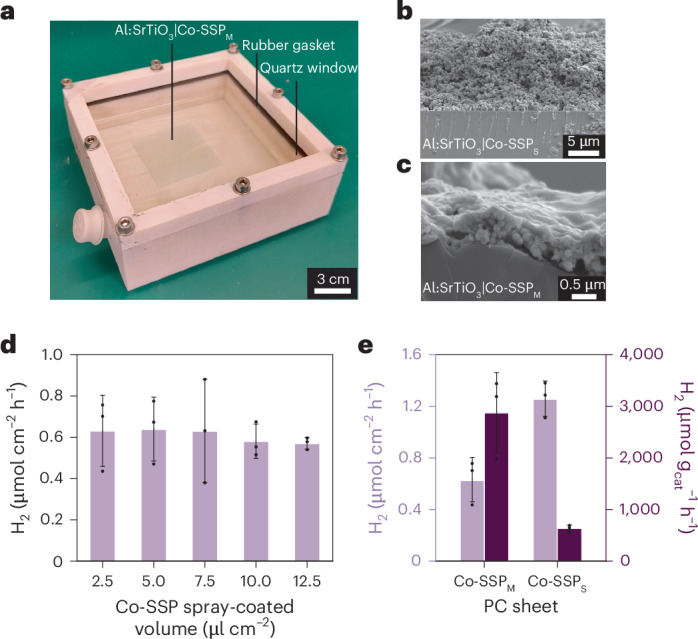
Scale-up of Al:SrTiO_3_|Co-SSP PC sheets. **a**, Photograph of medium-scale photoreactor with a 20.25-cm^2^ Al:SrTiO_3_|Co-SSP_M_ PC sheet. **b**,**c**, Cross-section SEM images of Al:SrTiO_3_|Co-SSP_S_ (**b**) and Al:SrTiO_3_|Co-SSP_M_ (**c**). **d**, Co-SSP spray coating optimization for Al:SrTiO_3_|Co-SSP_M_. **e**, Comparison between H_2_ evolution of Al:SrTiO_3_|Co-SSP_M_ and Al:SrTiO_3_|Co-SSP_S_. Conditions (**d**,**e**): for the medium-scale PC sheets, photocatalytic experiments were performed in 0.1 M TEOA solution under AM1.5G illumination for 6 h at room temperature in an N_2_ atmosphere. The data in **d** and **e** are presented as mean values ± s.d. for reactions performed in triplicate (*n* = 3; individual data points represented as dots).

Before the large-scale application of the PC system, the Al:SrTiO_3_ and Co-SSP deposition procedure was first optimized to allow the facile fabrication of large-area PC sheets. For this purpose, spray coating was employed to deposit both the light absorber and the co-catalyst films onto 20.25-cm^2^ frosted glass sheets to form medium-scale PC sheets (referred to as Al:SrTiO_3_|Co-SSP_M_; see Supplementary Table 2 and the Methods for details). The medium-scale photocatalytic experiments were performed in a custom three-dimensionally printed air-tight top-irradiation-type photoreactor (solution volume 100 ml, headspace ~180 ml) under simulated solar irradiation (AM1.5G, 100 mW cm^‒2^) at room temperature (Fig. 4a and Supplementary Figs. 28 and 29).

Spray coating Al:SrTiO_3_ led to a maximum loading of 0.22 ± 0.01 mg cm^−2^ on the frosted glass sheets. Hence, the Al:SrTiO_3_ layer on Al:SrTiO_3_|Co-SSP_M_ was far thinner than that of Al:SrTiO_3_|Co-SSP_S_ (2 mg cm^−2^ Al:SrTiO_3_) as observed in cross-section SEM images of both sheets (Fig. 4b,c). Nevertheless, UV−vis diffuse reflectance spectroscopy spectra of the PC sheets showed similar light absorption in the <380-nm wavelength region (where Al:SrTiO_3_ absorbs), indicating that the thin light absorber layer on Al:SrTiO_3_|Co-SSP_M_ was still able to fully capture incident light, thus ensuring that sheet performance will not be limited by transmission of light (Supplementary Fig. 30). SEM images of Al:SrTiO_3_|Co-SSP_M_ showed that the sheets had a morphology close to that of the small-scale sheets, with a thicker layer of Co-SSP (Supplementary Figs. 31 and 32). The difference in Co-SSP layer thickness was expected due to the inherent differences in the spin and spray coating deposition techniques and was further confirmed by ICP-OES, which shows a Co and Zr loading of 2.99 and 1.21 wt%, respectively, on Al:SrTiO_3_|Co-SSP_M_ (versus 1.01 and 0.84 wt%, respectively, on Al:SrTiO_3_|Co-SSP_S_; Supplementary Tables 4 and 20).

Interestingly, the H_2_ evolution rate remained in the range of 0.57 to 0.63 μmol cm^−2^ h^−1^ when the Co-SSP spray coating volume was varied from 2.5 to 12.5 μl cm^−2^ (Fig. 4d and Supplementary Table 21), suggesting that only a minimal amount of Co-SSP is required for optimal sheet performance. Hence, further scaled-up PC sheets were fabricated by spray coating only 2.5 μl cm^−2^ of Co-SSP. From Fig. 4e, comparing the performance of Al:SrTiO_3_|Co-SSP_M_ and Al:SrTiO_3_|Co-SSP_S_ showed that the performance of the former was ~50% of the latter on a per area basis (0.63 ± 0.17 and 1.26 ± 0.14 μmol cm^−2^ h^−1^, respectively; Supplementary Table 22). However, the two PC sheets with different fabrication procedures still had similar IQEs at 350 nm when measured using PC sheets of similar area (1 cm^2^; Supplementary Fig. 15 and Supplementary Table 7). Thus, rather than resulting from differences in fabrication procedure, this drop in performance can instead be largely attributed to expected nonlinear changes in reaction kinetics, thermodynamics and fluid mechanics when a chemical system is scaled up^41^. This was supported by experiments scaling up Al:SrTiO_3_|Co-SSP_S_ to 20.25 cm^2^, which showed no meaningful difference in performance compared with Al:SrTiO_3_|Co-SSP_M_ (Supplementary Fig. 33 and Supplementary Table 23). Meanwhile, on a per gram PC basis, Al:SrTiO_3_|Co-SSP_M_ outperformed Al:SrTiO_3_|Co-SSP_S_ with a H_2_ evolution rate of 2870 ± 780 and 630 ± 69 μmol g_cat_^−1^ h^−1^, respectively (Fig. 4e and Supplementary Table 22). Together, these results show that spray coating was suitable as a light absorber and co-catalyst deposition technique for large-scale PC sheet fabrication. While spray coating has been applied in other large-scale PC systems^17^, the use of SSP chemistry allows the co-catalyst layer to be deposited onto the PC sheets without annealing under ambient conditions. Relevant to the outdoor experiments, the effect of temperature on Al:SrTiO_3_|Co-SSP_M_ PC sheet performance was also tested. From 25 °C to 70 °C, there was no noticeable change in H_2_ evolution rate (Supplementary Fig. 27b and Supplementary Table 24).

### Large-scale demonstration under natural sunlight

**Fig. 5:**
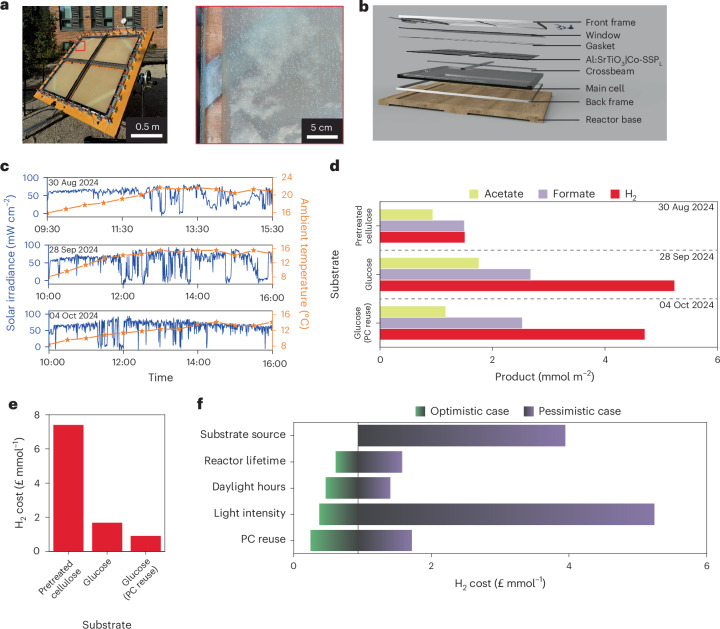
A 1-m^2^ demonstration of the Al:SrTiO_3_|Co-SSP_L_ system under outdoor conditions. **a**, Photographs of the large-scale photoreactor (left) and bubbles produced during the outdoor experiments (right: the photograph is a close-up view of the area bounded by the red box). **b**, Exploded-view schematic diagram of the large-scale photoreactor. **c**, Weather conditions over the course of the 1-m^2^ outdoor demonstrations. **d**, Product formation from the outdoor 1-m^2^ demonstration. **e**, TEA evaluating the cost of H_2_ from the 1-m^2^ outdoor demonstrations. The cost calculated using glucose as a substrate with PC reuse is based on the cumulative results of experiments performed on 28 September and 4 October 2024. **f**, Sensitivity analysis assessing the effect of various parameters on the cost of H_2_. Conditions: the outdoor photocatalytic experiments were performed under natural sunlight for 6 h at ambient temperature and pressure under an N_2_ atmosphere. The substrates used were either glucose or cellulose pretreated by enzymatic hydrolysis using cellulase.

The Al:SrTiO_3_|Co-SSP system was finally employed for large-scale (1 m^2^) outdoor application in a custom-made air-tight panel photoreactor (Fig. 5a and Supplementary Figs. 34 and 35). The structure of the photoreactor is shown and explained in more detail in Fig. 5b and Supplementary Discussion 7. As with the medium-scale sheets, the four large-scale 0.25-m^2^ PC sheets (hereafter referred to as Al:SrTiO_3_|Co-SSP_L_) were fabricated by successive spray coating of Al:SrTiO_3_ and Co-SSP (Fig. 1c and Supplementary Video 1). The outdoor large-scale demonstrations of Al:SrTiO_3_|Co-SSP_L_ were performed within the period of late August to early October 2024 with the incident solar light and ambient temperature during each experiment being recorded in Fig. 5c (variance in incident solar light is presented in Supplementary Table 25). Each experiment involved the use of four Al:SrTiO_3_|Co-SSP_L_ panels for a total active area of 1 m^2^.

Figure 5d shows that 1.51, 1.50 and 0.94 mmol m^−2^ of H_2_, formate and acetate were produced after the 6 h outdoor experiment when pretreated cellulose was used as the substrate, while using glucose yielded as much as 5.24, 2.68 and 1.76 mmol m^−2^ of the respective products (Supplementary Table 26). Photocatalytic performance was expected to be uniform across the Al:SrTiO_3_|Co-SSP_L_ panels based on the uniform absorption of light across the panels (Supplementary Fig. 36). While determining the charge balance between oxidation (formate and acetate) and reduction (H_2_) products is challenging due to the complex nature of glucose oxidation, estimations indicate a relatively closely matched ratio between oxidation and reduction products when using glucose as the substrate (Supplementary Discussion 8). The total organic carbon before and after photocatalysis, calculated based on the substrate glucose and the primary oxidation products formate and acetate, was also determined (Supplementary Table 27). Overall, the performance of Al:SrTiO_3_|Co-SSP_L_ was promising considering the lower intensity and intermittency of natural sunlight (average light intensity over the experiments in Cambridge, United Kingdom was 0.5–0.6 suns, that is, ~50−60 mW cm^−2^). The system worked well in the relatively cold outdoor environment, with the surface of the photoreactor still reaching a temperature of >30 °C as measured by thermal imaging (Supplementary Fig. 37). Bubble formation was also observed over the course of the experiments (Fig. 5a, photograph, Supplementary Video 2 and Supplementary Fig. 38). While the experiments were performed for only 6 h due to limited sunlight availability, it is expected that the performance of Al:SrTiO_3_|Co-SSP_L_ will be maintained for longer periods, as shown in the small-scale stability studies (Fig. 3i), and discussed in Supplementary Discussion 9. Scaling from 1 cm^2^ to 1 m^2^ introduced minor changes in experimental parameters due to reactor design, but these had a negligible impact on PC sheet performance (Supplementary Discussion 10).

The performance of the photocatalytic reforming system was also compared with other reported systems (Supplementary Fig. 39; Supplementary Tables 28 and 29). While the present Al:SrTiO_3_|Co-SSP_L_ system does not appear to provide a substantial improvement over state-of-the-art PC panel systems in terms of areal H_2_ production rate and stability, this gap in performance can be partially accounted for considering that the Al:SrTiO_3_|Co-SSP_L_ system was tested outdoors under low-intensity natural sunlight (intensity ~0.5−0.6 suns, that is, ~50−60 mW cm^−2^) and on a 1 m^2^ scale. These conditions were considerably less favorable than those in most reports, which typically evaluate photocatalytic performance under ideal laboratory conditions on small scales. Despite these conditions, the Al:SrTiO_3_|Co-SSP_L_ PC sheets still maintained a respectable performance, even among reported Al:SrTiO_3_-based systems (Supplementary Discussion 11). In addition, the major improvement of the Al:SrTiO_3_|Co-SSP system over previous reports lies in its ease of panel fabrication, scalability, the use of an earth-abundant transition-element co-catalyst, and the co-production of valuable organics alongside H_2_. Distinct from other PC panel systems, SSP chemistry uniquely allows the fabrication of large PC panels with a noble-metal-free co-catalyst using high-throughput spray coating under ambient conditions without the use of a binder. The production of organics from waste oxidation alongside H_2_ enhances the economic potential and practicality of the present system. Thus, the Al:SrTiO_3_|Co-SSP_L_ PC sheets can be said to compare favorably to existing systems when considering a holistic set of metrics. It is also noted that, although Al:SrTiO_3_|Co-SSP and other photocatalytic reforming systems are thermodynamically more favorable than water splitting, they are still outperformed by benchmark water-splitting systems in terms of H_2_ evolution rate and stability. This probably stems from the complex oxidation pathways and deactivating intermediates formed during organic substrate oxidation, which can hinder overall performance. The use of only the UV-absorbing Al:SrTiO_3_ light absorber also limits the performance of the present Al:SrTiO_3_|Co-SSP system.

### TEA of outdoor 1-m^2^ demonstration

To evaluate Al:SrTiO_3_|Co-SSP_L_ for practical applications, a TEA was conducted to calculate the estimated cost of H_2_ produced (see detailed discussion and calculations in Supplementary Discussion 12 and Supplementary Tables 30–33). The cost of H_2_ when using pretreated cellulose and glucose as a substrate was £7.44 mmol^−1^ and £1.71 mmol^−1^, respectively (Fig. 5e and Supplementary Table 33). The H_2_ cost was further decreased to £0.93 mmol^−1^ when the large-scale experiment was repeated by reusing the Al:SrTiO_3_ panels with redeposited Co-SSP (the cost of H_2_ in this case was calculated based on the cumulative amount produced from the two experiments). As this study represents an empirical TEA on a photocatalytic system based on actual measured data, it should be noted that the cost of H_2_ was found to be higher than previous estimates. This is because previous TEAs were performed based on hypothetical systems with assumed photocatalytic activities and stabilities orders of magnitude higher than any reported systems (Supplementary Fig. 39, gray region, and Supplementary Table 29) and are thus expected to underestimate H_2_ cost. The cost of H_2_ from the current system will be further decreased with larger-scale implementation of the system due to economies of scale, as well as the use of visible-light-absorbing PC systems^42,43^. The valorized oxidation products, which were not included in the preliminary TEA, also add to the economic output of the system. Finally, a sensitivity analysis was conducted to determine the extent to which certain factors affect H_2_ cost from the Al:SrTiO_3_|Co-SSP_L_ system (see detailed discussion in Supplementary Discussion 12; calculations and elaboration on ‘pessimistic’, ‘base’ and ‘optimistic’ cases in Supplementary Tables 34 and 35). Figure 5f shows that light intensity and PC reuse most prominently influences H_2_ cost. Hence, implementation of solar concentration and the development of more stable PC systems will be critical to achieving large-scale H_2_ production at a competitive cost.

## Discussion

Overall, this study demonstrates photocatalytic reforming at a small, medium and large scale using optimized PC sheet assembly procedures comprising a combination of drop casting, spin coating and spray coating to deposit the PC and co-catalyst layers. The solid wastes, cellulose and real-world PET bottles were hydrolyzed by enzymatic and alkaline treatment and reformed to value-added products. The scalability of the PC system was showcased in 1-m^2^ demonstrations of cellulose-derived waste reforming under outdoor conditions. By utilizing simple, high-throughput spray coating, large-scale PC sheets were fabricated under ambient conditions owing to SSP chemistry that allows thin mixed-metal composite co-catalyst films to be deposited without annealing or the use of binders. The SSP approach also represents a step towards bio-inspired manufacturing, providing a route to materials synthesis under mild conditions in contrast to conventional high-temperature, high-pressure processes. The present Al:SrTiO_3_|Co-SSP_L_ PC sheets remain competitive with reported photocatalytic systems without the use of noble metals and demonstrate the use of SSPs in a photocatalytic reforming system.

From the outdoor demonstrations, TEA and sensitivity analyses were performed based on the actual large-scale device with concrete measured data. Although the calculated H_2_ costs obtained were higher than previous reports on idealized photocatalytic systems, the present analyses will be a more useful guide for future research directions as they more accurately portray the current landscape of photocatalysis. Amid rising demand for sustainable H_2_ generation and concerns over improper waste management, the reported Al:SrTiO_3_|Co-SSP_L_ system serves as a potential pathway toward scalable sunlight-driven reforming to tackle both pressing issues.

The systematic scaling up (Fig. 1c, photographs) and performance analysis of the Al:SrTiO_3_|Co-SSP PC sheet systems presented in this work provides important insights into the feasibility of such solar-driven photocatalytic systems for practical applications. In addition to the use of non-precious elements, the versatile and facile fabrication routes employed for the large-scale demonstration of solar-driven waste reforming is an important step forward. Future work in this direction, as revealed from the TEA analysis, would involve improved PC and co-catalyst design, and approaches aiming for better light and thermal management to enhance production rates, thereby making the overall process more ‘photon economical’^44^ and competitive.

## Methods

### Materials

SrTiO_3_ (99.9%, Bioserv), SrCl_2_‧6H_2_O (99.9%, Thermo Fisher Scientific), Al_2_O_3_ nanopowder (<50 nm, Sigma-Aldrich), Na_3_RhCl_6_·*n*H_2_O (17.8 wt% Rh, Mitsuwa), Cr(NO_3_)_3_‧9H_2_O (98.0–103.0%, Kanto), 2-propanol (≥99.5%, Sigma-Aldrich), THF (99.8%, Thermo Fisher Scientific), TEOA (99%, Sigma-Aldrich), glucose (≥99.5%, Sigma-Aldrich), ethylene glycol (≥99%, Sigma-Aldrich), KOH (85%, Thermo Fisher Scientific), Sigmacell cellulose (20 μm, Sigma-Aldrich), cellulase (from *Aspergillus niger*, Sigma-Aldrich), cobalt(II) acetylacetonate (99%, Thermo Fisher Scientific) and zirconium(IV) *n*-propoxide (70 wt% in *n*-propanol, Thermo Fisher Scientific). Solvents were distilled over sodium (toluene) or sodium–potassium amalgam (*n*-hexane) immediately before use.

### Synthesis of the SSP [Co_4_Zr_2_O(O^*n*^Pr)_10_(acac)_4_]

The Co_4_Zr_2_O(O^*n*^Pr)_10_(acac)_4_ SSP complex was prepared by heating Zr(O^*n*^Pr)_4_ (70 wt% in *n*-propanol, 14.4 ml, 32 mmol), Co(acac)_2_ (8.24 g, 32 mmol) and toluene (20 ml) to reflux for 1 h under dry and O_2_-free N_2_, yielding a purple solution. The toluene and *n*-propanol were removed under vacuum and the resulting purple solid was redissolved in *n*-hexane (40 ml) under gentle heating. The solution was stored at ‒30 °C for 16 h resulting in the formation of large cubic purple crystals (6.42 g, 56% yield). Elemental (CHN) analysis calculated (%) for the complex: C_50_H_98_Co_4_O_19_Zr_2_: C 42.2, H 7.0. Found: C 42.1, H 7.0. The complex was dissolved in THF (0.1 M concentration; stock) for the subsequent deposition of co-catalyst films.

### Synthesis of Al:SrTiO_3_

Al:SrTiO_3_ was prepared by using a previously reported flux method^29^. In brief, 1.84, 0.020 and 26.7 g of SrTiO_3_, Al_2_O_3_ nanopowder, and SrCl_2_‧6H_2_O (molar ratio of 1:0.02:10) were mixed using an agate mortar. The mixture was then heated in an alumina crucible at 1423 K for 10 h and subsequently cooled to room temperature, after which the product was stirred in 500 ml of Milli-Q water. The resulting powder was collected by filtration to remove any impurities associated with the SrCl_2_. This rinsing process was repeated three times. The resulting Al:SrTiO_3_ powder was dried at 313 K overnight.

### Deposition and fabrication of Al:SrTiO_3_| Co-SSP_S_ PC sheets

Two milligrams of Al:SrTiO_3_ was dispersed in 200 μl of isopropanol via ultrasonication. A 1.0 cm × 1.0 cm glass sheet was cleaned by ultrasonication for 15 min in acetone, ethanol and water successively. The sheets were then placed in a UV/ozone cleaner for 20 min. The PC dispersion was then drop casted onto the glass sheet in four equal layers (50 μl per layer), with the sheet being allowed to dry between each successive layer. Finally, the sheet was calcined at 573 K for 1 h.

For Co-SSP deposition, a 0.1 M Co-SSP stock solution was first diluted to 0.04 M with THF. Then, 30 μl of the diluted Co-SSP solution was deposited onto the Al:SrTiO_3_ sheets by spin coating at 3,000 rpm for 30 s with a ramp rate of 1,500 rpm s^−1^. The Co-SSP loadings were controlled by spin coating different concentrations of Co-SSP solution without changing the volume used.

### Deposition and fabrication of Al:SrTiO_3_|RhCrO_*x*_ PC sheets

Al:SrTiO_3_ powder was loaded with RhCrO_*x*_ (0.1 wt% Rh and 0.1 wt% Cr) by impregnation from an aqueous solution of 0.01 M Na_3_RhCl_6_ and 0.01 M Cr(NO_3_)_3_, followed by calcination at 623 K for 1 h in air following a previously reported procedure^29^. Two milligrams of the Al:SrTiO_3_|RhCrO_*x*_ PC powder was then drop casted onto a glass substrate following the same procedure as used for the Al:SrTiO_3_|Co-SSP_S_ PC sheets. For the Al:SrTiO_3_|RhCrO_*x*_ PC sheets with spin-coated RhCrO_*x*_, the Na_3_RhCl_6_ and Cr(NO_3_)_3_ solutions were spin coated onto the Al:SrTiO_3_ sheets, followed by annealing at 623 K for 1 h in air.

### Deposition and fabrication of Al:SrTiO_3_|Co-SSP_M_ and Al:SrTiO_3_|Co-SSP_L_ PC sheets

For the fabrication of Al:SrTiO_3_|Co-SSP_M_, 10 mg of Al:SrTiO_3_ was dispersed in 1 ml of isopropanol using ultrasonication. A 4.5 cm × 4.5 cm frosted glass sheet was cleaned by ultrasonication for 15 min in acetone, ethanol and water successively. The PC dispersion was then spray coated onto the frosted glass sheet using a Paasche H-Series Single Action Suction Feed Airbrush (Supplementary Fig. 40). For Co-SSP deposition, 0.1 M Co-SSP stock solution was first diluted to 0.04 M with THF. 50 μl of the Co-SSP solution was then spray coated onto the Al:SrTiO_3_ sheet at room temperature in an air atmosphere. The fabrication of Al:SrTiO_3_|Co-SSP_L_ was done by successive spray coating of Al:SrTiO_3_ and Co-SSP onto 50 cm × 50 cm frosted glass panels similar to the procedure described for the Al:SrTiO_3_|Co-SSP_M_ PC sheets.

### Materials characterization

A TESCAN MIRA3 FEG-SEM instrument with an Oxford Instruments Aztec Energy X-Max^N^ 80 system was used to collect the SEM images and corresponding EDX mapping. A Thermo Scientific Talos F200X G2 transmission electron microscope (operating voltage 200 kV) equipped with a Thermo Scientific Ceta camera was used for TEM imaging, bright-field scanning TEM detector and a Super-X detector system for EDX mapping. Solution UV–vis spectra were obtained using an Agilent Cary 60 UV–vis spectrophotometer using quartz cuvettes with 10 mm path length, while UV–vis diffuse reflectance spectra were obtained using a Harrick Scientific Video Barrelino probe. FTIR spectra were recorded on a Thermo Scientific Nicolet iS50 spectrometer. ICP-OES was carried out on a Thermo Scientific iCAP 700 spectrometer, and elemental CHN analysis was obtained using a PerkinElmer 240 Elemental Analyser or an Exeter Analytical CE-440 Elemental Analyser by the University of Cambridge (Department of Chemistry) Microanalysis Service. High-resolution mass spectra were recorded by electrospray ionization mass spectrometry on a Thermo Fisher Q-Exactive Mass Spectrometer with an electrospray ionization source by direct injection in negative ion mode. XRD analysis was conducted using a Panalytical X’Pert Pro powder X-ray diffractometer by the University of Cambridge X-ray laboratory.

Co K-edge XAS was performed at the B18 Core XAS beamline^45,46^ of Diamond Light Source (experiment ID: SP42410). The pre- and post-catalysis samples were mixed with cellulose (1:6 ratio) and made into 10-mm pellets. The external reference samples CoCl_2_ and Co_3_O_4_ were also prepared in the same way. The setup utilized Pt-coated collimating, focusing and harmonic-rejection mirrors, along with an Si(111) double-crystal monochromator for incident X-ray energy selection. A Co foil reference was recorded simultaneously for energy calibration. Measurements were carried out in Quick EXAFS (QEXAFS) mode at room temperature. Pre-catalysis samples and CoCl_2_ and Co_3_O_4_ reference compounds were measured in transmission mode, while the post-catalysis sample was measured in fluorescence mode using a Vortex ME 7 Silicon drift detector coupled with an Xspress-3X digital pulse processor. The post-catalysis sample was measured in fluorescence mode because the remaining material after catalysis was insufficient for transmission measurements. Each sample that was measured in transmission mode was scanned three times, whereas the post-catalysis sample underwent 15 scan repetitions to improve the signal-to-noise ratio and ensure spectral reproducibility. Data processing and analysis were conducted with the Demeter software suite^47^.

XPS analysis was performed using a Thermo Scientific Escalab 250Xi fitted with a monochromated Al Kα X-ray source (1486.7 eV). All data were recorded with an X-ray beam size of 650 µm and a pass energy of 20 eV at a step size of 0.1 eV. Electronic charge neutralization was achieved using an ion source with ion gun current and voltage of 100 µA and 40 V, respectively. All sample data were recorded at a pressure below 10^−8^ torr and a room temperature of 294 K. TGA was performed on a Mettler Toledo TGA/DSC 2 STARe System. A sample of 10−20 mg was heated to 800 °C at a rate of 10 °C min^−1^. Measurements on samples were performed under a constant flow (80 ml min^−1^) of air (19−22% O_2_ in N_2_, <10 ppm H_2_O), provided by Air Liquide UK Limited. X-ray crystallographic data were collected using a Nonius KappaCCD (Mo Kα) diffractometer equipped with an Incoatec IμS microsource (Cu Kα). The temperature was held at 180 K using an Oxford Cryosystems N_2_ cryostat. Data integration and reduction were undertaken with HKL Denzo/Scalepack (Nonius). Multiscan corrections were applied using SORTAV (Nonius). Structures were solved using SHELXT and refined using SHELXL. Details of experimental acquisition, data processing and crystallographic parameters are presented in Supplementary Table 1 (the CCDC no. of the Co-Zr SSP X-ray structure is 2416182).

### PEC characterization

Chronoamperometry and PEIS experiments were carried out in an electrochemical cell using a three-electrode configuration consisting of an Al:SrTiO_3_ working electrode, a Pt mesh counter electrode and a RE-6 Ag/AgCl reference electrode (3 M NaCl gel, 0.55 mm diameter ceramic frit, MW-2030, BASi). The 30 ml stirred N_2_-saturated electrolyte contained 0.1 M Na_2_SO_4_ and 0.1 M substrate (TEOA, glucose or EG). The Al:SrTiO_3_ working electrodes were made by drop casting an Al:SrTiO_3_ dispersion (2 mg Al:SrTiO_3_ in 200 μl isopropanol) onto fluorine-doped tin-oxide-coated glass. The loading of SSP onto the working electrode was achieved by spin coating Co-SSP onto the electrode following the same procedure as Al:SrTiO_3_|Co-SSP_S_ PC sheet fabrication. A 500 W xenon arc lamp (Newport 67005) solar light simulator that was calibrated to AM1.5G was used as the light source. Chronoamperometry was conducted under chopped light irradiation in the presence of TEOA. The impedance response was recorded under continuous irradiation using a potentiostat (IviumStat) with frequency ranges from 1 MHz to 0.5 Hz and a 15-mV sinusoidal amplitude. Impedance data were fitted using the Randles equivalent circuit implemented in the modeling software ZView2 (Scribner Associates). The Randles circuit consists of a series resistor (*R*_S_), a charge transfer resistor (*R*_ct_) and a capacitor describing the capacitance across the semiconductor–liquid junction (*C*_SCLJ_). To account for the nonideal capacitive behaviors of the photoelectrodes, which may arise from surface inhomogeneity and frequency-dependent dielectric properties, *C*_SCLJ_ was replaced by a constant phase element (CPE) during fitting. No Warburg element was included in the Randles circuit because the PEIS Nyquist plots are dominated by a large semicircle at low frequencies, without the appearance of a linear tail that would indicate diffusion-controlled impedance.

### Calculation of IQE

The apparent quantum yield (AQY) and IQE were calculated from photocatalytic H_2_ evolution experiments using equations (1) and (2).

1 $$\begin{array}{l}{\rm{AQY}}\,({ \% })=\displaystyle \frac{{\rm{Number}}\,{\rm{of}}\,{\rm{reacted}}\,{\rm{electrons}}}{{\rm{Number}}\,{\rm{of}}\,{\rm{incident}}\,{\rm{photons}}}\times 100 \\ \qquad\qquad\;\;=\displaystyle \frac{{\rm{Number}}\,{\rm{of}}\,{\rm{evolved}}\,\,{{\rm{H}}}_{2}\,{\rm{molecules}}\times 2}{{\rm{Number}}\,{\rm{of}}\,{\rm{incident}}\,{\rm{photons}}}\times 100\end{array}\,$$

2 $$\begin{array}{l}{\mathrm{IQE}}\,({ \% })=\displaystyle\frac{\mathrm{AQY}}{A}\times 100 =\displaystyle\frac{{\mathrm{Number}}\,{\mathrm{of}}\,{\mathrm{evolved}}\,\,{{\rm{H}}}_{2}\,{\mathrm{molecules}}\times 2}{{\mathrm{Number}}\,{\mathrm{of}}\,{\mathrm{incident}}\,{\mathrm{photons}}\times {\rm{A}}}\\ \,\,\,\,\,\,\,\,\,\,\,\,\,\,\,\,\,\,\,\,\,\,\,\,\,\times 100 ,\end{array}$$

where AQY is calculated from the incident photons and H_2_ yield, and *A* is the absorption of the PC sheet.

### Pretreatment of cellulose and real-world PET bottles

Cellulose was pretreated using the enzyme cellulase following a previously reported procedure^48^. In brief, a suspension of 2.5 g of cellulose in 50 ml of 0.1 M aqueous NaHCO_3_ solution at pH 6.5 (adjusted by the addition of HCl) was maintained at 37 °C. Then, 0.5 g of cellulase in 50 ml of 0.1 M aqueous NaHCO_3_ solution at pH 6.5 (adjusted by the addition of HCl) was added to the cellulose suspension. The suspension was incubated at 37 °C for 24 h under stirring, followed by filtration through a syringe filter (0.2 μm) to remove unreacted cellulose. The filtered solution was stored at −4 °C before use. The solution contained 0.011 M of glucose.

The real-world PET plastic water bottle was pretreated using an alkaline hydrolysis method^49^. In brief, the PET bottle was first cut into small pieces and dipped in liquid nitrogen. Thereafter, the pieces were shredded using a grinder. The ground PET bottle was then added to 1.0 M aqueous KOH at a concentration of 50 mg ml^−1^ and heated to 80 °C for 3 days under stirring to ensure sufficient depolymerization of the PET. The solution was then kept unperturbed at room temperature to cool and finally filtered through a syringe filter (0.2 μm) to remove unreacted PET. The solution contained 0.13 M of EG.

### Photocatalytic reforming experiments at small (1 cm^2^) and medium (20.25 cm^2^) scales

Small-scale photocatalytic measurements were conducted in a top-irradiation-type glass photoreactor with a screw-top cap equipped with a quartz window (Supplementary Fig. 10). For photocatalytic reforming experiments, 10 ml of the chosen feedstock (0.1 M TEOA, 0.1 M glucose, 0.1 M EG, pretreated cellulose or pretreated PET) was added to the photoreactors. The bulk solution for the experiments performed at pH 7 and 14 were ultrapure water and 1.0 M KOH, respectively. The prepared PC sheets were then placed into the photoreactors. The liquid and photoreactor headspace were purged with N_2_ (containing 2% CH_4_ for GC analysis) for 30 min. All purging needle holes were then sealed with Loctite Superglue Universal adhesive. The sheets were then irradiated by a solar light simulator (G2V Sunbrick LED Solar Simulator) calibrated to match air mass 1.5 global (AM1.5G) irradiance. The experiments were conducted without stirring at room temperature.

Medium-scale photocatalytic measurements were conducted in a custom three-dimensionally printed top-irradiation-type photoreactor equipped with a quartz window (Supplementary Figs. 28 and 29). For optimization of the medium-scale PC sheets, 100 ml of 0.1 M TEOA was added to the photoreactor. The prepared PC sheet was then placed into the photoreactor. The lid of the photoreactor was secured using eight M4 screws with a rubber gasket to provide an airtight seal. The liquid and photoreactor headspace were purged with N_2_ and subsequently irradiated by a solar light simulator similar to the small-scale experiments. The experiments were also conducted without stirring at room temperature.

### Large-scale (1 m^2^) photocatalytic reforming under outdoor conditions

For the large-scale experiments, four Al:SrTiO_3_|Co-SSP_L_ panels were placed into a custom-built panel photoreactor (Supplementary Figs. 34 and 35; a detailed description of the photoreactor and 1-m^2^ demonstration is provided in Supplementary Discussion 7). The photoreactor was clamped shut and filled with 20 l of the chosen feedstock (0.011 M glucose or pretreated cellulose) using a submersible water pump. The liquid and photoreactor headspace were purged with N_2_ (containing 2% CH_4_ for GC analysis) for 30 min via tubing installed at the bottom and top of the photoreactor.

The photoreactor was then placed under natural sunlight for 6 h. The photoreactor was tilted and rotated to track the Sun while incident light intensity was continuously measured using a Newport 843-R-USB Handheld Power Meter equipped with a 919P-020-12 Thermopile sensor. Gas products were collected in a separate gas collection chamber connected to the headspace of the photoreactor while liquid products were collected by drawing liquid samples from the photoreactor. The total organic carbon before and after reaction was calculated with respect to the substrate glucose as well as the primary oxidation products formate and acetate.

### Product analysis

GC was used to measure H_2_ evolution. Fifty microlitres of gas samples were periodically taken from the headspace of the photoreactors and analyzed using a Shimadzu GC-2010 Plus gas chromatograph with a barrier discharge ionization detector (2% CH_4_ was used as an internal standard). The concentration of produced H_2_ in the photoreactor headspace can be calculated using

$${\mathrm{Concentration}}_{{H}_{2}}=\frac{{\mathrm{Area}}_{{H}_{2}}}{{\mathrm{Area}}_{{{CH}}_{4}}}\times \frac{{\mathrm{Concentration}}_{{{CH}}_{4}}}{{\mathrm{Response}\,\mathrm{factor}}_{{H}_{2}}}$$

where ${\mathrm{concentration}}_{{\mathrm{H}}_{2}}$ is the concentration of H_2_ in the photoreactor headspace, ${\mathrm{area}}_{{\mathrm{H}}_{2}}$ and ${\mathrm{area}}_{{\mathrm{CH}}_{4}}$ are the peak areas of H_2_ and CH_4_, respectively, obtained from GC sampling, ${\mathrm{concentration}}_{{\mathrm{CH}}_{4}}$ is the concentration of CH_4_ internal standard in the photoreactor headspace (2%) and ${\mathrm{response}\,\mathrm{factor}}_{{\mathrm{H}}_{2}}$ is a constant calculated from the peak area and concentration ratios of samples with known composition of H_2_ and CH_4_.

Formate and acetate concentrations were determined using ion-exchange chromatography on a Metrohm 882 compact ion-exchange chromatography system equipped with a Metrosep A Supp 5-150/4 column using 3.2 mM aqueous Na_2_CO_3_ and 1 mM aqueous NaHCO_3_ as the eluent. The response factors of formate and acetate were determined by calibration with known amounts of aqueous formate and acetate solutions. Glucose, EG, GAld dimer and glycolic acid concentrations were determined by HPLC on a Waters Breeze system equipped with a RIS-2414 refractive index detector and a Rezex 8% Ca^2+^ monosaccharide 300 × 7.80 mm HPLC column (for glucose) and a Rezex ROA-Organic Acid H^+^ 300 × 7.80 mm HPLC column (for EG, GAld dimer and glycolic acid) using 2.5 mM H_2_SO_4_ as the eluent with a flow rate of 0.4 ml min^−1^ at 75 °C. The response factors for the substrates were determined by calibration of aqueous solutions with known amounts of the respective substrates.

### Statistics

Average values are based on measurements performed in triplicates, except for the large-scale experiments, where the values are based on measurements performed as a single experiment. The errors correspond to the standard deviation (s.d.) of data points from individual samples.

## Supplementary information

Supplementary Information Supplementary Discussions 1–12, Figs. 1–61 and Tables 1–49.

Supplementary Video 1 Video of catalyst spray coating on large-scale PC sheets.

Supplementary Video 2 Video of bubble evolution during large-scale outdoor experiment.

## Source data

Source Data Figs. 1–5 Source data for Figs. 1–5.

## Data Availability

The data that support the findings of this study are available via the University of Cambridge data repository at 10.17863/CAM.129936.
