# Supplementary material for: Photoreforming of solid waste on 1 m2 scale using single-source precursor-derived co-catalyst films
Source: Nat Chem Eng. 2026 Jun 24;3(6):351–62. doi: 10.1038/s44286-026-00406-y (PMC13293892; doi:10.1038/s44286-026-00406-y)
Supplement: Supplementary file 1 — Supplementary Discussions 1–12, Figs. 1–61 and Tables 1–49. [file 44286_2026_406_MOESM1_ESM.pdf]

nature chemical engineering

Supplementary information https://doi.org/10.1038/s44286-026-00406-y

Photoreforming of solid waste on 1 m^2 scale using single-source precursor-derived co-catalyst films

In the format provided by the authors and unedited

Table of Contents

Supplementary Discussions 1−12	Page S2 to Page S11
Supplementary Figures 1−61	Page S12 to Page S46
Supplementary Tables 1−49	Page S47 to Page S66
Supplementary References	Page S67 to Page S72

Supplementary Discussion 1 | Rationale for photocatalytic reforming substrate selection and waste substrate pre-treatment.

The model photocatalytic reforming substrates chosen were glucose (model substrate of cellulose – a major fraction of lignocellulosic biomass) and ethylene glycol (EG; model substrate of polyethylene terephthalate (PET) plastics). The valence and conduction band positions of Al:$SrTiO_3$ align well with the oxidation potential of these substrates and the reduction potential of H_2 evolution, confirming that the photocatalyst (PC) is able to drive these chemical reactions (Supplementary Fig. 41 and Supplementary Table 36). The photocatalytic reforming tests were carried out in aqueous media under neutral pH for glucose (pH ~7) as near-neutral conditions are used for pre-treating cellulose by enzymatic digestion. Conversely, the experiments were carried out under both neutral (pH ~7) and alkaline (pH ~14) conditions for EG as alkaline conditions are typically used for pre-treating PET plastics by alkaline hydrolysis. Thus, the conditions of these experiments using model substrates correspond to those of the pre-treated solid waste.

For the pre-treatment of waste-derived substrates, cellulose was pre-treated using the enzyme cellulase to yield glucose (0.011±0.001 M glucose from 0.05 mg ml^{-1} cellulose), as detected using high-performance liquid chromatography, in near-neutral aqueous buffer conditions (0.1 M $NaHCO_3$, pH 6.5; see Methods section for details) as demonstrated in previous reports.[1] PET, on the other hand was pre-treated by alkaline hydrolysis in 1.0 M aqueous KOH to yield its constituent monomers, EG (0.13±0.01 M) and terephthalate (see Methods section for details).[2] The solution obtained after pre-treatment of the polymeric substrates was used directly for photocatalytic reactions.

Supplementary Discussion 2 | Comparison between Al:$SrTiO_3$|Co-SSP and benchmark Al:$SrTiO_3$|$RhCrO_x$ systems.

The performance of the Al:$SrTiO_3$|Co-SSP_S PC sheets was compared to that of Al:$SrTiO_3$ PC sheets with a benchmark $RhCrO_x$ co-catalyst (hereafter referred to as Al:$SrTiO_3$|$RhCrO_x$).[3] The amount of H_2 produced in the presence of glucose using Al:$SrTiO_3$|Co-SSP_S sheets was comparable to that using the benchmark Al:$SrTiO_3$|$RhCrO_x$ PC (Supplementary Fig. 42a and Supplementary Table 9), clearly indicating the potential of co-catalyst films (based on earth-abundant transition elements) emerging from unique SSP chemistry to replace expensive rare earth-based mixed-metal co-catalysts, such as the previously used $RhCrO_x$ co-catalyst.[4,5] It is also noted that the $RhCrO_x$ co-catalyst leached to a lesser extent than the Co-SSP co-catalyst after photocatalytic reactions (~10% leaching of the Rh co-catalyst for the benchmark Al:$SrTiO_3$|$RhCrO_x$ system compared to ~60% leaching of the Co co-catalyst for the Al:$SrTiO_3$|Co-SSP_S system; Supplementary Table 37). However, while Co contamination of liquid products can potentially complicate product recovery, many effective techniques are already available to recover and remove Co from aqueous streams, which can be potentially reused in the Al:$SrTiO_3$|Co-SSP system.[65] Additionally, Al:$SrTiO_3$|$RhCrO_x$ performed worse in the presence of organic substrates than during pure water oxidation (Supplementary Fig. 43), in agreement with previous literature.[50] This contrast further highlights the advantage of the Al:$SrTiO_3$|Co-SSP system in achieving enhanced H_2 evolution when coupled to waste reforming.

For the waste-derived substrates, while the Al:$SrTiO_3$|Co-SSP_S PC sheets were outperformed by the benchmark Al:$SrTiO_3$|$RhCrO_x$ with the pre-treated PET substrate, their H_2 evolution rates were still comparable with the pre-treated cellulose substrate (0.21±0.03 and 0.29±0.02 μmol cm^{-2} for the former and latter, respectively; Supplementary Fig. 42b and Supplementary Table 13). The relatively larger gap in performance between Al:$SrTiO_3$|Co-SSP_S and Al:$SrTiO_3$|$RhCrO_x$ in the 1.0 M KOH pre-treated PET was attributed to the higher intrinsic H_2 evolution activity of Rh co-catalysts (Cr species in the $RhCrO_x$-loaded system only prevents backward reaction and does not directly enhance H_2 evolution)[47] in alkaline conditions compared to Co co-catalysts, which was not a factor in the near-neutral cellulase pre-treated cellulose.[59–61] Nevertheless, when using the same co-catalyst deposition technique (spin coating) for both Al:$SrTiO_3$|Co-SSP_S and Al:$SrTiO_3$|$RhCrO_x$, the gap in performance between the two systems with the pre-treated PET substrate was much smaller, while Al:$SrTiO_3$|Co-SSP_S actually outperforms Al:$SrTiO_3$|$RhCrO_x$ by ~4× with the pre-treated cellulose substrate (Supplementary Fig. 44 and Supplementary Table 38).

On the oxidation side, the same distribution of products was obtained from the Al:$SrTiO_3$|$RhCrO_x$ sheets as the Al:$SrTiO_3$|Co-SSP_S sheets for both the model and waste-derived substrates (Supplementary Figs. 45 and 46; Supplementary Tables 14 and 15).

Supplementary Discussion 3 | Co-SSP as a co-catalyst for the Al:$SrTiO_3$|Co-SSP PC sheets.

Co-SSP was confirmed to act as an H_2 evolution co-catalyst. However, it is expected that only the Co species is responsible for facilitating H_2 evolution reaction (HER) while Zr species remains relatively inert and do not participate in the redox process, as demonstrated from control experiments using CoO and ZrO_2 metal oxides as co-catalysts for Al:$SrTiO_3$ where only the PC sheets with CoO showed photocatalytic activity (Supplementary Fig. 47 and Supplementary Table 39). Nevertheless, the Zr component plays a necessary role in SSP synthesis providing a robust host for the Co species. Note that the experiments using metal oxides as co-catalysts required a Nafion binder for effective attachment of these co-catalysts onto the Al:$SrTiO_3$ sheet. This highlights another advantage of utilising SSP chemistry for forming co-catalyst films on PC sheets, which is the formation of films under ambient conditions (that is, without high-temperature annealing) without the use of binders. For comparison, Al:$SrTiO_3$ sheets fabricated with a Co salt as co-catalyst without annealing and without the use of a binder had a H_2 evolution rate >10× lower than that of the Al:$SrTiO_3$|Co-SSP_S PC sheets (Supplementary Fig. 48 and Supplementary Table 39).

Aside from the role of Co-SSP as a H_2 evolution co-catalyst, it is also noted that linear sweep voltammetry (LSV) curves (Supplementary Fig. 49) reveal Co-SSP also serves as a co-catalyst for glucose and EG oxidation reactions across different pH environments. This highlights another advantage of Co-SSP as a co-catalyst for photocatalytic waste oxidation, which is a highly promising aspect considering the very limited availability of such co-catalysts.

Supplementary Discussion 4 | In-situ redeposition and recycling of leached Co to enhance stability of Al:$SrTiO_3$|Co-SSP system.

An in-situ Co recycling and redeposition strategy was employed by performing photocatalytic reactions in substrate solutions containing a low concentration of Co^{2+} ions. This strategy has been demonstrated in previous reports to extend the stability of Co catalyst films by continuously redepositing Co species over the course of the reaction.[6,7] However, as the addition of Co^{2+} ions from fresh chemicals into the reaction solution is undesirable from a practical standpoint, Co^{2+} ions leached from the PC sheets were instead utilised, allowing for the simultaneous recycling of leached Co and enhancement of PC sheet stability. This was done by first performing the photocatalytic reforming reaction under standard conditions, followed by using the same reaction solution (containing Co^{2+} ions leached from the first PC sheet) for a second photocatalytic cycle with a fresh PC sheet.

The presence of low concentrations of Co^{2+} ions (~0.1 mM) in the reaction solution led to noticeably improved PC sheet stability (Supplementary Fig. 50 and Supplementary Table 40). The long-term experiments showed that the PC sheets retained ~50% of their performance in the last 22 h compared to the first 22 h. This was in contrast to the long-term experiments without Co^{2+} ions present in the reaction solution where almost all performance was lost after 22h (Supplementary Fig. 51, top panel; Supplementary Table 41). The enhanced stability in the presence of Co^{2+} ions was likely due to in-situ redeposition of Co species on the PC sheets over the course of the photocatalytic experiment, as indicated by inductively coupled plasma optical emission spectroscopy (ICP-OES) measurements showing a high loading of 0.86 wt% Co species (versus 1.01 wt% for the as-prepared sheets) still present on the PC sheet even after 66 h of operation (Supplementary Table 42). With this configuration, the feasibility of the Al:$SrTiO_3$|Co-SSP system in batch mode is significantly improved as retaining leached Co in the reaction solution will be beneficial for the long-term stability of the PC sheets, allowing for the mitigation of the drawback of the system regarding Co leaching. It is noted that Zr^{4+} ions were also present in the reaction solution as Zr was also leached from the PC sheets. However, control experiments in the presence of only Zr^{4+} ions showed no improvement in long-term photocatalytic performance (Supplementary Fig. 52; Supplementary Table 42 and 43), as was expected considering that only Co acted as the H_2 evolution co-catalyst.

Supplementary Discussion 5 | Effect of binder and annealing on the performance of the Al:$SrTiO_3$|Co-SSP system.

A Nafion binder and annealing were also tested to improve the long-term performance of the PC sheets (Supplementary Fig. 51 and Supplementary Table 41). While the binder did little to improve performance (and potentially inhibited H_2 desorption from the sheets as shown in Supplementary Fig. 53), annealing the sheets allowed them to retain ~30% of their performance even after 66 h without Co-SSP re-deposition as Co leaching from the PC sheets was decreased (Supplementary Table 44). Additionally, in all cases there was no significant leaching of Al, Sr and Ti species, confirming the high stability of the metal oxide light absorber (Supplementary Table 44).

Supplementary Discussion 6 | Approaches to enhance the performance and stability of the Al:$SrTiO_3$|Co-SSP system for the photocatalytic reforming of pre-treated cellulose.

The enzymatic treatment of cellulose using cellulase yields a solution containing glucose and intermediate products cellobiose and other low degree of polymerisation cello-oligosaccharides such as cellotriose, cellotetraose, etc., which can poison the PC.[8] This leads to poor photocatalytic performance and stability when using pre-treated cellulose compared to glucose.

To overcome this issue, the Al:$SrTiO_3$|Co-SSP system presented in this work can be paired with thermal or photothermal approaches to more efficiently reform real-world cellulose and other polymeric substrates. The heat-assisted photocatalytic oxidation of sugars and polyols to liquid organic H_2 carriers such as formic acid, which is subsequently photocatalytic converted to H_2 with high yields have already been demonstrated.[9] This stepwise waste oxidation and H_2 generation concept has great potential for integration with large-scale photocatalytic reforming systems, such as in this work, to improve performance and stability. Solar heating using lenses or reflectors to concentrate sunlight can also be integrated into these systems to realise a fully solar-driven cellulose reforming process.

Along similar lines, the cellulose pre-treatment method can be optimised to obtain a more glucose-rich solution with less intermediates that are detrimental to the photocatalytic reforming performance. Enzymatic treatment was used in this work as it allowed for the hydrolysis of cellulose relatively easily under mild conditions. Nevertheless, there are other approaches for cellulose hydrolysis worth considering. Acid hydrolysis of cellulose is potentially the most developed process as it is inexpensive and fast.[10] However, due to challenges in neutralisation of the acid, solid acid catalysts have more recently gained attention. These catalysts can convert cellulose to glucose more efficiently than enzymatic processes,[11] and are thus promising for pre-treating cellulose to minimise intermediates detrimental to photocatalyst performance prior to the photocatalytic reforming reaction.

More generally, there have also been efforts to develop photocatalytic systems for reforming polymeric substrates, rather than monomeric glucose.[12,13] These photocatalytic systems more efficiently reform cellulose and its derivatives by first producing hydroxyl radicals to break the glycosidic linkages of these polymeric substrates to form glucose, xylose, formic acid, hydroxymethyl furfural, etc., which are more easily oxidised.[14–16] Insights from these studies can be applied to the present Al:$SrTiO_3$|Co-SSP system to optimise the oxidation side of the overall photocatalytic reforming reaction by introducing an oxidation co-catalyst or tailoring the structure of the PC system.

Supplementary Discussion 7 | Large-scale photoreactor and 1 m^2 demonstrations under outdoor conditions.

The outdoor demonstrations were performed in a custom-built panel photoreactor. The main cell of the photoreactor, in which the photocatalytic reactions take place, was constructed from acrylic with a UV-transparent window. An inner acrylic crossbeam provides internal support to the main cell. This internal crossbeam also holds four 0.25 m^2 PC panels in place during photoreactor operation, giving a total active area of 1 m^2 (Fig. 5b and Supplementary Figs. 34 and 35). An outer aluminium frame provides additional structural stability to the cell. The main

cell was sealed and held onto a timber base by toggle clamps, with a compressed neoprene gasket along the edge of the cell forming a gastight seal during operation. The photoreactor can also be tilted and rotated to allow for solar tracking and is equipped with liquid and gas inlets and outlets for gas purging, filling of reaction solution and sample collection.

During the reactions, the main cell was fully filled with the reforming feedstock solution. The main cell is connected to a smaller gas collection chamber (volume of ~2.7 l) in which produced H_2 gas was collected. The gas collection chamber was equipped with a rubber septum for gas sampling and purged with N_2 containing 2% CH_4 as an internal standard before starting the experiments. Gas samples were withdrawn from this gas collection chamber periodically and analysed using gas chromatography following the same procedure as the lab-scale experiments (see Methods section for details on gas chromatography measurements). The headspace volume of the main cell (fully filled with substrate solution) was negligible compared to that of the gas collection chamber. This ensures that product H_2 would be collected in the collection chamber, rather than accumulating in the main cell due to the pressure difference between the main cell and collection chamber. Furthermore, as H_2 is the lightest gas, it diffuses rapidly throughout the gas collection chamber headspace and quickly equilibrates concentration gradients in the headspace. Consequently, the generated H_2 is expected to mix throughout the headspace on a short timescale. The gas in the headspace of the collection chamber was also agitated by drawing the gas in and out of the syringe several times before collecting the gas sample. These factors ensure that the distribution of H_2 in the collection chamber is homogeneous.

To identify any potential gas leakage from the 1 m^2 photoreactor over the course of the outdoor experiments, the gas collection chamber headspace was sampled at the beginning of the experiment and the peak areas of the CH_4 internal standard was compared to those at the end of the experiments (sample gas chromatograms provided in Supplementary Fig. 54). It can be seen that there was a small decrease of ~16% in CH_4 area, indicating that there was slight leakage over the course of the experiment. This was expected as it is challenging to completely prevent gas leakage in large reactors. Nevertheless, the inclusion of the CH_4 internal standard allows for gas leakage to be accounted for as product H_2 quantification is performed with respect to the amount of CH_4 standard gas present. It is also noted that this decrease in CH_4 area is also partially accounted for by pressure buildup from produced H_2 considering the relatively small volume of the sample collection chamber, meaning that there was likely significantly less leakage than indicated by the CH_4 area drop. A relatively large increase in H_2 peak area can be seen by the end of the large-scale experiment (Supplementary Fig. 54). This confirms that a significant amount of H_2 gas was formed during the experiment, and that the reported photocatalytic performance of the large-scale system was not influenced by gas collection or sampling artefacts.

The 1 m^2 demonstrations were performed beside the Yusuf Hamied Department of Chemistry building in Cambridge between late August to early October 2024. During the demonstrations, the position of the sun was tracked with continuous measurement of incident light intensity (Fig. 5c). The feedstocks selected for the 1 m^2 demonstrations were pre-treated cellulose and 0.011 M glucose (equivalent to the concentration of glucose obtained from cellulose pre-treatment by cellulase). Pre-treated cellulose was selected as a feedstock to more accurately emulate a potential real-world waste reforming scenario. Enzymatic treatment of cellulose with cellulase was, in turn, selected as it allows for the breakdown of cellulose under mild conditions compared to methods such as acid hydrolysis, which require either concentrated acids or high temperatures and pressures.[17–21] Cellulase production in large scales have also been demonstrated,[22,23] further making it a realistic choice for waste pre-treatment.

However, the photocatalytic performance of the Al:$SrTiO_3$|Co-SSP_L system was drastically decreased due to fouling from the pre-treated cellulose solution. Hence, a dilute glucose solution was also used as a feedstock to maximise photocatalytic performance. The concentration of glucose used was 0.011 M, equal to that obtained from cellulose pre-treatment. The use of dilute glucose was to improve the economic aspects of the photocatalytic system, as small-scale experiments indicate that H_2 evolution was similar regardless of whether relatively high or low concentrations of glucose were used (Supplementary Fig. 24b; Supplementary Table 45). The difference in performance when using the two feedstocks is discussed in the context of a technoeconomic analysis in Supplementary Discussion 12.

Supplementary Discussion 8 | Charge balance between oxidation and reduction products from 1 m^2 outdoor experiments.

The oxidation of glucose to formate involves the transfer of 2 electrons per molecule of produced formate.[24] Hence, a 1:1 product ratio of formate and H_2 is expected, which accounts for about half of the H_2 produced from the 1 m^2 outdoor experiments using glucose as the substrate (Figure 5d). On the other hand, it is challenging to determine the expected product ratio of acetate and H_2 as literature on glucose oxidation to acetate is scarce (acetate is typically only a relatively minor product). Nevertheless, considering that the complete oxidation of glucose to water and CO_2 requires the transfer of 24 electrons (thus the partial oxidation of glucose to acetate should involve much fewer electron transfer),[25] the total ratio between oxidation and reduction products from the 1 m^2 experiments using glucose as the substrate can still be expected to relatively closely match.

On the other hand, when using pre-treated cellulose as the substrate, there was a more noticeable difference in product ratios where the evolved H_2 was lower than expected. This may be due to increased gas trapping by the pre-treated cellulose solution compared to the glucose solution. Aside from glucose, the pre-treated cellulose solution also contained intermediate products of cellulose hydrolysis consisting of cellobiose and other low degree of polymerisation cello-oligosaccharides (LD-COS) such as cellotriose, cellotetraose, etc.[8] The presence of cellobiose and these LD-COS was verified using LC−MS (Supplementary Fig. 23). These higher molecular weight polymers increase the viscosity of the pre-treated cellulose solution, thus restricting the flow of product H_2 gas bubbles from the solution to the reactor headspace.[26,27] Considering the large volume of solution in the large-scale outdoor experiments, the volume of H_2 gas retained in solution could be significant, leading to the difference in measured product ratios between the experiments using pre-treated cellulose and glucose solution.

Supplementary Discussion 9 | Stability of the PC sheets under outdoor operation.

Due to logistical challenges and weather conditions, it was not possible to test the large-scale system over longer durations and consecutive days. Nevertheless, longer duration and overnight experiments with the medium-scale Al:$SrTiO_3$|Co-SSP_M system under outdoor conditions using glucose as the substrate were performed. These medium-scale experiments will still be representative of the large-scale system as they were conducted under similar conditions.

A 12 h outdoor experiment was performed to demonstrate that the PC sheets can maintain their performance longer than the 6 h experiment duration of the large-scale outdoor demonstration. Considering the drop in light intensity later in the day (as well as drop in ultraviolet light intensity in the late afternoon/evening; Supplementary Fig. 55a), the PC sheets maintained good performance over the 12 h experiment (Supplementary Fig. 55b and Supplementary Table 46).

Overnight outdoor experiments were also conducted to study the performance of the PC sheets under day and night cycles. When left in the reaction solution overnight over the course of a consecutive two-day outdoor experiment (weather conditions shown in Supplementary Figs. 56a and b), the Al:$SrTiO_3$|Co-SSP_M sheet lost most of its performance on the second day (Supplementary Fig. 56c and Supplementary Table 47). This was likely due to continuous dissolution of the co-catalyst while the sheet was immersed in the reaction solution. However, if stored outside the reaction solution (i.e. in dry conditions), the PC sheet maintained its performance on the next day of operation, even after long-term storage (Supplementary Figs. 57a-c; Supplementary Table 48). Hence, proper storage conditions are important in maintaining PC sheet stability. Based on the results of these outdoor experiments, although there is still room for improvement in the stability of the PC sheets, their lifetime can be extended with suitable operating procedures.

Supplementary Discussion 10 | Effect of differences in reaction parameters on PC sheet performance when scaling up the PC sheets and experiment size.

As experimental parameters change when scaling up the Al:$SrTiO_3$|Co-SSP PC system from 1 cm^2 to 1 m^2, the potential effect of these changes should be accounted for. This is because these parameters, particularly reaction solution depth and temperature, may influence the photocatalytic performance of the PC sheets due to effects on light absorption, product gas trapping and overall reaction kinetics.[5] Hence, small- and medium-scale experiments with solution depth and temperature fixed at 1.2 cm and 40 °C, respectively, were performed. These conditions were set based on the outdoor 1 m^2 demonstration, where the large-scale photoreactor had a solution height of 1.2 cm and reached a temperature of up to ~40 °C during the outdoor experiments (Supplementary Fig. 37).

Supplementary Figure 58 shows the performance comparison of the small-scale Al:$SrTiO_3$|Co-SSP_S and medium-scale Al:$SrTiO_3$|Co-SSP_M PC sheets under standard conditions and with solution depth and temperature fixed at 1.2 cm and 40 °C, respectively. Here, standard conditions refer to a solution depth of ~1.4 and ~0.9 cm (based on reaction volume and photoreactor dimensions) for the experiments involving small- and medium-scale experiments, respectively, and room temperature. It is noted that over the course of the experiments, these experiments respectively reach a temperature of ~36 and ~45 °C due to heating from light irradiation (Supplementary Figs. 59 and 60). Nevertheless, in both the small- and medium-scale experiments, the photocatalytic performance at the different conditions was similar (Supplementary Figure 58a and b; Supplementary Table 49). This was likely because in the standard conditions, the solution depth and temperature were still close to 1.2 cm and 40 °C, respectively, as in the case of the experiments with fixed solution depth and temperature. Therefore, within the scope of this work, the photocatalytic performance of the PC sheets at

different experiment scales was not meaningfully affected by the relatively small differences in reaction parameters.

Supplementary Discussion 11 | Comparison between the Al:$SrTiO_3$|Co-SSP system and other Al:$SrTiO_3$-based photocatalytic systems.

While Al:$SrTiO_3$-based photocatalytic systems have been shown to achieve higher H_2 evolution performances than the present Al:$SrTiO_3$|Co-SSP system,[4,5] these systems are fundamentally different from the present system despite also utilising Co species as a co-catalyst. In these Al:$SrTiO_3$|$RhCr_2O_3$/CoOOH and Al:$SrTiO_3$|$RhCrO_x$/CoO_y systems (Supplementary Fig. 39), H_2 evolution still relies on a noble metal-based Rh co-catalyst, with Cr and Co species respectively functioning to inhibit backward reactions and mediate hole transfer only.[4,5] While the inclusion of a noble metal co-catalyst expectedly yields higher H_2 evolution, the scarcity, cost and environmental impact of noble metal utilisation limits the large-scale feasibility of the mentioned systems. Particularly in the case of Rh, life cycle analyses encompassing metrics related to carbon emissions, human health, terrestrial and marine toxicity, water consumption and various environmental components have also shown that Rh extraction is more negatively impactful than Pt, Pd, Au, and Ag in virtually all measured metrics.[28] Additionally, for the Al:$SrTiO_3$|$RhCrO_x$/CoO_y system,[5] the Co species are deposited by photodeposition. The practicality of photodeposition for large-scale applications is questionable as it is costly to utilise high-intensity ultraviolet light and challenging to ensure uniform light irradiation over large volumes.[29] Different from these systems, in the present Al:$SrTiO_3$|Co-SSP system, the Co species acts as the H_2 evolution co-catalyst without the need for a noble metal and is deposited by high-throughput spray coating, thus enhancing its feasibility for large-scale application.

Another key difference between the present Al:$SrTiO_3$|Co-SSP system and reported systems is the type of reaction the systems are applied for. In this work, the reactions of interest are various solid waste reforming reactions (H_2 evolution coupled to the oxidation of organic substrates) while the mentioned Al:$SrTiO_3$-based systems were only used for overall water splitting (H_2 evolution coupled to water oxidation). While being more thermodynamically favourable than water oxidation, the use of organic substrates can introduce new challenges in the photocatalytic process.

Compared to water oxidation, the oxidation of organic substrates is more complex and proceeds via a multi-step mechanism with the formation of various intermediates. It is possible that these intermediates can adsorb strongly onto and deactivate the catalyst.[30,31] Even for the benchmark Al:$SrTiO_3$ light absorber with $RhCrO_x$ co-catalyst system used in this study (which utilises the same Rh H_2 evolution co-catalyst as the Al:$SrTiO_3$ systems in Supplementary Figure 39), H_2 evolution rates when paired with organic substrate oxidation were far below those paired with water oxidation (Supplementary Fig. 43). This result is supported by previous literature utilising the same PC system for organic substrate and water oxidation.[32] Hence, for these Al:$SrTiO_3$-based systems, organics oxidation is significantly less favourable than water oxidation, which further accounts for the seemingly poorer photocatalytic performance of the Al:$SrTiO_3$|Co-SSP system compared to those in literature. However, it is noted that the production of value-added chemicals rather than O_2 remains an advantage of photocatalytic reforming compared to overall water splitting.

Additionally, in the case of the Al:$SrTiO_3$|Co-SSP system, the H_2 evolution rates when coupled even to real-world waste-derived organic substrate oxidation was significantly higher than when coupled to water oxidation (Fig. 3d), suggesting that the Al:$SrTiO_3$|Co-SSP system is more suitable for photocatalytic reforming than the benchmark Al:$SrTiO_3$|$RhCrO_x$ system. This gives some indication on the advantages of this system in reforming waste to value-added organics compared to previous reports utilising Al:$SrTiO_3$.

Supplementary Discussion 12 | Technoeconomic and sensitivity analysis of 1 m^2 photocatalytic system.

In addition to its photocatalytic performance, the economic aspects of the large-scale Al:$SrTiO_3$|Co-SSP_L system was also evaluated via a technoeconomic analysis (TEA). To obtain an accurate economic assessment, the scope of this analysis was limited to the actual operation and performance of the 1 m^2 photoreactor. This means that the production cost of H_2 was calculated based on the volume of H_2 produced and the associated capital and operational costs of the conducted experiments. The TEA of the photocatalytic system has been conducted on an actual large-scale demonstration with measured experimental data. Energy consumption for PC synthesis (heating of catalyst precursors) was also measured with an energy meter (Supplementary Fig. 61). As the data used in the TEA were measured from real experiments, the only necessary assumption was on photoreactor lifetime (note that the photoreactor lifetime refers only to the device in which the photocatalytic reaction takes place, and not the stability of the PC panels themselves). Thus, a conservative assumption of 100 days was made for photoreactor lifetime to account for wear and tear during panel and solution loading and unloading.

The large-scale demonstrations were performed with freshly prepared Al:$SrTiO_3$|Co-SSP_L in pre-treated cellulose and glucose, as well as Al:$SrTiO_3$|Co-SSP_L with reused Al:$SrTiO_3$ and re-deposited Co-SSP in glucose (total of three runs). These experiments were then used as the three scenarios considered in the TEA. In the case of glucose reforming with Al:$SrTiO_3$ reuse, the amount of H_2 produced and its associated capital and operating costs were taken from the cumulative data of both experiments using glucose as a substrate.

From the TEA (Fig. 5e and Supplementary Tables 30−33), the cheapest H_2 cost of £0.93 mmol^{-1} was obtained from the use of glucose as a substrate with photocatalyst reuse. This was expected as the relative contribution of all materials to the operating cost, other than the Co-SSP, decreases with increasing number of experiment runs. This means that the H_2 cost could potentially be significantly reduced by extended reuse of the Al:$SrTiO_3$ panels. It is also noted that the calculated H_2 cost also does not factor in the value of valorised oxidation products which will further improve the economic aspect of the photocatalytic reforming system. On the other hand, the most expensive H_2 cost of £7.44 mmol^{-1} was obtained from the use of pre-treated cellulose as a substrate due to the reduced H_2 production rate from the poisoned PC. It should be emphasised, however, that a TEA considers only economic aspects, while the use of pre-treated cellulose or other waste-derived substrates has additional advantages in terms of waste treatment and environmental remediation. Other accompanying analyses such as a life cycle analysis could give a more holistic view of the applicability of the Al:$SrTiO_3$|Co-SSP_L system, particularly for waste-derived substrate reforming.

For the Al:SrTiO$_3$|Co-SSP$_L$ system, the calculated cost of H_2 under all scenarios was drastically higher than those available in literature.[2,33–35] The reason for this difference becomes clear when considering the assumptions made in previous TEA. As these TEA were based on hypothetical systems (Supplementary Fig. 39, grey region; Supplementary Table 29), various parameters, particularly those relating to photocatalytic activity and stability, were assumed to be 1−2 orders of magnitude higher than any reported system. It is therefore expected that the resulting H_2 cost estimation would be vastly underestimated. While such results may appear more attractive, data based on a real large-scale system would be more appropriate to guide research on scaled up photocatalytic systems.

Following the technoeconomic analysis, a sensitivity analysis was also conducted to determine the most significant factors affecting the cost of H_2 from the Al:SrTiO$_3$|Co-SSP$_L$ photocatalytic system. The factors considered were photocatalyst reuse, light intensity, daylight hours, reactor lifetime and substrate source. The sensitivity analysis was performed based on "pessimistic", "base" and "optimistic" cases for each factor which respectively represent unfavourable conditions, actual experimental conditions and favourable conditions. Data for the "base" case were taken from the large-scale demonstration using glucose as a substrate with photocatalyst reuse.

The sensitivity analysis (Fig. 5f and Supplementary Tables 34 and 35) shows that H_2 cost is most influenced by light intensity as sunlight is the main driving force for H_2 evolution. Hence, selecting locations with high solar insolation and implementing solar tracking will be critical in producing competitively-priced H_2 from photocatalytic systems. Solar concentration can also be used to increase light intensity, but the temperature dependence of photocatalytic reactions must be considered. Another major factor affecting H_2 cost is PC reuse. A large portion of operating costs stem from PC (and co-catalyst) materials. Thus, PC with good long-term stability will be required for producing cheap H_2. Currently, while PC systems with solar-to-hydrogen efficiencies of up to 5% have been reported, the stabilities of these highly efficient PC remain in the time scale of hours or even minutes, with few notable exceptions.[36–38] Research with an emphasis on PC stability is therefore of great importance. Other than that, the sensitivity analysis suggests that substrate source could markedly affect H_2 cost in certain cases. When enzymatic pre-treated cellulose was taken as the substrate, the cost of H_2 was over 4× higher than when glucose was used. This was due to the poor performance of Al:SrTiO$_3$|Co-SSP$_L$ in pre-treated cellulose. However, the use of waste-derived substrates would be preferable for photocatalytic reforming systems as H_2 evolution can be coupled to waste valorisation. Therefore, it would be advantageous to select a more suitable waste and/or pre-treatment procedure. Aside from enzymatic treatment, other treatment methods such as alkaline hydrolysis, acid hydrolysis or hydrothermal treatment should be explored to supply the photocatalytic system with a suitable feedstock which does not inhibit catalysis.[39–41]

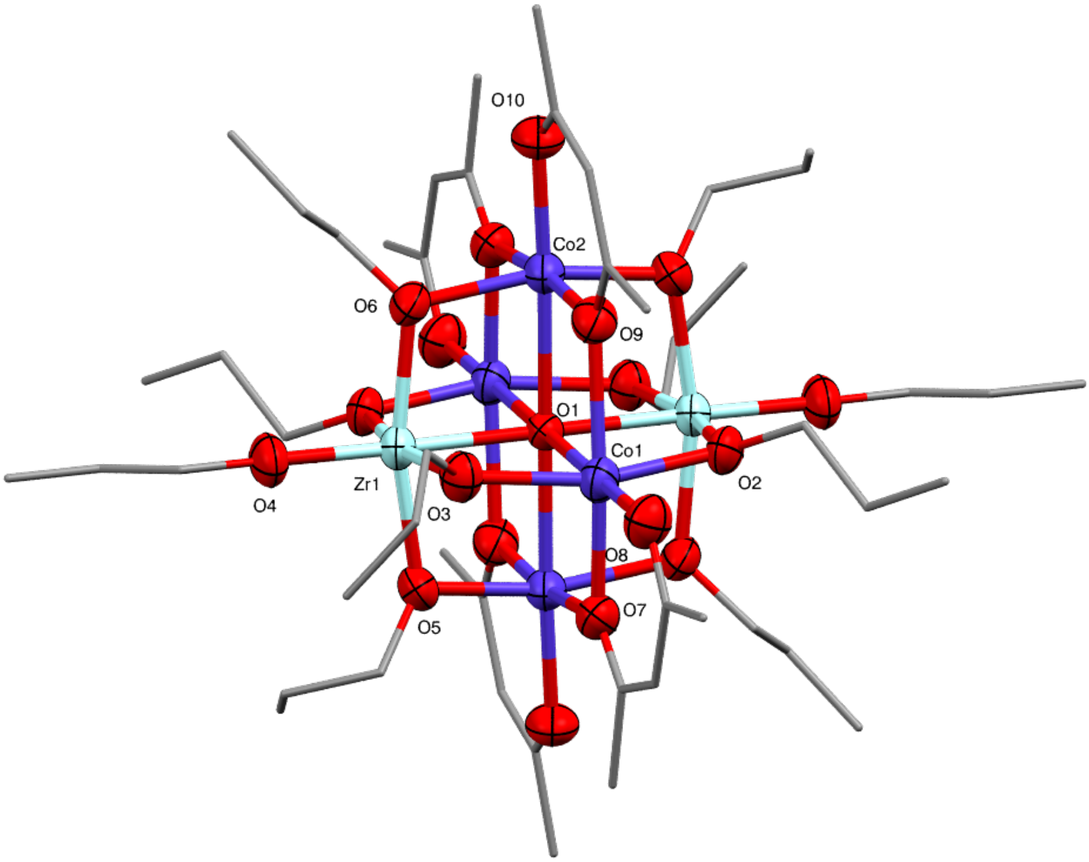

Supplementary Figure 1 | Molecular structure of $Co_4Zr_2O(O^nPr)_{10}(acac)_4$ (ellipsoids at 50% probability) with H atoms and minor disorder of some *n*−propoxide ligands omitted for clarity. Selected bond lengths (Å) and angles (°): Co1–O1 2.3583(7), Co1–O2 2.009(3), Co1–O3 2.008(3), Co1–O7 2.066(3), Co1–O8 2.023(4), Co1–O9 2.307(3), Co2–O1 2.3643(7), Co2–O5 2.003(3), Co2–O6 1.996(3), Co2–O7 2.314(4), Co2–O9 2.066(4), Co2–O10 2.022(4), Zr1–O1 2.2123(5), Zr1–O2 2.086(3), Zr1–O3 2.106(3), Zr1–O4 1.932(3), Zr1–O5 2.089(3), Zr1–O6 2.098(3), O–Co1–O 81.71(10)−98.44(15), O–Co2–O 81.58(9)−100.82(14), O–Zr1–O 83.28(9)−97.44(14).

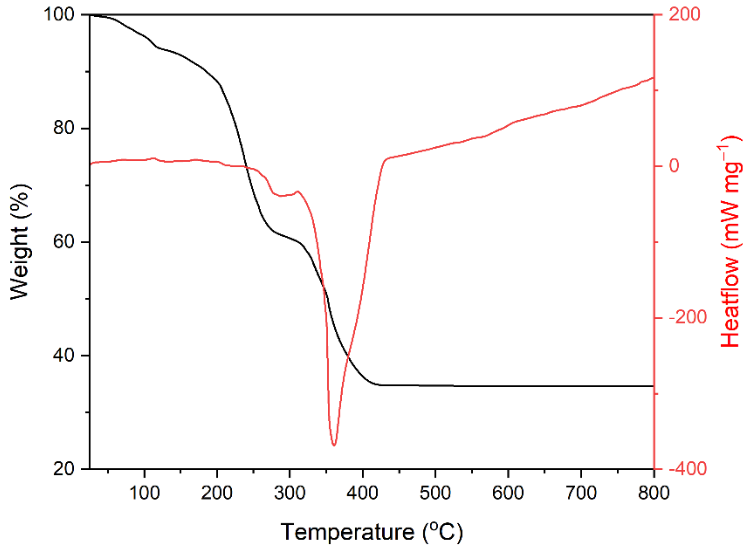

Supplementary Figure 2 | Thermogravimetric analysis (TGA) of Co-Zr alkoxide crystals heated from 25−800 °C at 10 °C min^{-1} in air.

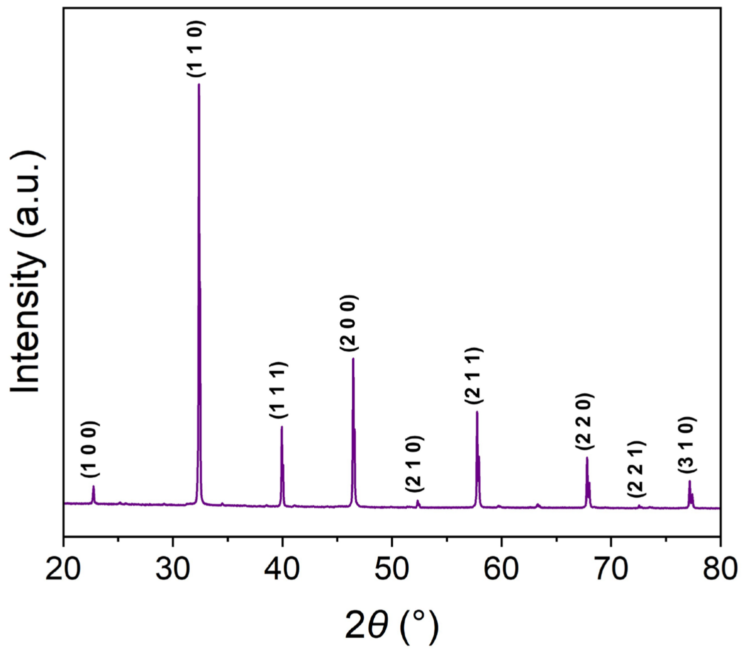

Supplementary Figure 3 | Full view of X-ray diffraction (XRD) patterns for Al:$SrTiO_3$|Co-SSPs. The most intense reflections in the powder pattern match those of $SrTiO_3$ (ICDD 00-035-0734).

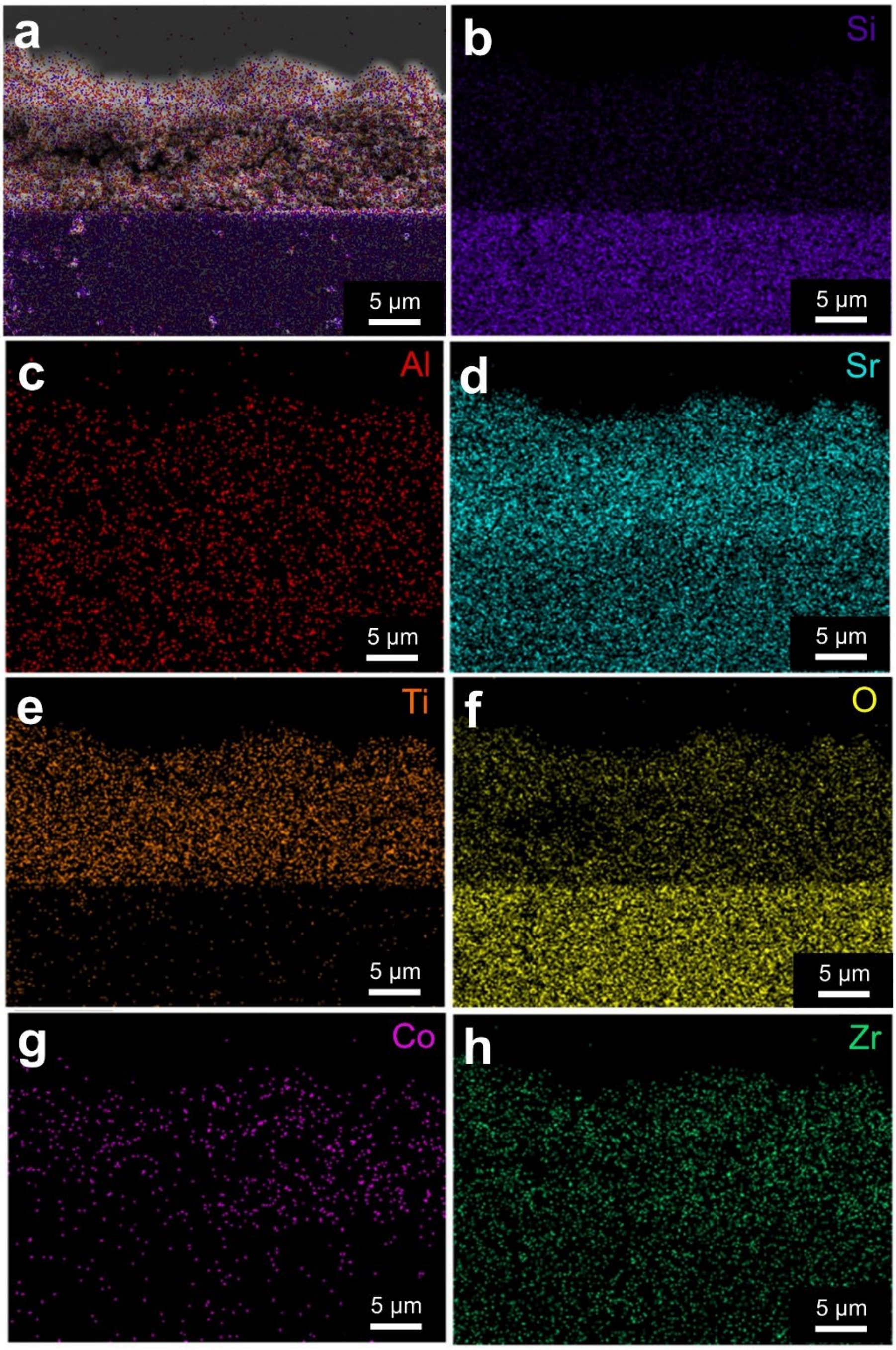

Supplementary Figure 4 | Cross-section SEM-EDX elemental mapping of Al:$SrTiO_3$|Co-SSPs. a-h, Overlay (**a**), silicon (**b**), aluminium (**c**), strontium (**d**), titanium (**e**), oxygen (**f**), cobalt (**g**) and zirconium (**h**) elemental mapping.

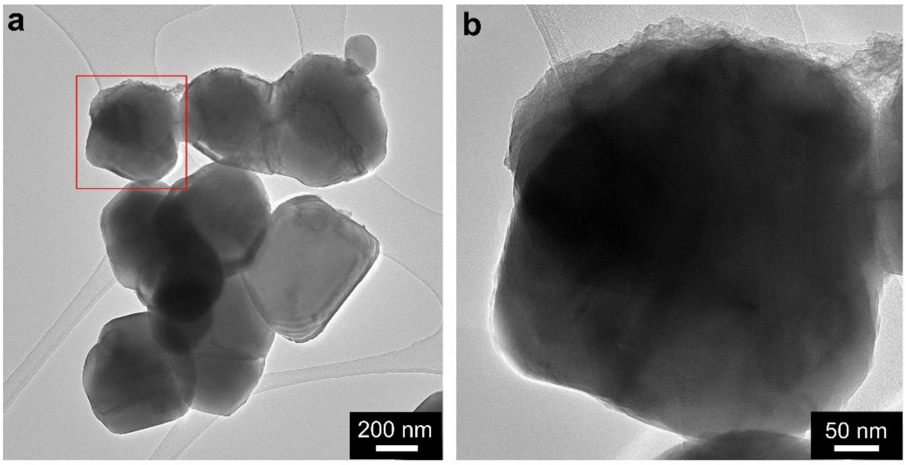

Supplementary Figure 5 | TEM image of Al:SrTiO3|Co-SSP. a, Low magnification TEM image of Al:SrTiO3|Co-SSP powder. **b,** Magnified view of the region bounded by the red square.

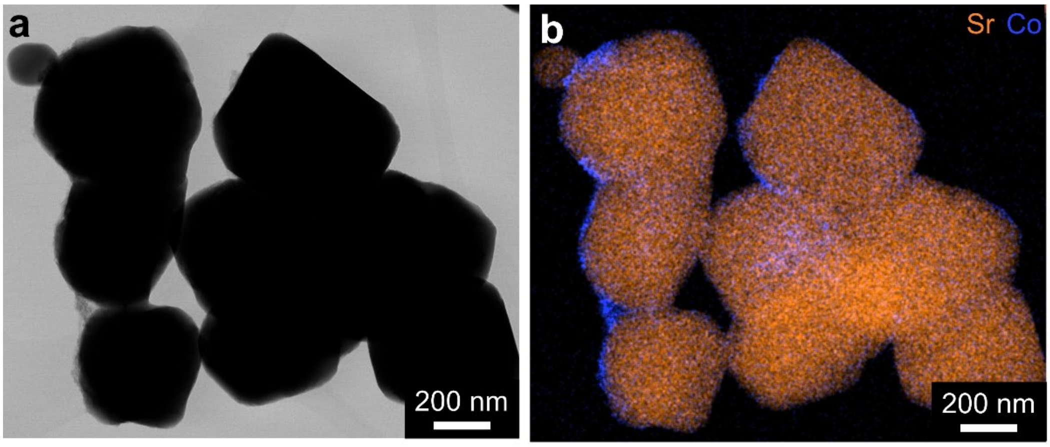

Supplementary Figure 6 | BF-STEM image and EDX elemental mapping of Al:SrTiO3|Co-SSP. a, Bright-field STEM image of Al:SrTiO3|Co-SSP powder. **b,** Corresponding Sr and Co overlay EDX elemental mapping.

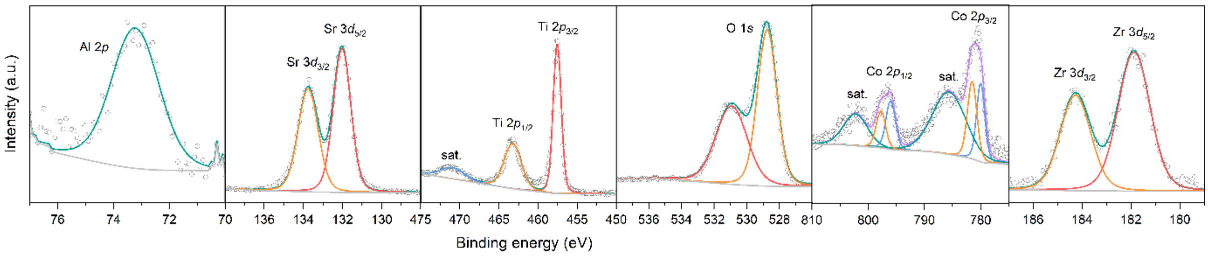

Supplementary Figure 7 | Curve-fitted spectra XPS spectra of as-prepared Al:SrTiO3|Co-SSPs. The spectra of Al for the post-catalytic samples are shown without fitting as no noticeable peaks were detected. The lack of noticeable peaks is likely due to the relatively small amount of Al present due to low doping concentration. Sat., satellite peak.

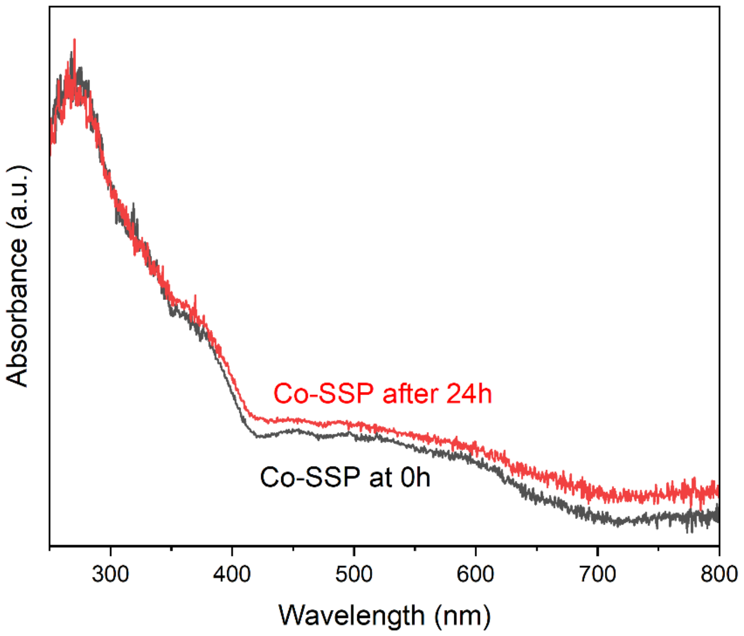

Supplementary Figure 8 | UV−visible diffuse reflectance spectra of Co-SSP at 0 h and 24 h after precursor deposition. The Co-SSP was kept under ambient conditions during the 24 h storage.

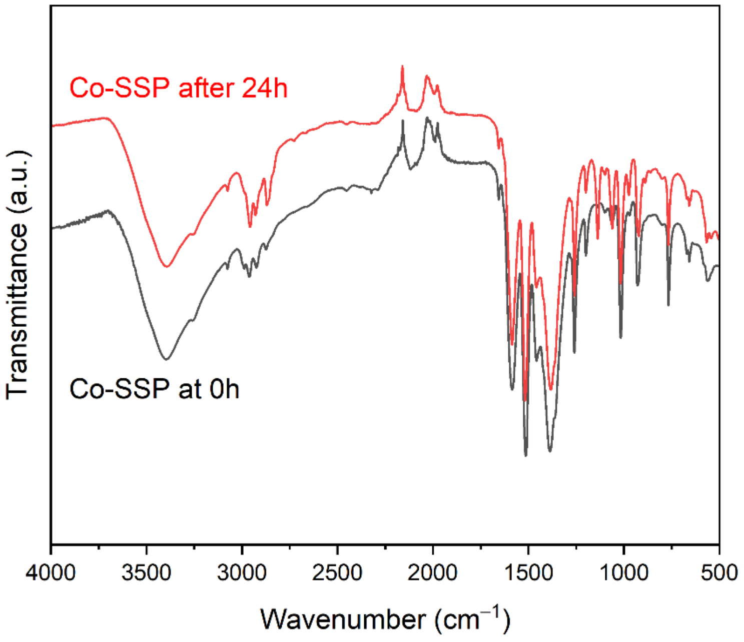

Supplementary Figure 9 | FTIR spectra of Co-SSP at 0 h and 24 h after precursor deposition. The Co-SSP was kept under ambient conditions during the 24 h storage. The FTIR spectrum indicates that some organic ligand is still present, particularly $Co(acac)_2$, which shows peaks at 1597 cm^{-1} for ν(C=O) and 1525 cm^{-1} for ν(C=C).

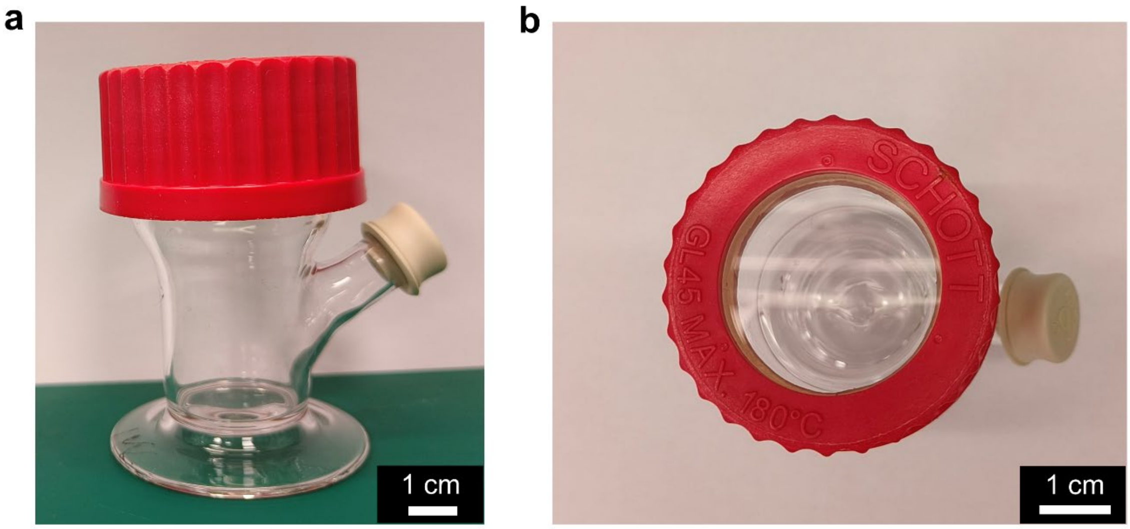

Supplementary Figure 10 | Photographs of top-irradiation-type glass photoreactor used for small-scale (1 cm^2) photocatalytic reactions. Side-view (**a**) and top-view (**b**) photographs of the photoreactor.

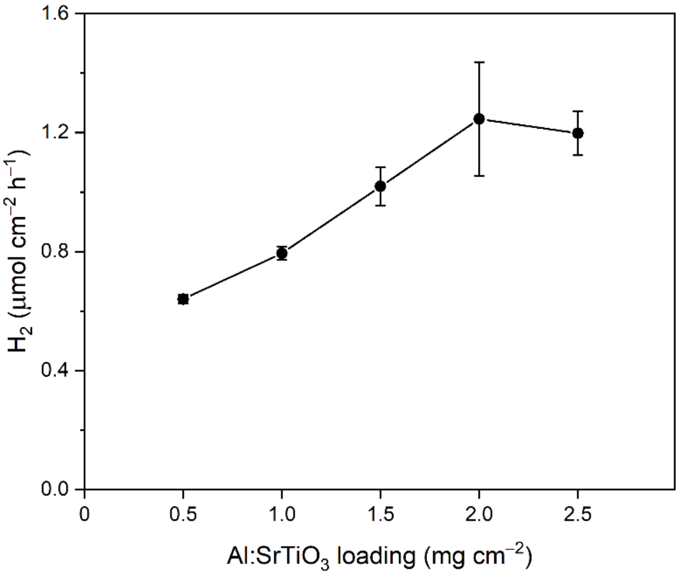

Supplementary Figure 11 | Optimisation of Al:$SrTiO_3$ loading on Al:$SrTiO_3$|Co-SSPs. 30 µl of 0.04 M Co-SSP solution was spin coated onto Al:$SrTiO_3$. The photocatalytic experiments were performed with TEOA under AM1.5G illumination for 22 h at room temperature. The data are presented as mean values ± SD for reactions performed in triplicate (n = 3).

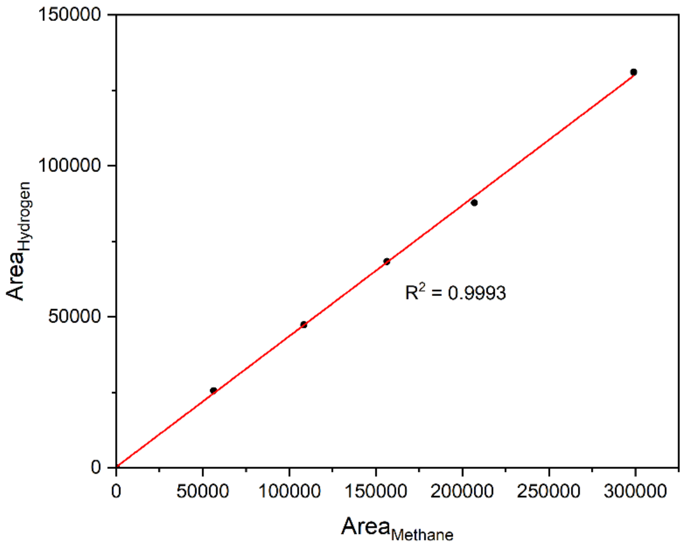

Supplementary Figure 12 | Gas chromatography calibration curve for H_2. 2% CH_4 was used as an internal standard.

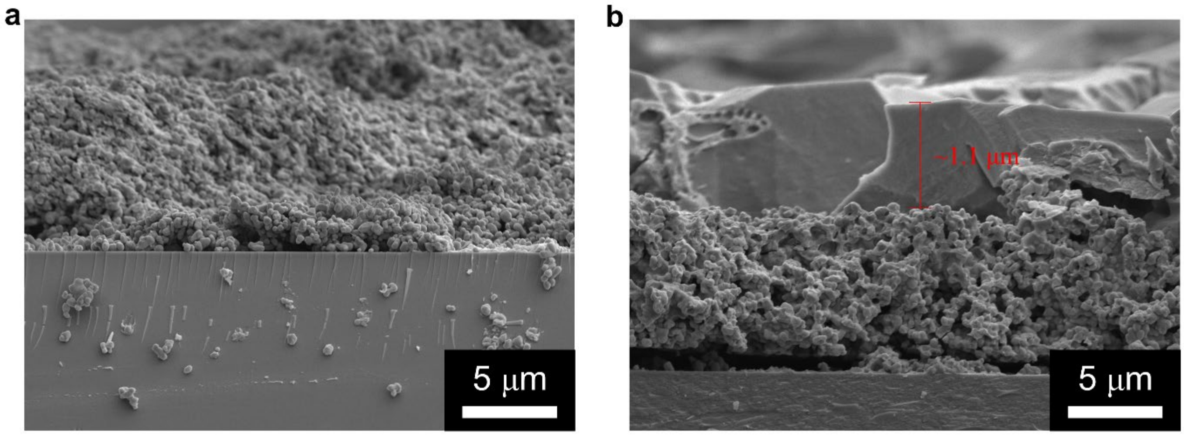

Supplementary Figure 13 | Cross-sectional SEM images of Al:SrTiO$_3$|Co-SSP PC sheets with different Co-SSP deposition methods. a,b, Images of the PC sheets with Co-SSP deposited using spin coating (**a**) and drop casting (**b**). The Co-Zr species were well-dispersed on the Al:SrTiO$_3$ when Co-SSP was deposited using spin coating and no discernible co-catalyst layer was formed, while the Co-SSP deposited using drop casting had a co-catalyst layer thickness of ~1.1 μm.

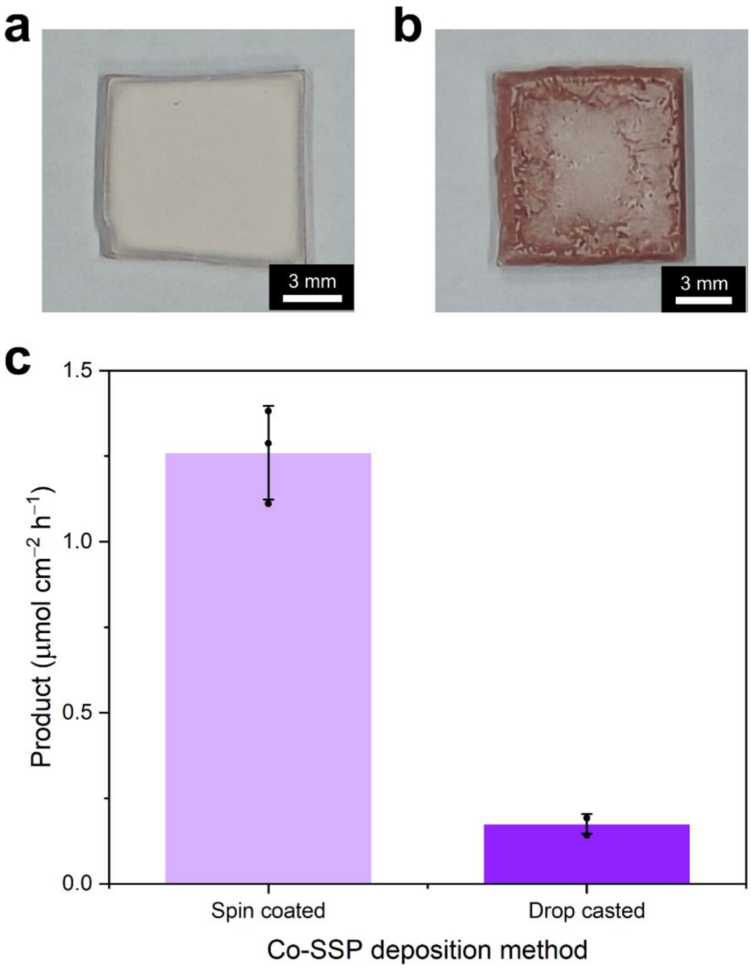

Supplementary Figure 14 | Comparison between Al:SrTiO$_3$ PC sheets with Co-SSP deposited via different methods. a,b, Photographs of PC sheets with spin coated (**a**) and drop casted (**b**) Co-SSP. **c,** H_2 evolution comparison between Al:SrTiO$_3$ PC sheets with spin coated and drop casted Co-SSP. The photocatalytic experiments were performed under AM1.5G illumination for 22 h at room temperature. The data in (**c**) are presented as mean values ± SD for reactions performed in triplicate (n = 3).

photocatalytic experiments were performed under AM1.5G illumination for 22 h at room temperature. The data are presented as mean values ± SD for reactions performed in triplicate (n = 3).

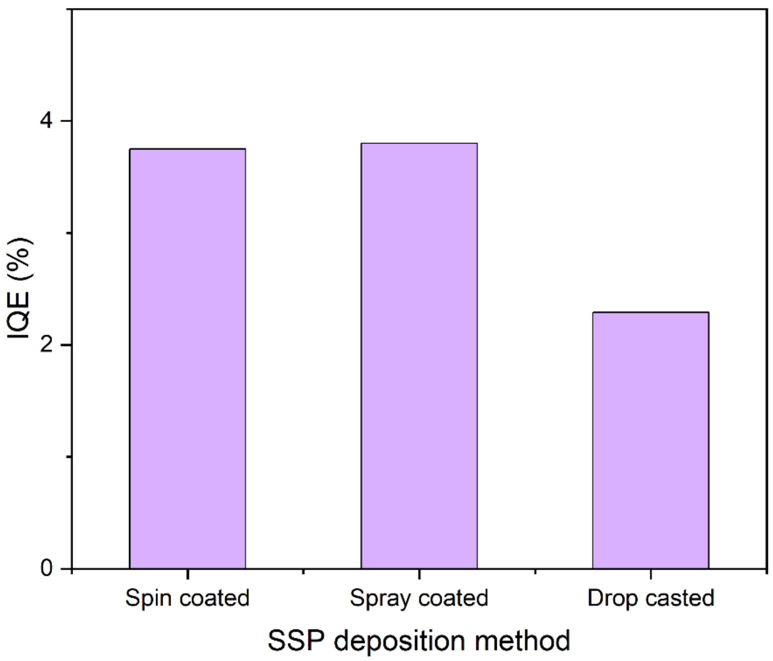

Supplementary Figure 15 | Internal quantum efficiency (IQE) of the PC sheets at 350 nm with different Co-SSP deposition methods. Glucose was used as the substrate.

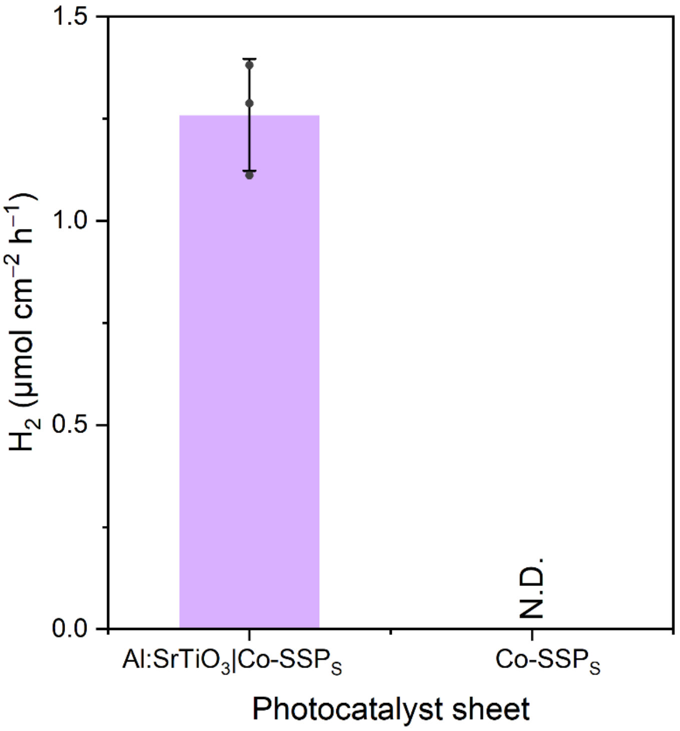

Supplementary Figure 16 | H_2 evolution of Al:$SrTiO_3$|Co-SSP_S and Co-SSP_S only. The photocatalytic experiments were performed under AM1.5G illumination for 22 h at room temperature. The data are presented as mean values ± SD for reactions performed in triplicate (n = 3).

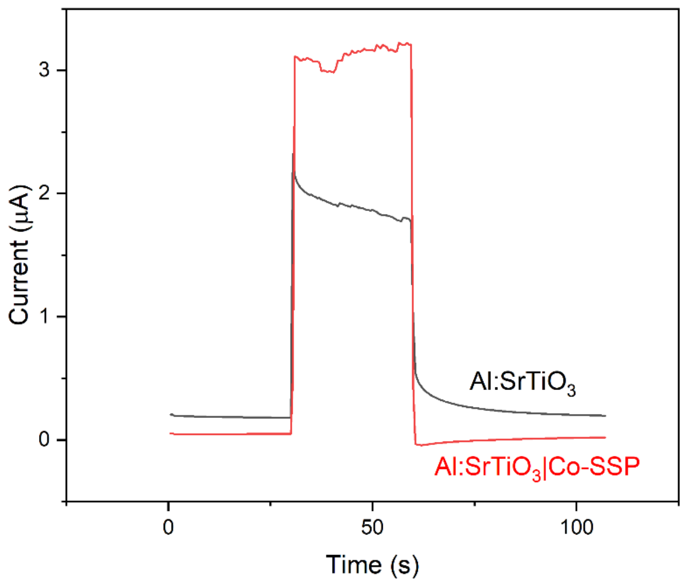

Supplementary Figure 17 | Chronoamperometry traces of Al:$SrTiO_3$ and Al:$SrTiO_3$|Co-SSP photoelectrodes. Measurements were performed in 12 ml 0.1 M TEOA under AM1.5G illumination.

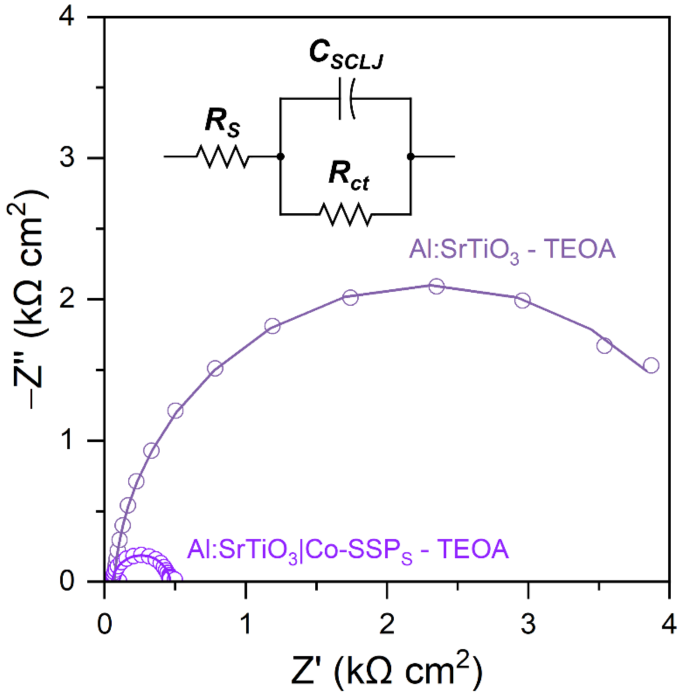

Supplementary Figure 18 | Nyquist plots of the PEIS response (open circles) recorded with frequency ranges from 1 MHz to 0.5 Hz and a 15-mV sinusoidal AC perturbation amplitude at –0.4 V vs RHE as well as the corresponding fitting curves (solid lines). Inset: proposed Randles equivalent circuit, C_{SCLJ} was replaced by a constant phase element (CPE) during fitting to account for the non-ideal capacitive behaviours of the photoelectrodes. Measurements were performed in 30 ml stirred electrolyte containing 0.1 M Na_2SO_4 and 0.1 M TEOA under AM1.5G illumination.

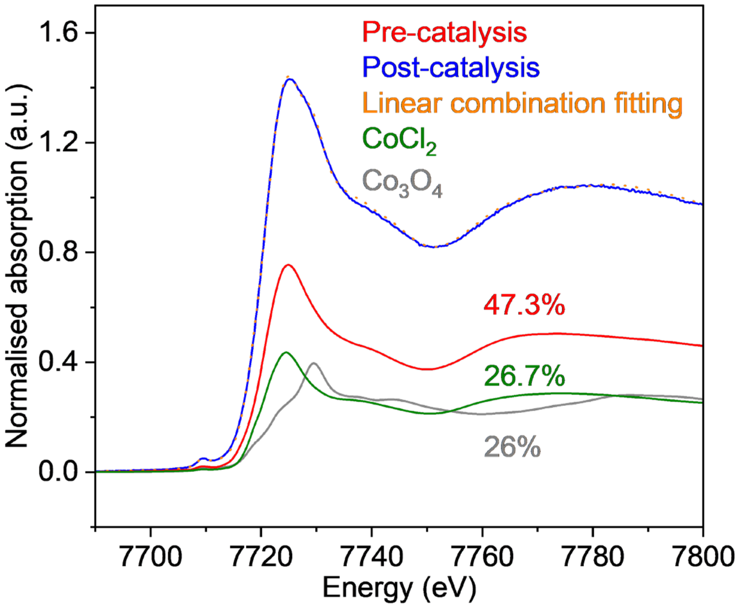

Supplementary Figure 19 | Linear combination fitting of the post-catalysis XANES spectrum using reference profiles from the pre-catalysis sample (47.3%), $CoCl_2$ (26.7%), and Co_3O_4 (26%).

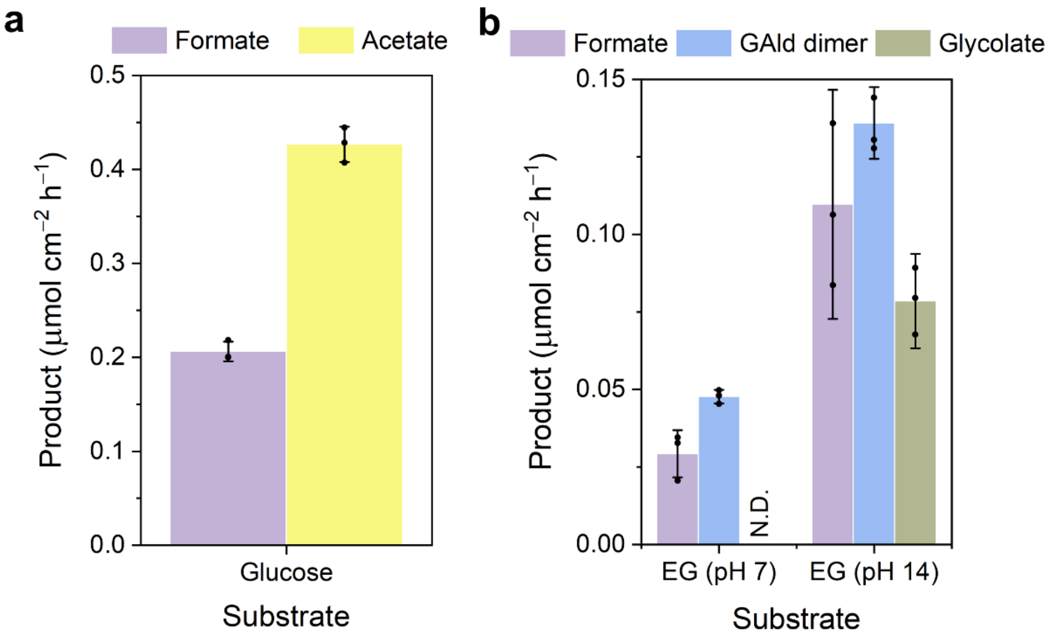

Supplementary Figure 20 | Oxidation products of Al:$SrTiO_3$|Co-SSPs using model substrates. a,b, Oxidation products using glucose (**a**) and EG (**b**) as substrate. The photocatalytic experiments were performed under AM1.5G illumination for 22 h at room temperature. The data are presented as mean values ± SD for reactions performed in triplicate (n = 3).

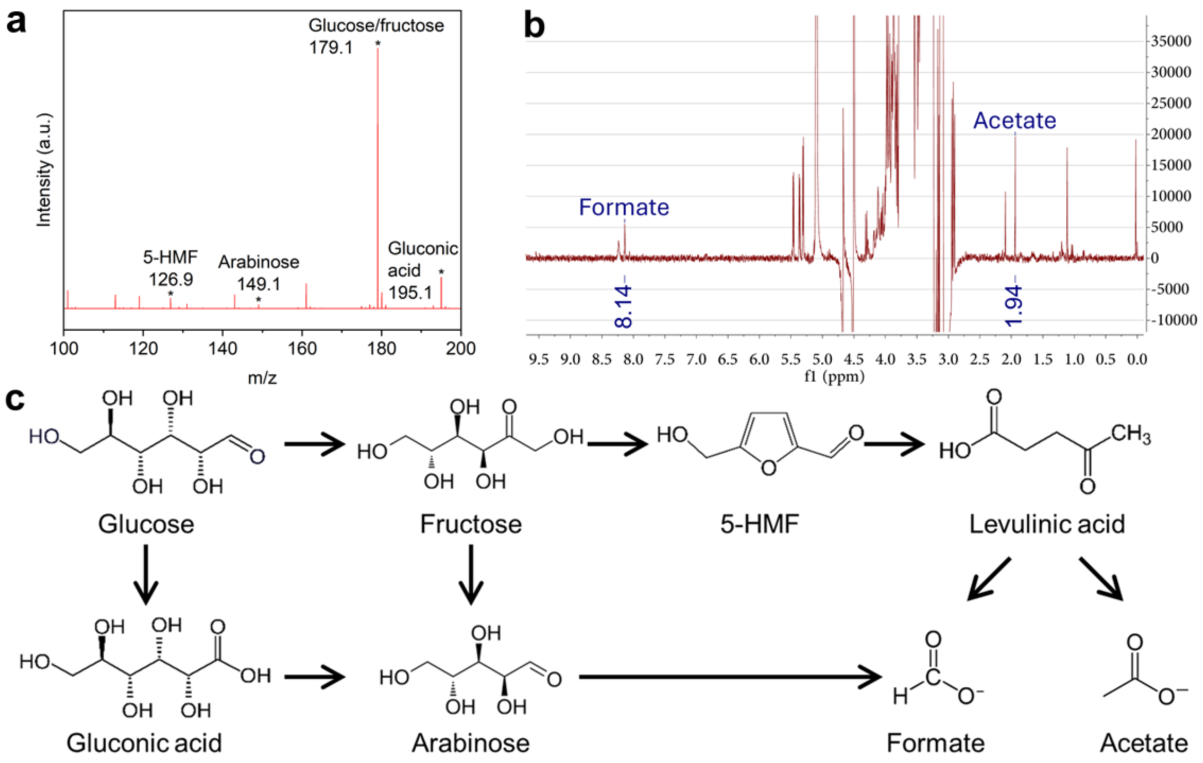

Supplementary Figure 21 | Reaction mechanism for glucose oxidation. a,b, The mass spectrum (**a**) and the ^{1}H NMR spectrum (**b**) of the post-photocatalysis reaction mixture. **c,** Proposed reaction scheme for glucose oxidation.

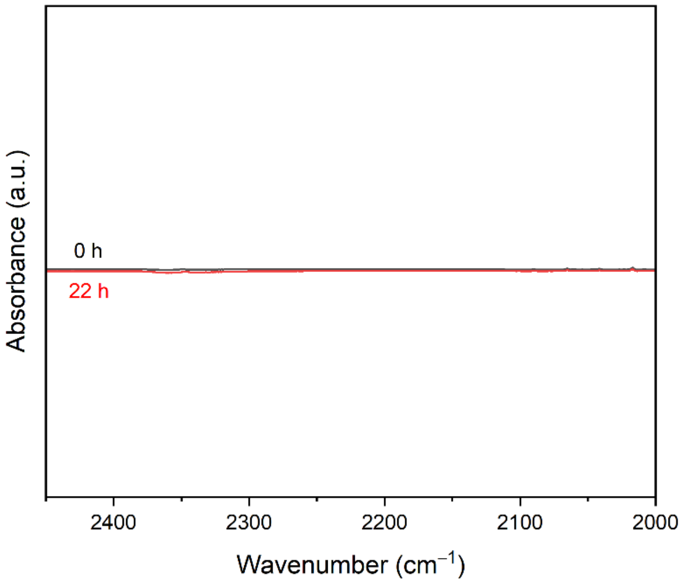

Supplementary Figure 22 | FTIR spectra of headspace gas before and after glucose photoreforming experiment using Al:SrTiO$_3$|Co-SSPs PC sheet. Absorption peaks of CO_2 are expected to be at ≈2350 cm^{-1}, respectively. After withdrawing the headspace gas for the 0 h measurement, the reactor was re-purged with N_2 (with 2% CH_4 as internal standard) and the experiment was performed as normal. The photocatalytic experiment was performed under AM1.5G illumination for 22 h at room temperature.

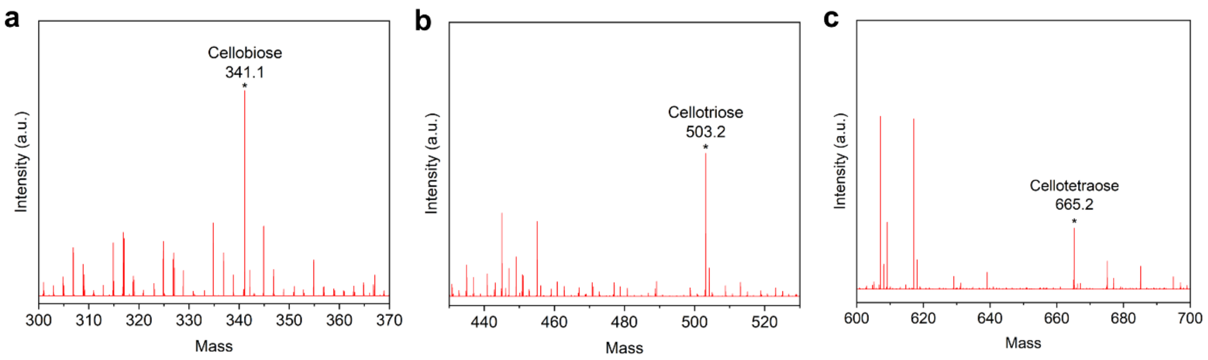

Supplementary Figure 23 | Liquid chromatography–mass spectrometry analysis of pre-treated cellulose solution. a-c, Spectral region showing the peaks of cellobiose (**a**), cellotriose (**b**) and cellotetraose (**c**).

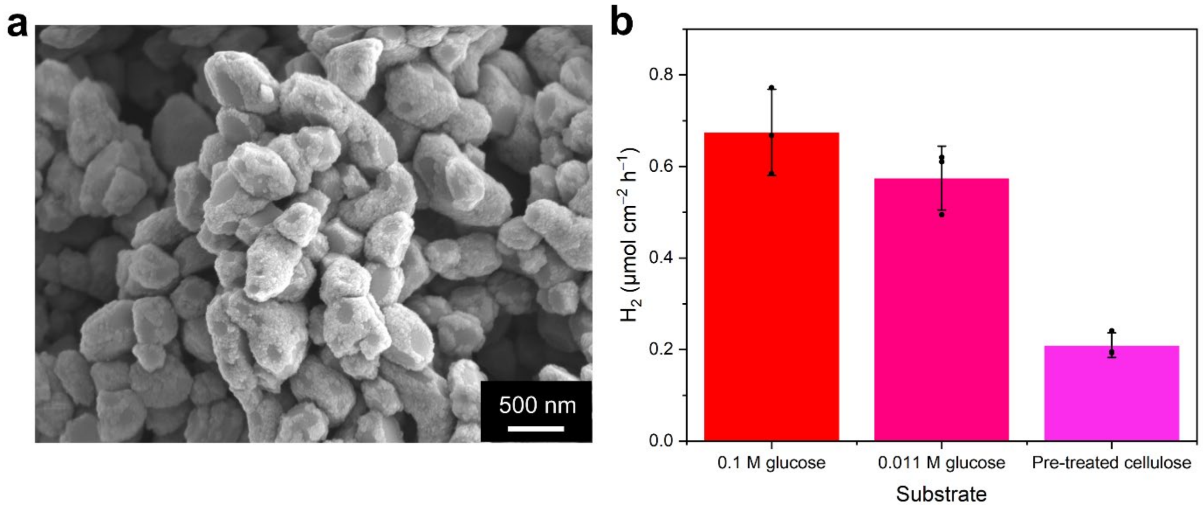

Supplementary Figure 24 | Photocatalyst deactivation by pre-treated cellulose substrate. a, Top-view SEM images showing the attachment of intermediates from cellulose pre-treatment which causes deactivation of the Al:$SrTiO_3$ photocatalyst. **b,** Comparison of H_2 evolution of Al:$SrTiO_3$|Co-SSP_S using high and low concentrations of glucose, as well as pre-treated cellulose as substrate. 0.011 M glucose was chosen as the low-substrate-concentration condition as the pre-treatment of cellulose yielded the same concentration of glucose. The photocatalytic experiments were performed under AM1.5G illumination for 22 h at room temperature. The data in (**b**) are presented as mean values ± SD for reactions performed in triplicate (n = 3).

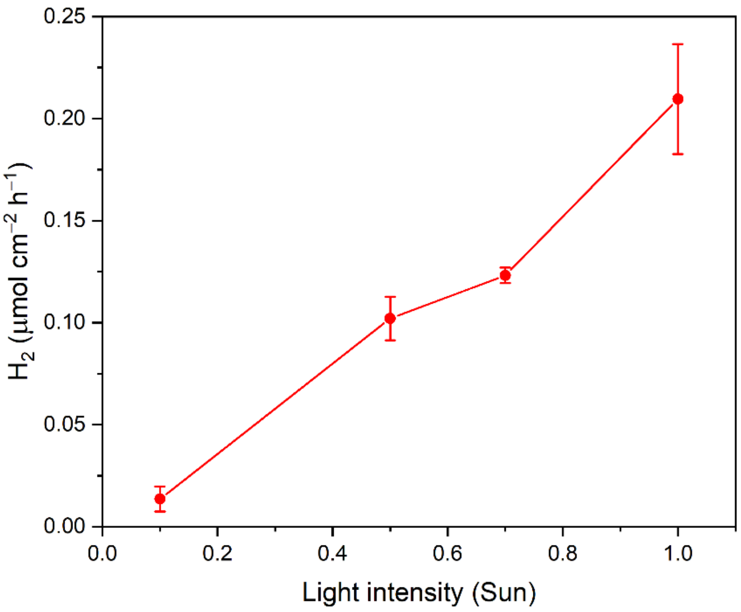

Supplementary Figure 25 | H_2 evolution of Al:SrTiO$_3$|Co-SSPs under various light intensities using pre-treated cellulose as the substrate. The photocatalytic experiments were performed for 22 h at room temperature. The data are presented as mean values ± SD for reactions performed in triplicate (n = 3).

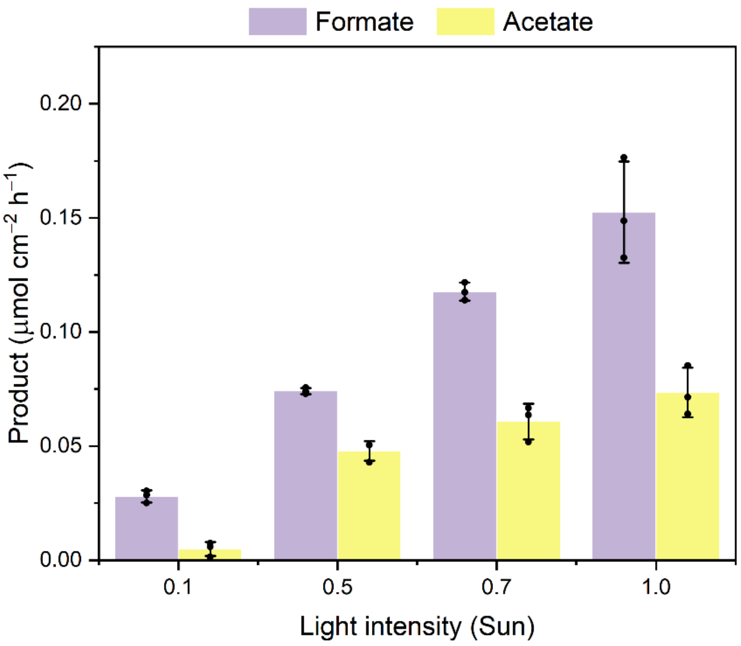

Supplementary Figure 26 | Oxidation products of Al:SrTiO$_3$|Co-SSPs under various light intensities using pre-treated cellulose as the substrate. The photocatalytic experiments were performed for 22 h at room temperature. The data are presented as mean values ± SD for reactions performed in triplicate (n = 3).

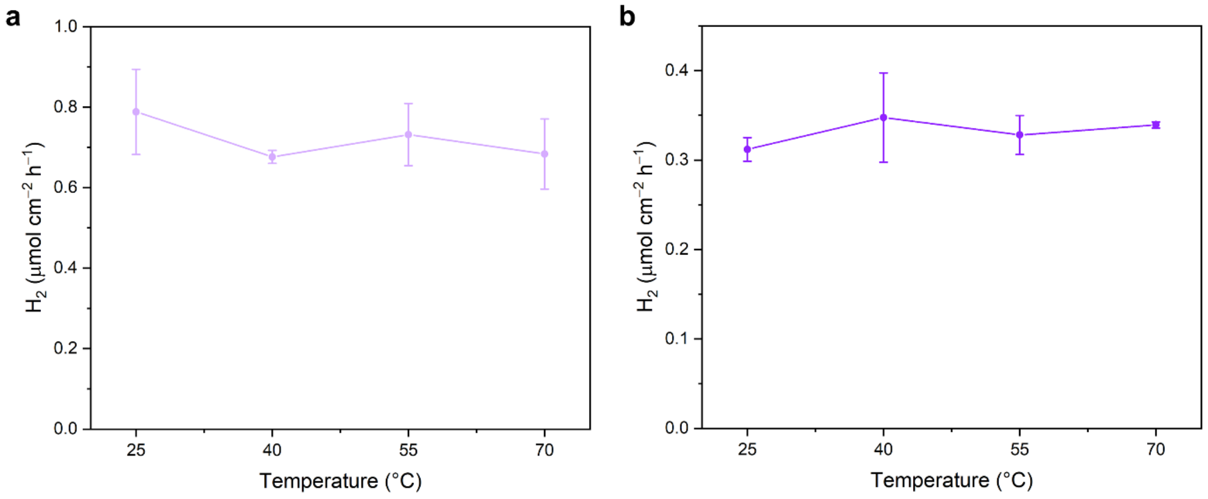

Supplementary Figure 27 | Performance of PC sheets at different temperatures. a,b, Performance of Al:$SrTiO_3$|Co-SSP_S (**a**) and Al:$SrTiO_3$|Co-SSP_M (**b**) from 25−70 °C. The photocatalytic experiments were performed in 0.1 M glucose solution under AM1.5G illumination for 6 h with the solution depth fixed at 1.2 cm. The data are presented as mean values ± SD for reactions performed in triplicate (n = 3).

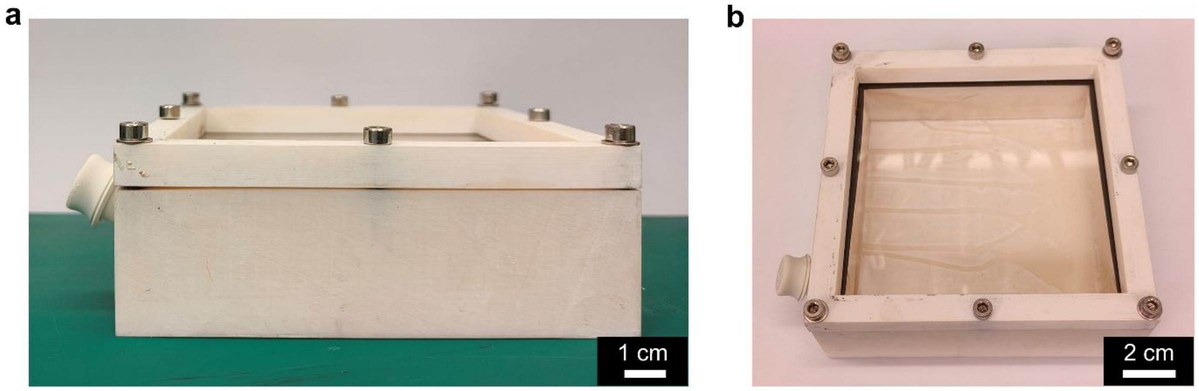

Supplementary Figure 28 | Photographs of 3D-printed top-irradiation-type photoreactor used for medium-scale (20.25 cm^2) photocatalytic reactions. Side-view (**a**) and top-view (**b**) photographs of the photoreactor.

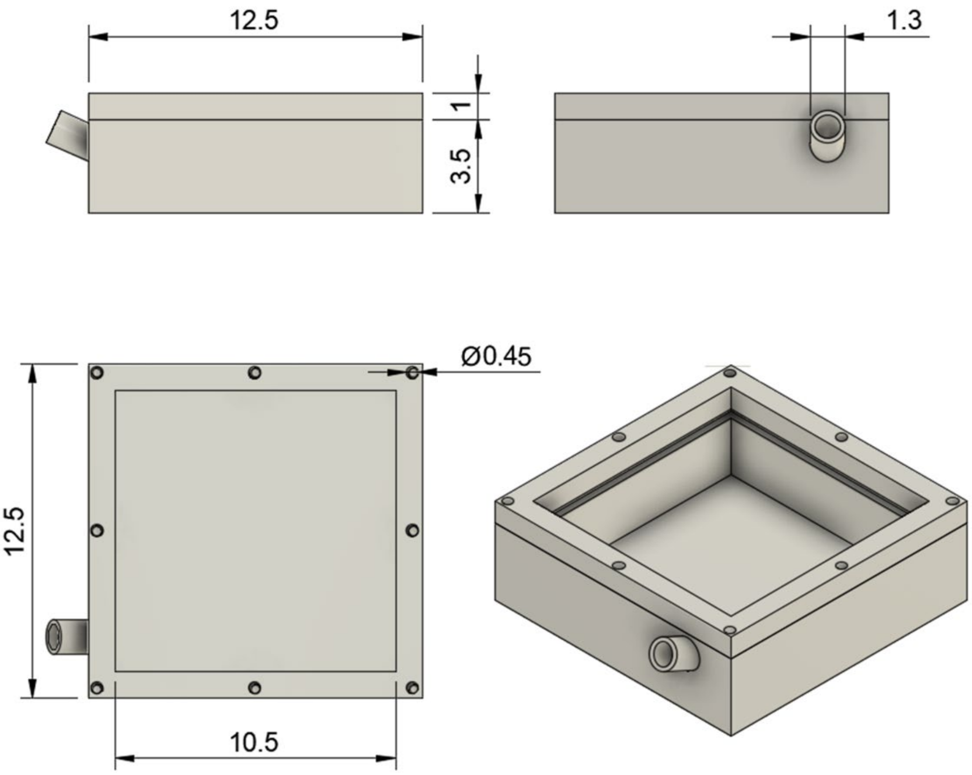

Supplementary Figure 29 | Schematic diagram and dimensions of the 3D-printed photoreactor used for medium-scale (20.25 cm^2) photocatalytic reactions. The schematic shows side, front, top and isometric views of the photoreactor. Dimensions are listed in cm.

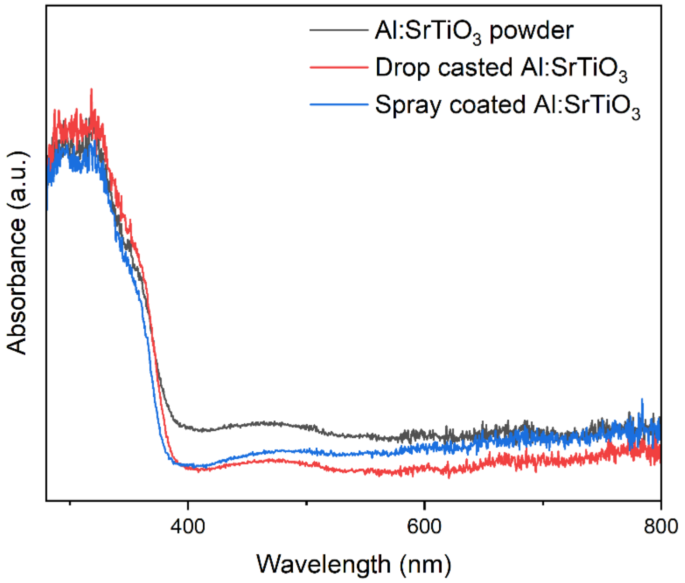

Supplementary Figure 30 | UV−visible diffuse reflectance spectra of Al:SrTiO$_3$ powder, drop casted Al:SrTiO$_3$ and spray coated Al:SrTiO$_3$.

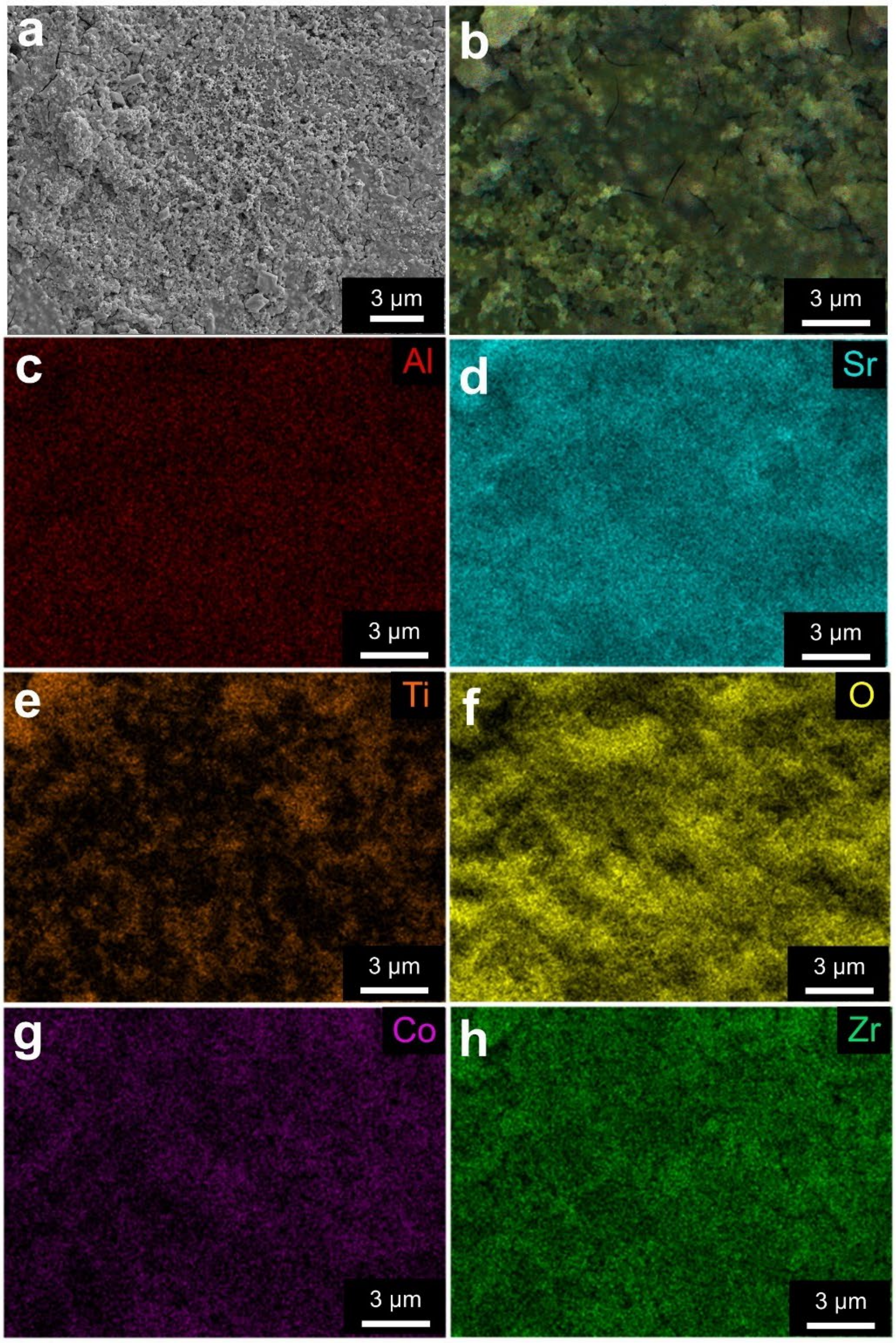

Supplementary Figure 31 | Top-view SEM images and EDX elemental mapping of Al:SrTiO$_3$|Co-SSP$_M$. a, Top-view SEM image. **b-h,** Overlay (**b**), aluminium (**c**), strontium (**d**), titanium (**e**), oxygen (**f**), cobalt (**g**) and zirconium (**h**) elemental mapping.

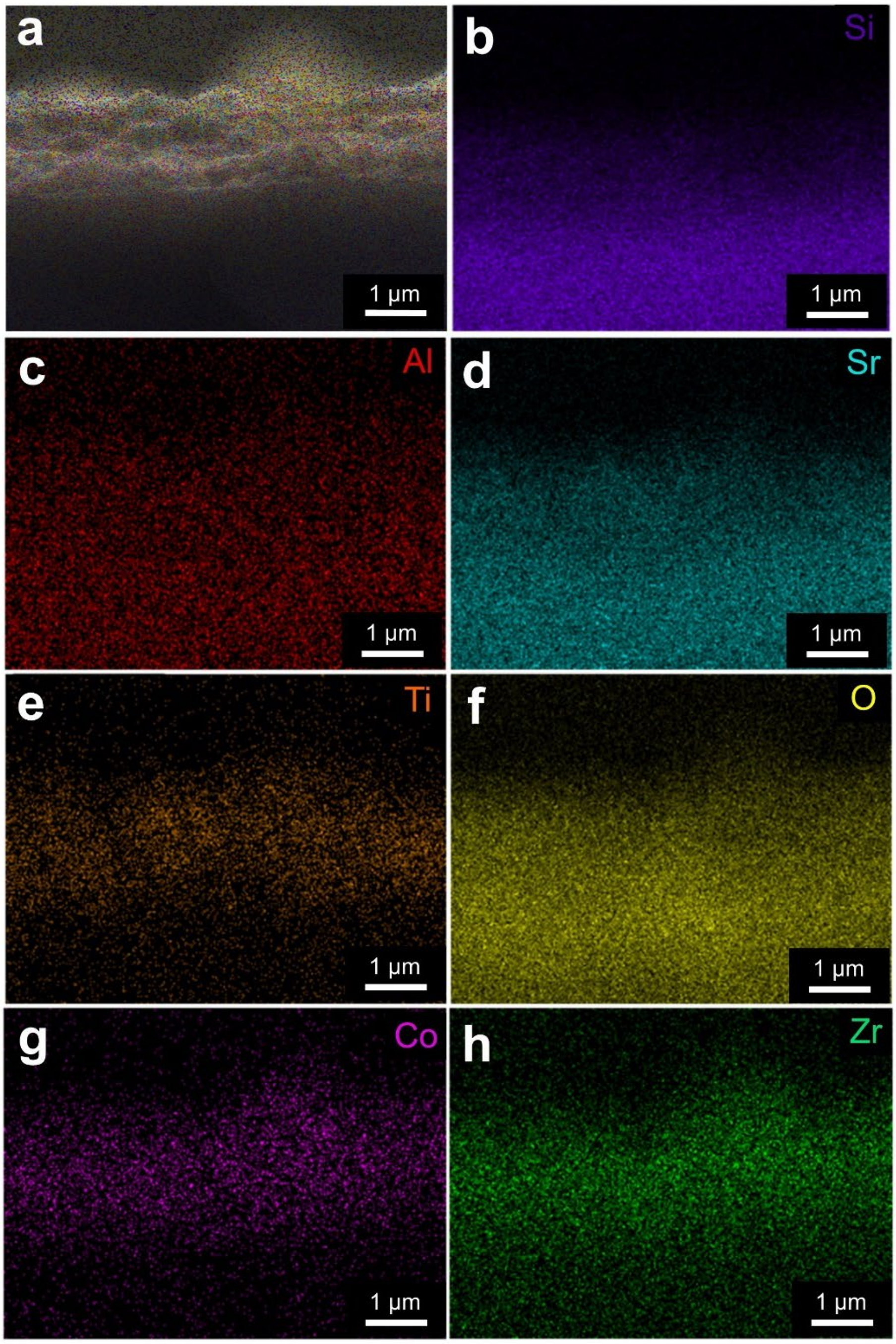

Supplementary Figure 32 | Cross-section SEM-EDX elemental mapping of Al:$SrTiO_3$|Co-SSP_M. a-h, Overlay (**a**), silicon (**b**), aluminium (**c**), strontium (**d**), titanium (**e**), oxygen (**f**), cobalt (**g**) and zirconium (**h**) elemental mapping.

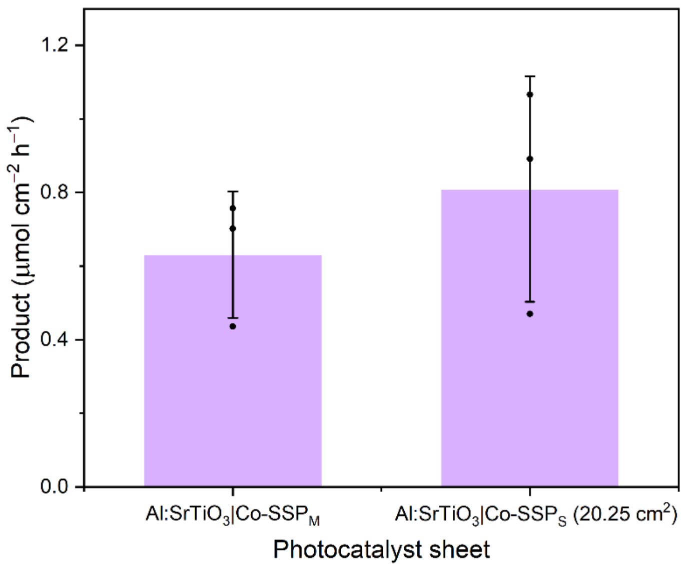

Supplementary Figure 33 | Performance comparison between Al:$SrTiO_3$|Co-SSP_M and 20.25 cm^2 Al:$SrTiO_3$|Co-SSP_S PC sheets. The photocatalytic experiments were performed under AM1.5G illumination for 6 h. The data are presented as mean values ± SD for reactions performed in triplicate (n = 3).

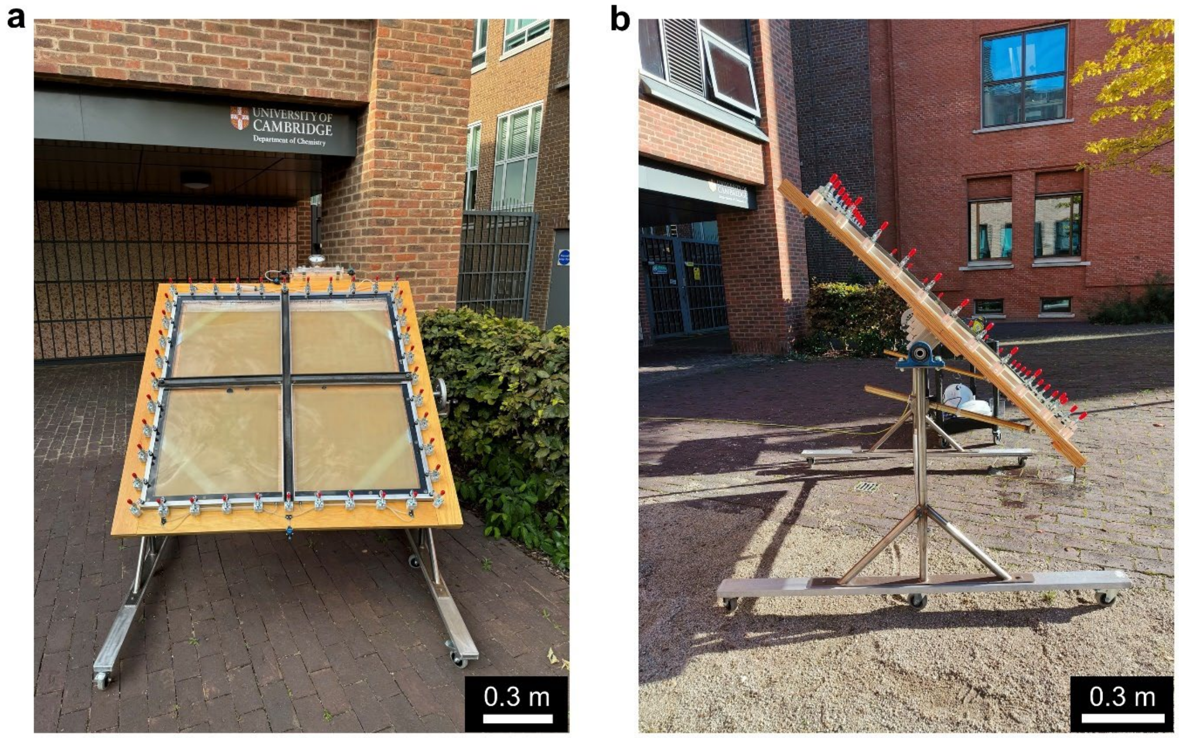

Supplementary Figure 34 | Photographs of large-scale panel photoreactor during outdoor demonstration of Al:$SrTiO_3$|Co-SSP_L PC sheets under natural sunlight. a,b, Front-view (**a**) and side-view (**b**) photograph of photoreactor.

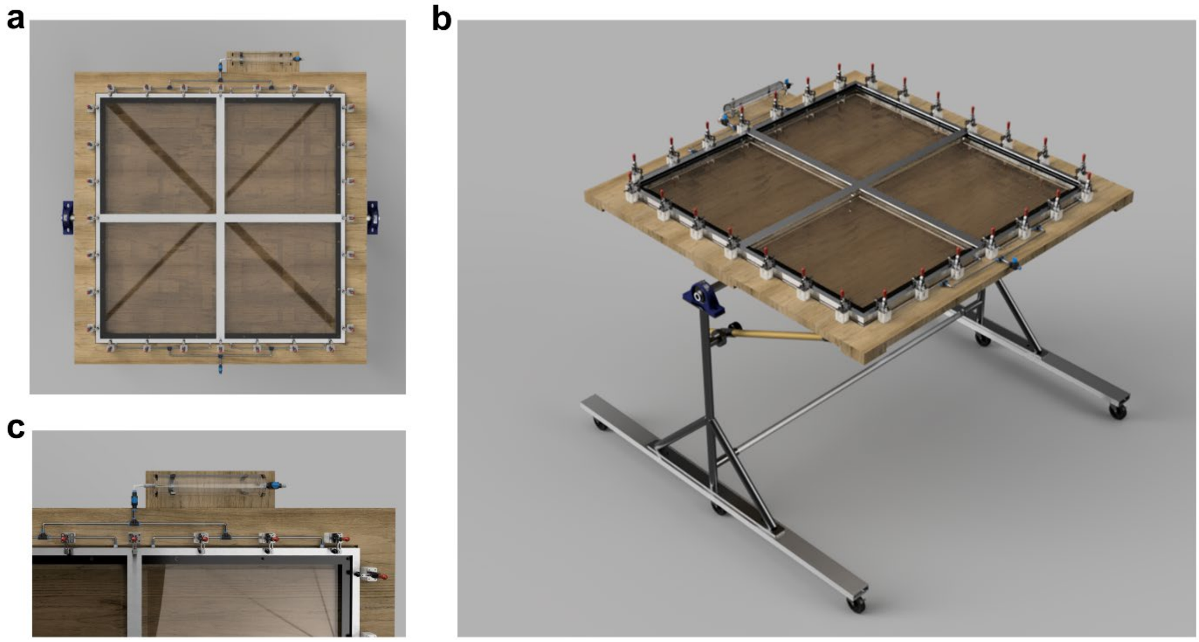

Supplementary Figure 35 | 3D renders of the large-scale panel photoreactor used for outdoor demonstration of Al:SrTiO$_3$|Co-SSP$_L$ PC sheets. a,b, Top-view (**a**) and side-view (**b**) of the panel photoreactor. **c,** Gas sample collection chamber for the panel photoreactor.

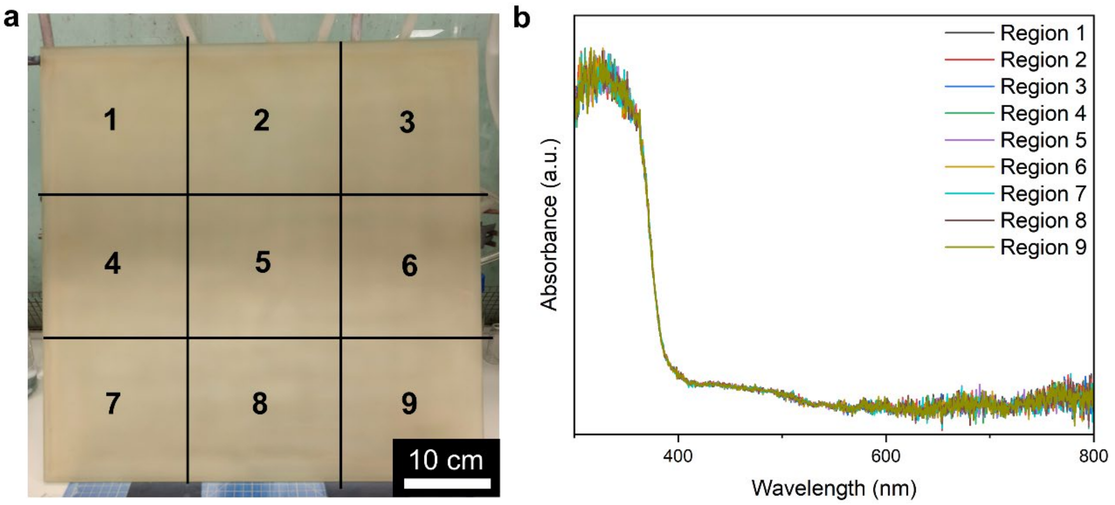

Supplementary Figure 36 | Light absorption across the Al:SrTiO$_3$|Co-SSP$_L$ PC sheet. a, Photograph of as-prepared Al:SrTiO$_3$|Co-SSP$_L$ PC sheet. The different regions in which UV−visible diffuse reflectance spectra were recorded is marked. **b,** UV−visible diffuse reflectance spectra of each region of the Al:SrTiO$_3$|Co-SSP$_L$ PC sheet.

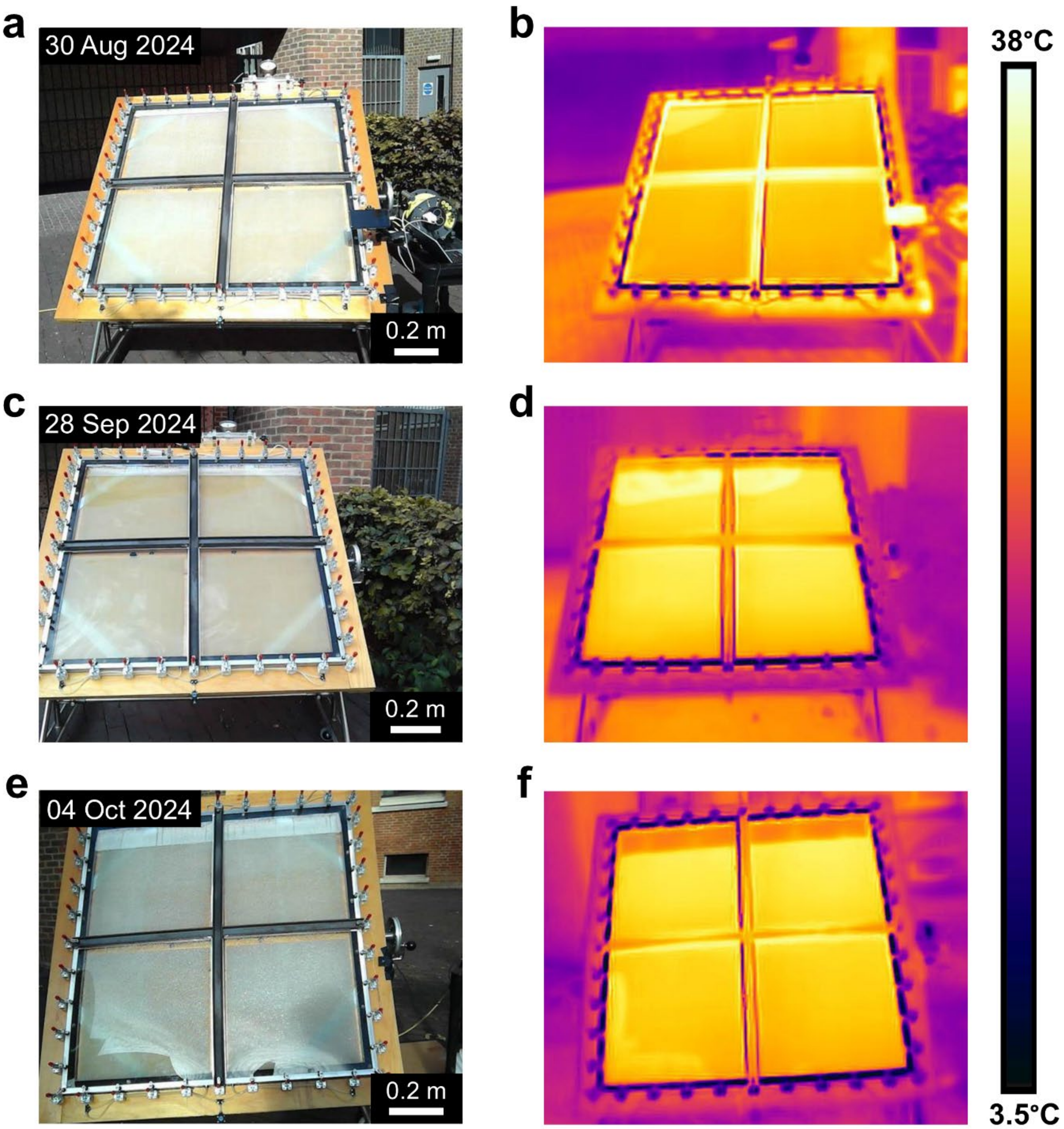

Supplementary Figure 37 | Thermal images of large-scale panel photoreactor during outdoor demonstration of Al:$SrTiO_3$|Co-SSP_L PC sheets under natural sunlight. a,b, Digital (**a**) and thermal (**b**) image of the photoreactor during the experiment on 30 August 2024. **c,d,** Digital (**c**) and thermal (**d**) image of the photoreactor during the experiment on 28 September 2024. **e,f,** Digital (**e**) and thermal (**f**) image of the photoreactor during the experiment on 4 October 2024. A temperature scale bar is included for the thermal images.

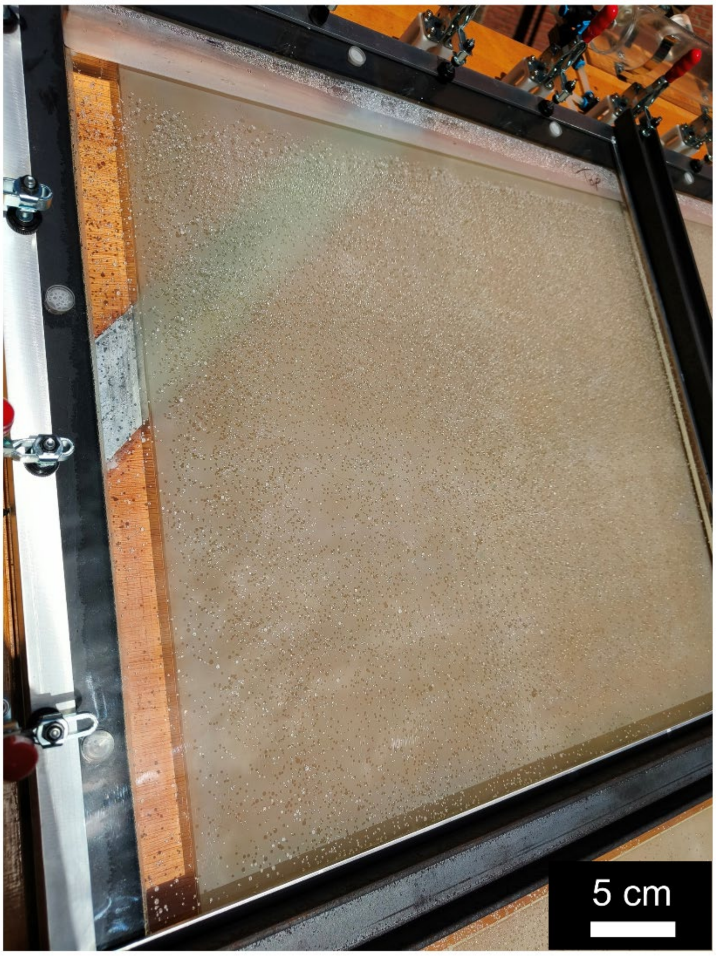

Supplementary Figure 38 | Photograph showing gas bubble formation during outdoor demonstration of Al:$SrTiO_3$|Co-SSP_L PC sheets under natural sunlight. A video of gas bubble formation can be found in Supplementary Video 2.

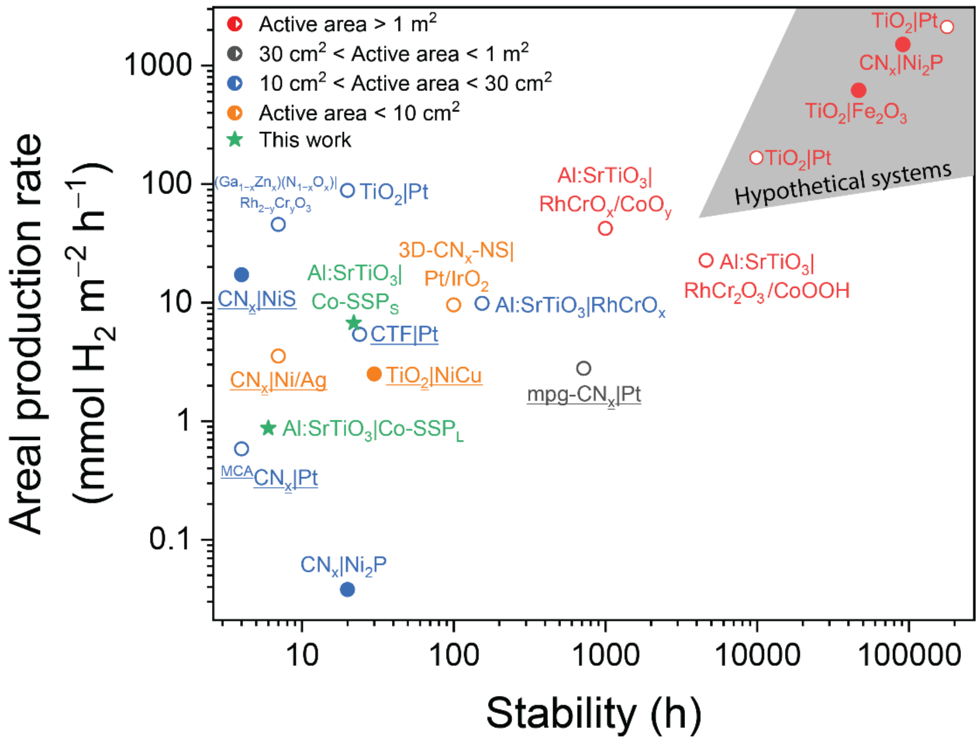

Supplementary Figure 39 | Literature summary of real and hypothetical photocatalytic systems. Hollow and solid symbols indicate photocatalytic systems with and without noble metals, respectively. Underlined systems utilise valuable sacrificial electron donors (e.g., TEOA, ethanol, etc.) as substrate. Values used for the Al:$SrTiO_3$|Co-SSP system were with glucose as the substrate.

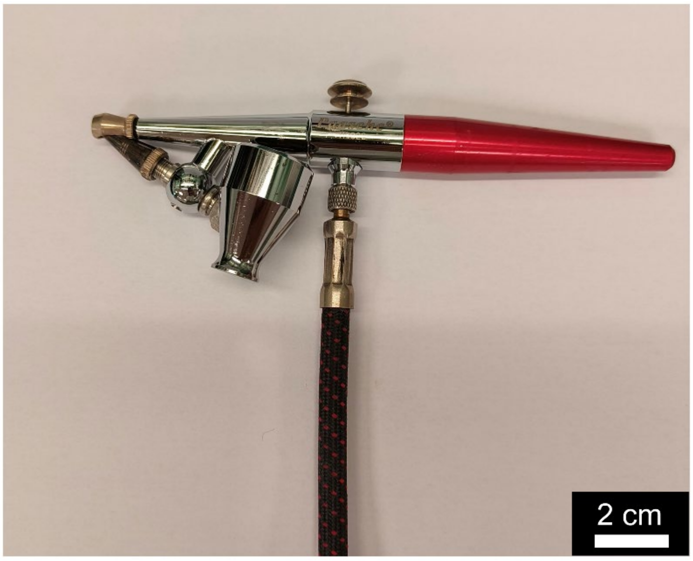

Supplementary Figure 40 | Photograph of the airbrush used for spray coating catalyst onto medium- and large-scale PC sheets. The catalyst dispersion is loaded into a reservoir and the airbrush (Paasche H Series Single Action Suction Feed Airbrush) uses a flow of N_2 to disperse the catalyst.

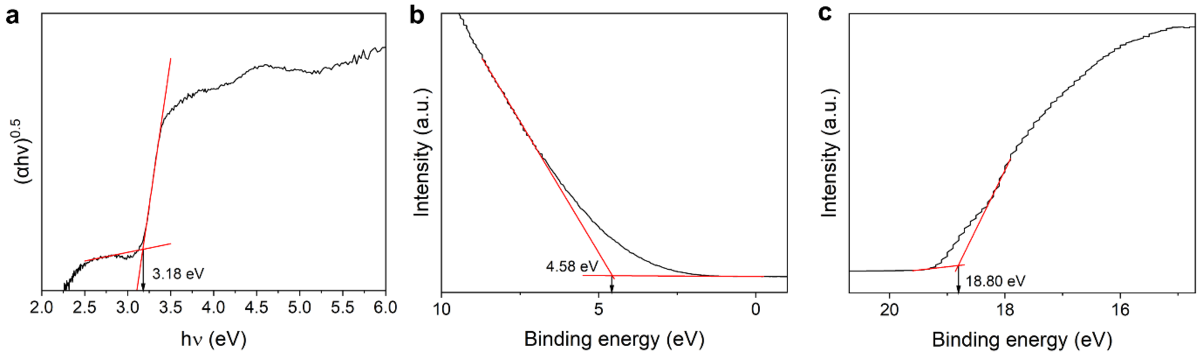

Supplementary Figure 41 | Band structure determination of Al:SrTiO$_3$. a, Tauc plot derived from the UV-visible spectrum of Al:SrTiO$_3$. **b,c,** Ultraviolet photoelectron spectroscopy spectra (He−Iα=21.22 eV) corresponding to valence band and cutoff regions of Al:SrTiO$_3$. The valence band maximum and conduction band minimum of Al:SrTiO$_3$ were measured to be +2.15 V (vs the reversible hydrogen electrode (RHE), pH 7) and −1.03 V (vs RHE, pH 7), respectively.

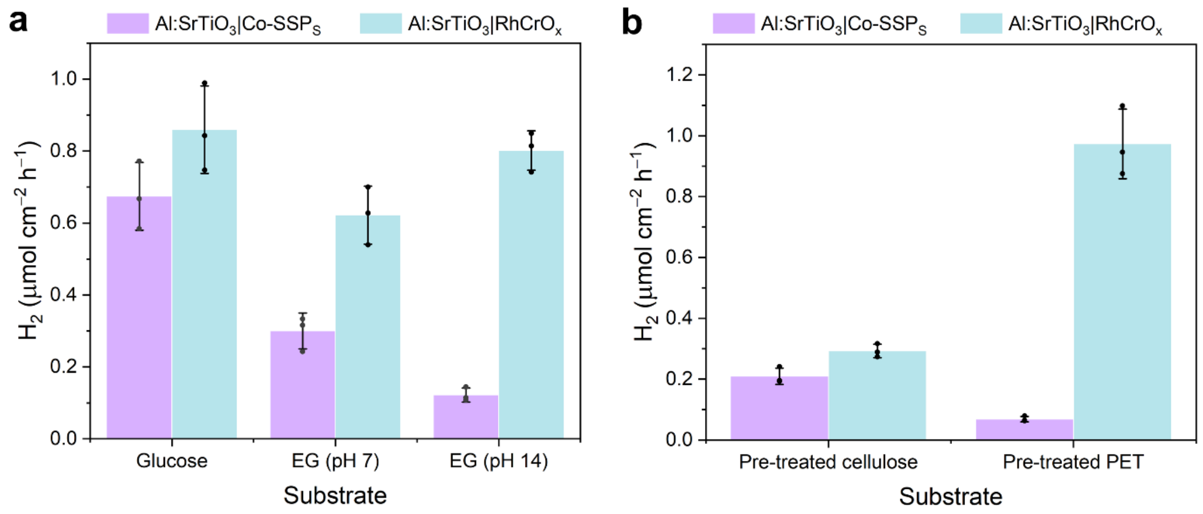

Supplementary Figure 42 | H_2 evolution comparison between Al:SrTiO$_3$|Co-SSP$_S$ and a benchmark Al:SrTiO$_3$|RhCrO$_x$ PC system. a, H_2 evolution with glucose and EG as model substrates. **b,** H_2 evolution with pre-treated cellulose and pre−treated PET as real-world waste-derived substrates. The photocatalytic experiments were performed under AM1.5G illumination for 22 h at room temperature. The data are presented as mean values ± SD for reactions performed in triplicate (n = 3).

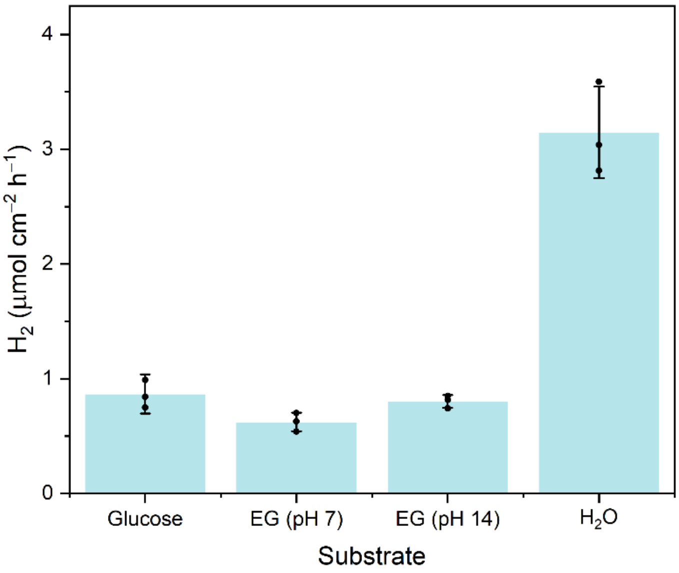

Supplementary Figure 43 | H_2 evolution comparison of benchmark Al:SrTiO$_3$|RhCrO$_x$ system using different oxidation substrates. The photocatalytic experiments were performed under AM1.5G illumination for 22 h at room temperature. The data are presented as mean values ± SD for reactions performed in triplicate (n = 3).

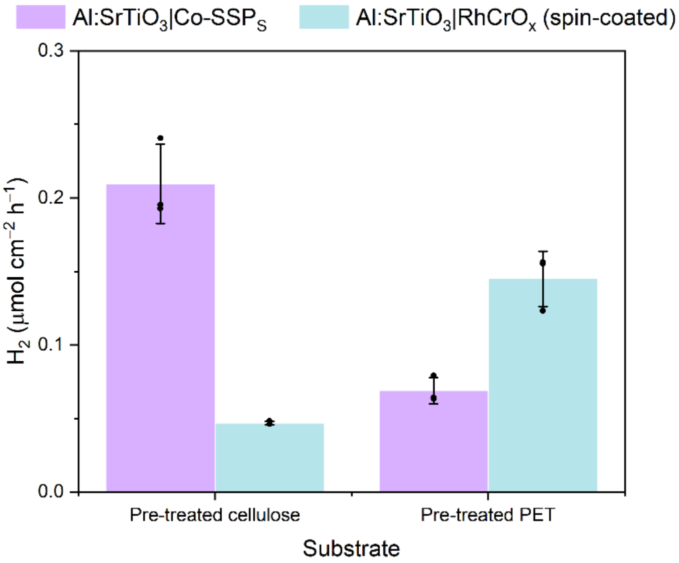

Supplementary Figure 44 | H_2 evolution comparison between Al:SrTiO$_3$|Co-SSP$_S$ and a Al:SrTiO$_3$|RhCrO$_x$ PC system with RhCrO$_x$ deposited using spin coating. While the benchmark RhCrO$_x$ co-catalyst is typically loaded using impregnation, spin coating was used in this case to study the performance of the co-catalysts using the same deposition technique. The photocatalytic experiments were performed under AM1.5G illumination for 22 h at room temperature. The data are presented as mean values ± SD for reactions performed in triplicate (n = 3).

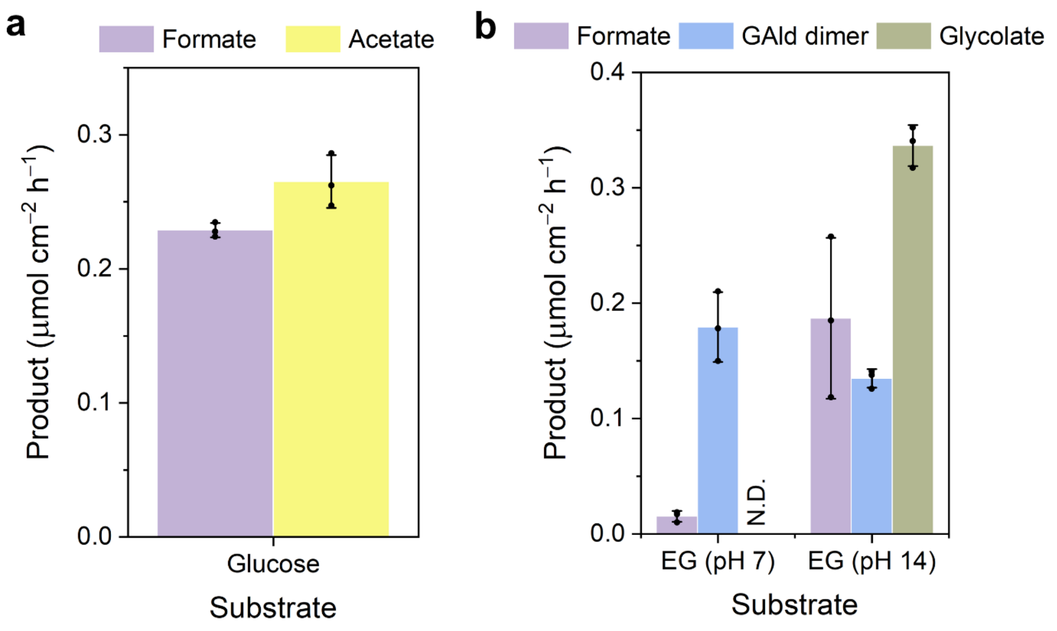

Supplementary Figure 45 | Oxidation products of Al:SrTiO$_3$|RhCrO$_x$ using model substrates. a,b, Oxidation products using glucose (**a**) and EG (**b**) as substrate. The photocatalytic experiments were performed under AM1.5G illumination for 22 h at room temperature. The data are presented as mean values ± SD for reactions performed in triplicate (n = 3).

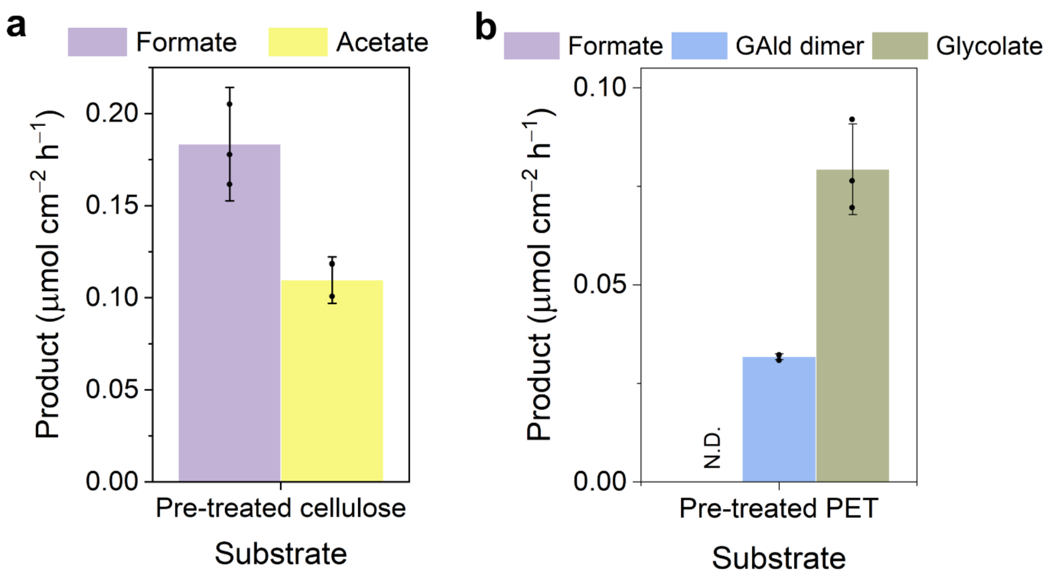

Supplementary Figure 46 | Oxidation products of Al:SrTiO$_3$|RhCrO$_x$ using real-world waste-derived substrates. a,b, Oxidation products using pre-treated cellulose (**a**) and pre-treated PET (**b**) as substrate. The photocatalytic experiments were performed under AM1.5G illumination for 22 h at room temperature. The data are presented as mean values ± SD for reactions performed in triplicate (n = 3).

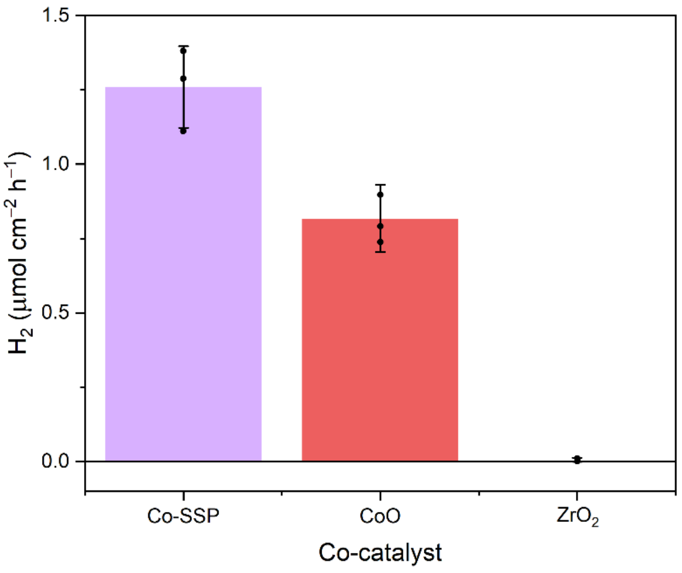

Supplementary Figure 47 | H_2 evolution comparison between Al:$SrTiO_3$ PC sheets using Co-SSP, CoO and ZrO_2 as co-catalysts. The metal oxides were deposited onto the PC sheets with Nafion as a binder without annealing. The photocatalytic experiments were performed under AM1.5G illumination for 22 h at room temperature. The data are presented as mean values ± SD for reactions performed in triplicate (n = 3).

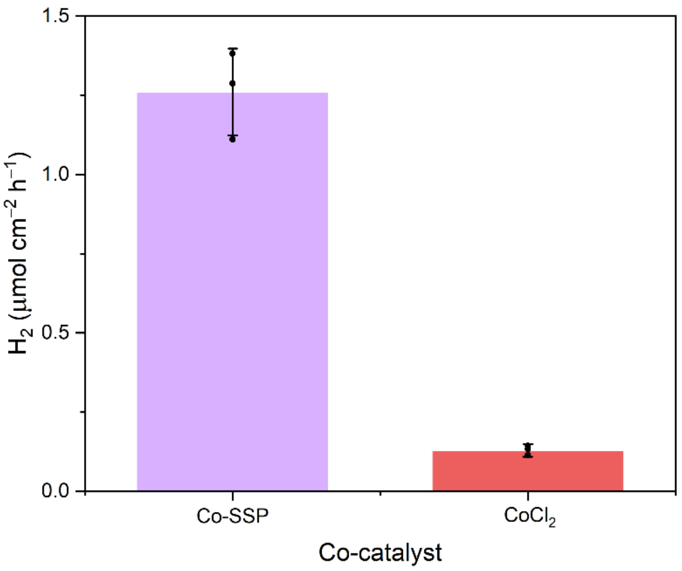

Supplementary Figure 48 | H_2 evolution comparison between Al:$SrTiO_3$ PC sheets using co-catalysts derived from single-source precursor (Co-SSP) and metal salt ($CoCl_2$). The $CoCl_2$ was deposited onto the PC sheet without a binder and annealing. The photocatalytic experiments were performed under AM1.5G illumination for 22 h at room temperature. The data are presented as mean values ± SD for reactions performed in triplicate (n = 3).

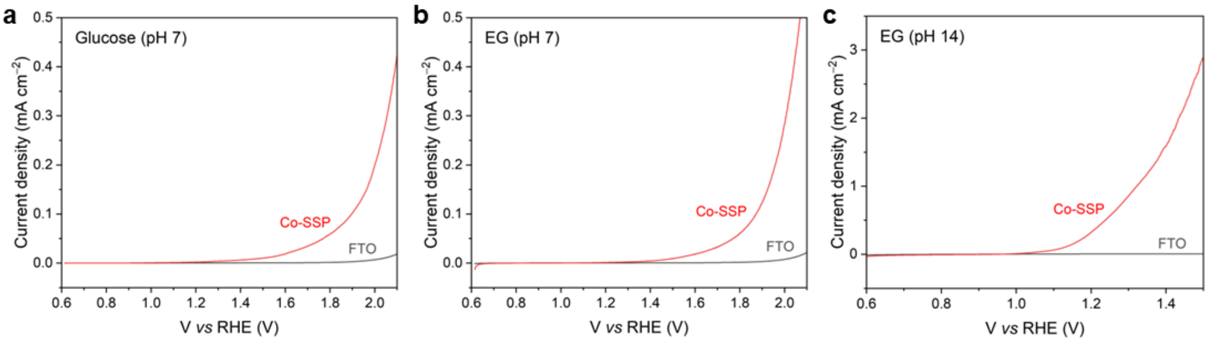

Supplementary Figure 49 | Evaluation of Co-SSP for electrochemical oxidation of glucose and EG. a–c Linear sweep voltammograms of SSP-modified FTO electrodes and bare FTO electrodes for glucose oxidation (**a**) and EG oxidation (**b**) at pH 7 as well as for EG oxidation at pH 14 (**c**). Electrochemical measurements were performed in 15 ml stirred Na_2SO_4 (0.1 M, pH) electrolyte containing either glucose (0.1 M) or EG (0.1 M). Alkaline electrolyte contains KOH (1.0 M, pH 14) and EG (0.1 M).

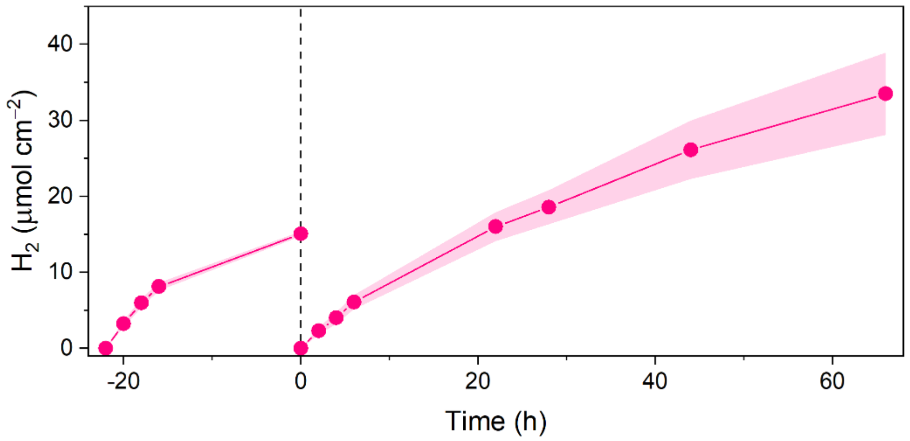

Supplementary Figure 50 | Long-term photocatalytic experiments with Al:$SrTiO_3$|Co-SSPs in reaction solution containing low concentrations (~0.1 mM) of Co^{2+} ions. The Co^{2+} ions in the reaction solution resulted from leached Co from a prior photocatalytic experiment also using an Al:$SrTiO_3$|Co-SSP PC sheet. The dotted line indicates when the used PC sheet was replaced with a fresh sheet for a second photocatalytic cycle. For clarity, 0 h is denoted as the start of the second photocatalytic cycle. The photocatalytic experiments were performed under AM1.5G illumination for a total of 88 h at room temperature using glucose as the substrate. The data are presented as mean values ± SD for reactions performed in triplicate (n = 3).

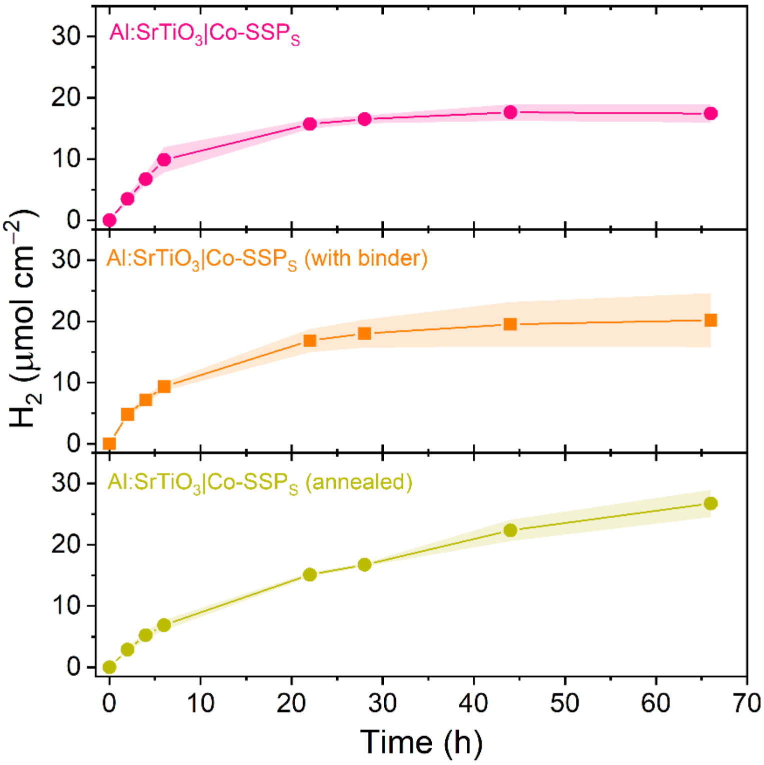

Supplementary Figure 51 | Long-term photocatalytic experiments with Al:SrTiO3|Co-SSPs, Al:SrTiO3|Co-SSPs with a Nafion binder, and Al:SrTiO3|Co-SSPs with annealing. The photocatalytic experiments were performed under AM1.5G illumination for 66 h at room temperature using glucose as the substrate. The data are presented as mean values ± SD for reactions performed in triplicate (n = 3).

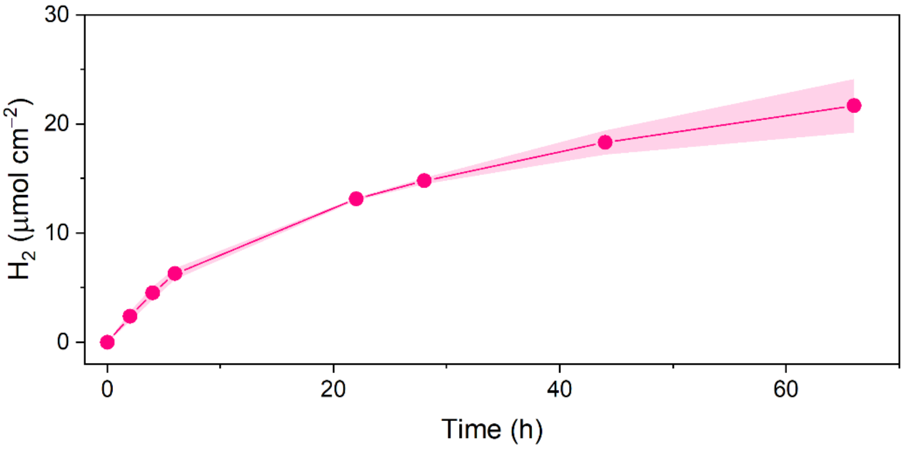

Supplementary Figure 52 | Long-term photocatalytic experiments with Al:SrTiO3|Co-SSPs in reaction solution containing 0.1 mM Zr^{4+} ions. The photocatalytic experiments were performed under AM1.5G illumination for a total of 66 h at room temperature using glucose as the substrate. The data are presented as mean values ± SD for reactions performed in triplicate (n = 3).

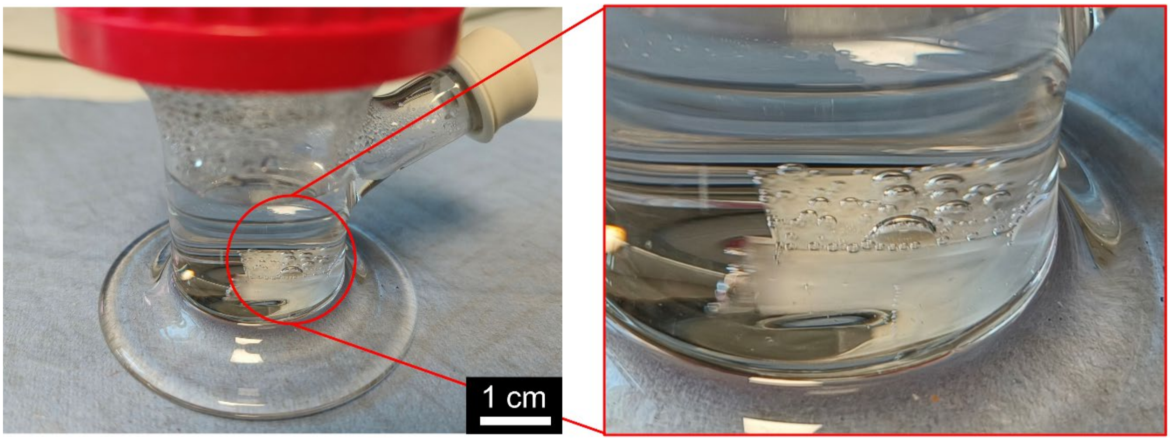

Supplementary Figure 53 | Photograph of Al:SrTiO3|Co-SSPs PC sheet with Nafion binder after photocatalytic experiment. The photograph shows evolved H_2 bubbles on the surface of the PC sheet.

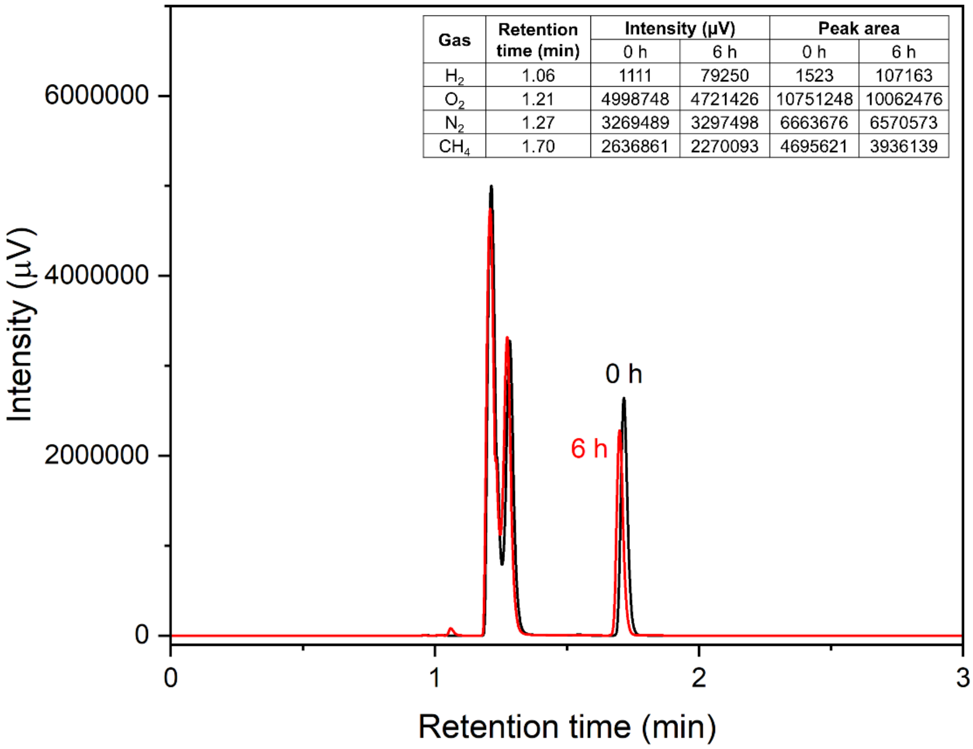

Gas	Retention time (min)	Intensity (μV)		Peak area	
		0 h	6 h	0 h	6 h
H_2	1.06	1111	79250	1523	107163
O_2	1.21	4998748	4721426	10751248	10062476
N_2	1.27	3269489	3297498	6663676	6570573
CH_4	1.70	2636861	2270093	4695621	3936139

Supplementary Figure 54 | Sample gas chromatograms from gas sampling at the beginning (0 h) and end (6 h) of the 1 m^2 outdoor experiment using glucose as the substrate. Inset: retention time, intensity and peak area of each detected gas. Large peaks corresponding to O_2 and N_2 gases were caused by unavoidable air contamination during gas injection into the GC.

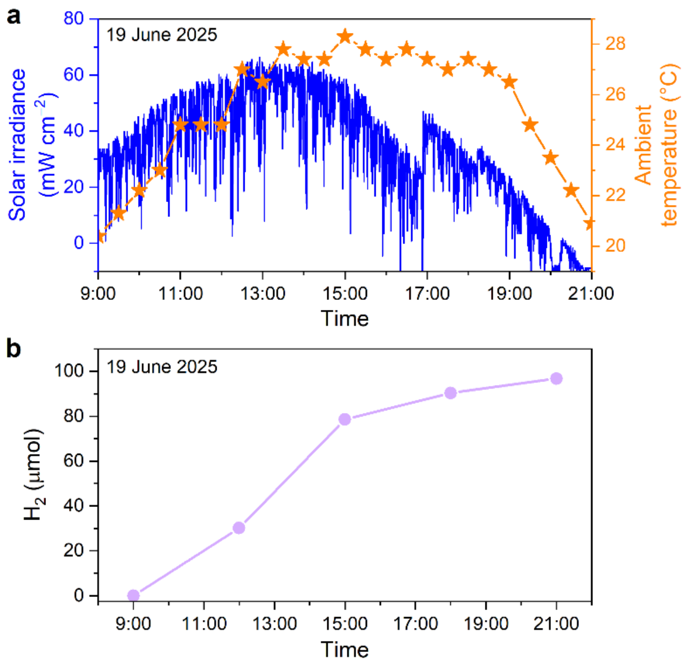

Supplementary Figure 55 | 12 h medium-scale outdoor experiments using Al:$SrTiO_3$|Co-SSP_M. a, Weather conditions over the course of the experiment on 19 June 2025. The average sunlight intensity was 0.33. **b,** Product formation from the outdoor demonstration under natural sunlight. Glucose was used as the substrate.

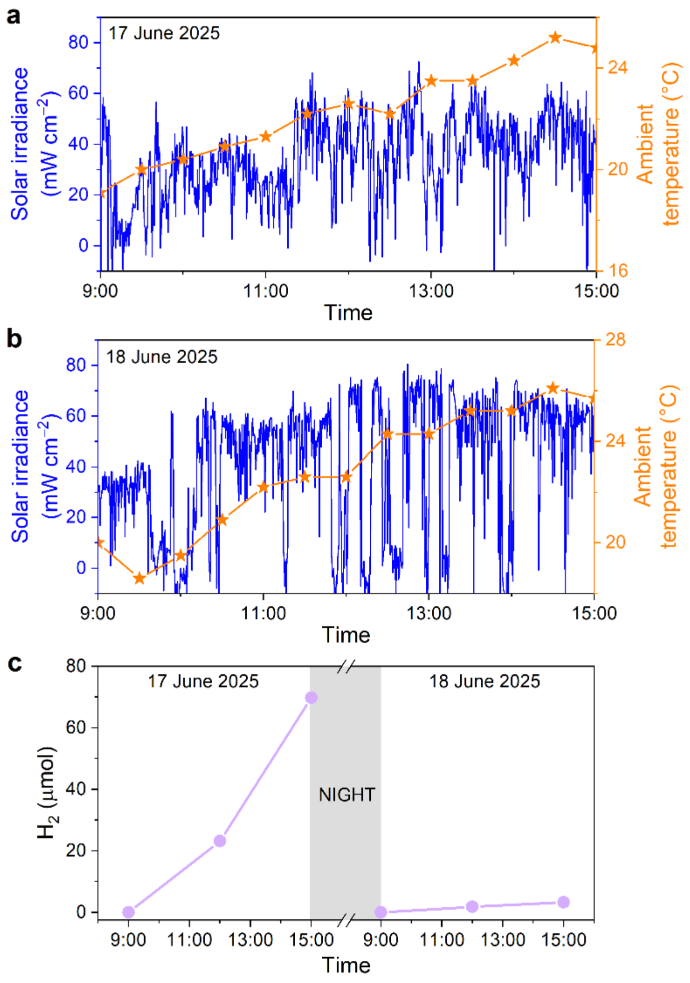

Supplementary Figure 56 | Overnight medium-scale outdoor experiments using Al:SrTiO$_3$|Co-SSP$_M$ with the PC sheet left in the reaction solution overnight. a,b, Weather conditions over the course of the experiment on 17 June 2025 (**a**) and 18 June 2025 (**b**). The average sunlight intensities each day were 0.34 and 0.41 suns, respectively. **c,** Product formation from the outdoor demonstration under natural sunlight. Glucose was used as the substrate.

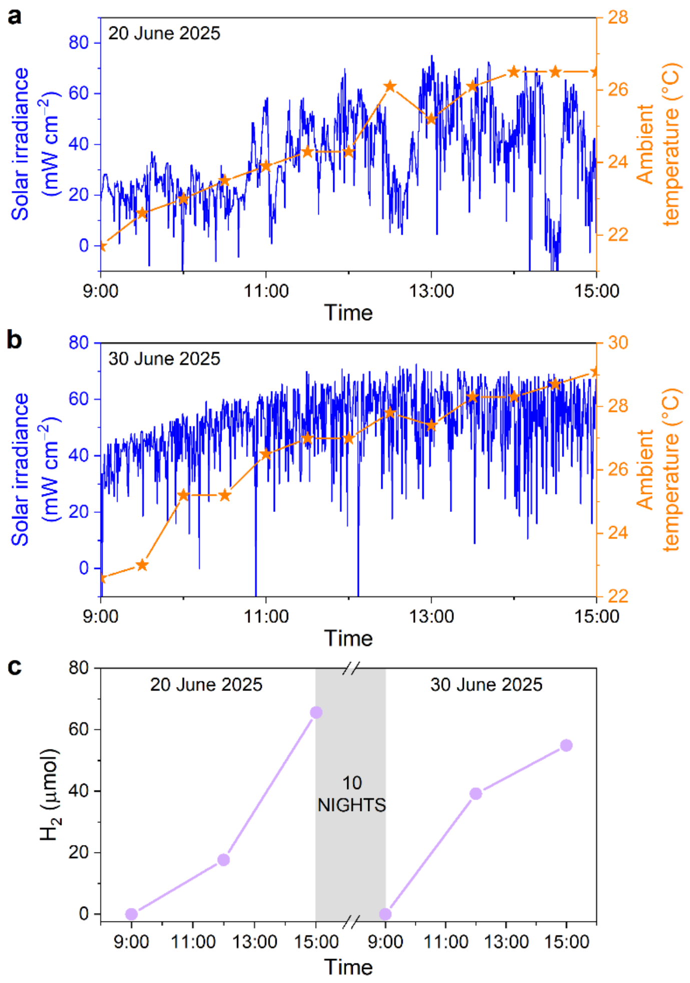

Supplementary Figure 57 | Overnight medium-scale outdoor experiments using Al:SrTiO$_3$|Co-SSP$_M$ with the PC sheet stored outside the reaction solution (i.e. in dry conditions) for 10 days in between each outdoor experiment. a,b, Weather conditions over the course of the experiment on 20 June 2025 (**a**) and 30 June 2025 (**b**). The average sunlight intensities each day were 0.35 and 0.51 suns, respectively. **c,** Product formation from the outdoor demonstration under natural sunlight. Glucose was used as the substrate.

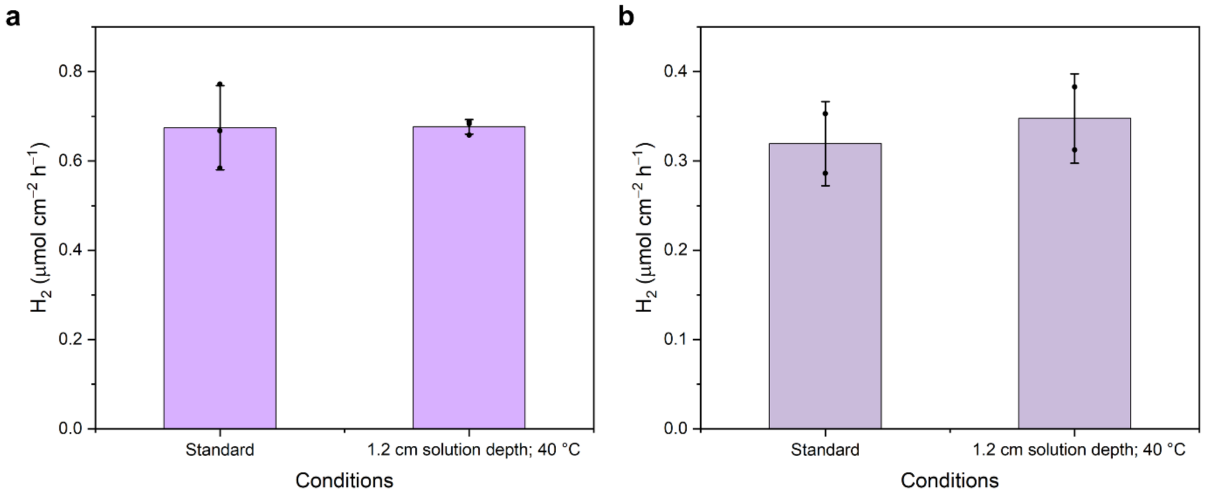

Supplementary Figure 58 | Performance comparison between photocatalytic experiments performed under standard conditions as well as with solution depth and temperature fixed at 1.2 cm and 40 °C, respectively. a,b, Performance comparison of Al:$SrTiO_3$|Co-SSP_S (**a**) and Al:$SrTiO_3$|Co-SSP_M (**b**) PC sheets under the different conditions. Standard conditions refer to a solution depth of ~1.4 and ~0.9 cm for the small (1 cm^2) and medium (20.25 cm^2) scale experiments, respectively, at room temperature. The photocatalytic experiments were performed in 0.1 M glucose solution under AM1.5G illumination for 22 h and 6 h for the small and medium scale experiments, respectively. The data in (**a**) and (**b**) are presented as mean values ± SD for reactions performed in triplicate (n = 3) and duplicate (n=2), respectively.

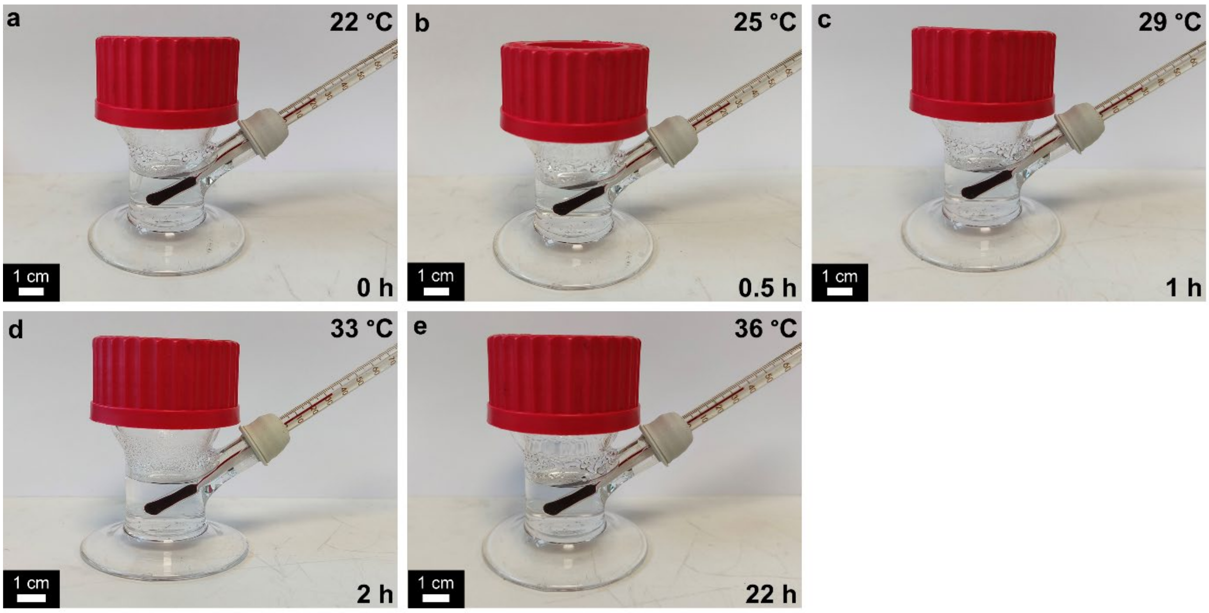

Supplementary Figure 59 | Temperature change during the small-scale experiments with Al:$SrTiO_3$|Co-SSPs. a-e, Temperature of reaction solution at 0 h (**a**), 0.5 h (**b**), 1 h (**c**), 2 h (**d**) and 22 h (**e**).

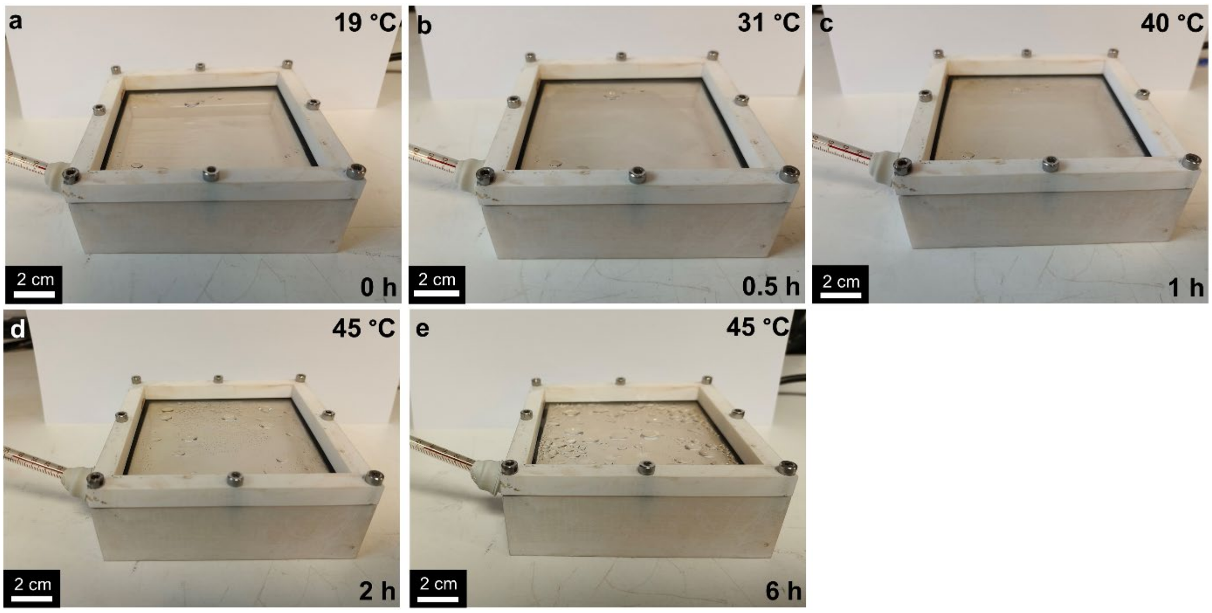

Supplementary Figure 60 | Temperature change during the medium-scale experiments with Al:$SrTiO_3$|Co-SSPs. a-e, Temperature of reaction solution at 0 h (**a**), 0.5 h (**b**), 1 h (**c**), 2 h (**d**) and 6 h (**e**).

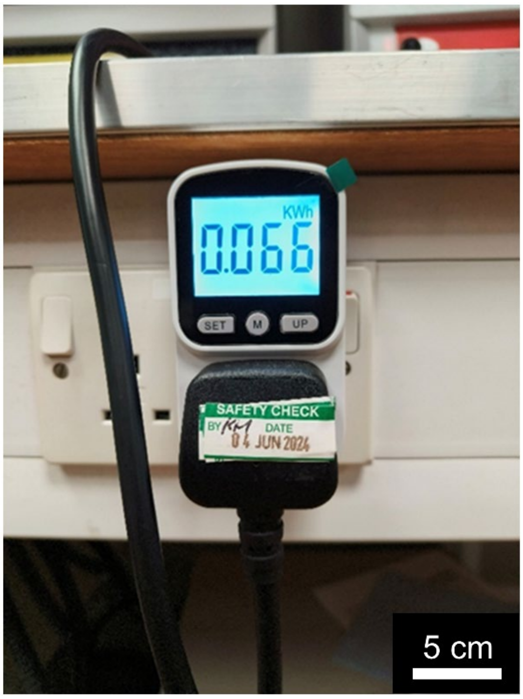

Supplementary Figure 61 | Photograph of energy meter used to measure energy consumption for catalyst synthesis.

Supplementary Table 1 | Crystallographic data and refinement of $Co_4Zr_2O(O^nPr)_{10}(acac)_4$.

	$Co_4Zr_2O(O^nPr)_{10}(acac)_4$
CCDC number	2416182
Cambridge data number	DW_K3_0080
Chemical formula	$C_{50}H_{98}Co_4O_{19}Zr_2$
Formula weight	1421.44
Temperature / K	180(2)
Crystal system	triclinic
Space group	P –1
a / Å	11.8592(4)
b / Å	12.9542(5)
c / Å	13.3577(6)
alpha / °	106.607(2)
beta / °	115.323(2)
gamma / °	101.396(3)
Unit-cell volume / $Å^3$	1651.93(12)
Z	1
Calc. density / g cm^{-3}	1.429
F(000)	738
Radiation type	Mo Kα
Absorption coefficient / mm^{-1}	1.346
Crystal size / mm^3	0.28 × 0.14 × 0.04
2-Theta range / °	7.03−50.70
Completeness to max 2-theta	0.963
No. of reflections measured	13968
No. of independent reflections	5823
R(int)	0.0502
No. parameters / restraints	370 / 39
Final R1 values (I > 2σ(I))	0.0520
Final wR(F^2) values (all data)	0.1264
Goodness-of-fit on F^2	1.042
Largest difference peak & hole / e $Å^{-3}$	0.845, −0.546

Supplementary Table 2 | Fabrication details of the $Al:SrTiO_3$|Co-SSP PC sheets.

PC sheet	Fabrication details		
	Support	$Al:SrTiO_3$ deposition method	Co-SSP deposition method
$Al:SrTiO_3$|Co-SSP_S	1 cm^2 glass	Drop casting and annealing	Spin coating
$Al:SrTiO_3$|Co-SSP_M	20.25 cm^2 frosted glass	Spray coating	Spray coating
$Al:SrTiO_3$|Co-SSP_L	0.25 m^2 frosted glass	Spray coating	Spray coating

Supplementary Table 3 | H_2 evolution of Al:$SrTiO_3$|Co-SSPs with various Al:$SrTiO_3$ loadings. The photocatalytic experiments were performed under AM1.5G illumination for 22 h at room temperature with TEOA. The data are presented as mean values ± SD for reactions performed in triplicate (n = 3).

Al:$SrTiO_3$ loading (mg cm^{-2})	H_2 (μmol cm^{-2} h^{-1})
0.5	0.64±0.01
1.0	0.79±0.02
1.5	1.02±0.06
2.0	1.25±0.19
2.5	1.20±0.07

Supplementary Table 4 | Calculated and actual loadings of Co and Zr species on Al:$SrTiO_3$|Co-SSPs. The calculated values were based on the volume and concentration of Co-SSP used and the actual values were determined via ICP-OES.

Spin coated Co-SSP concentration (M)	Calculated pre-catalysis loading (wt%)		Measured pre-catalysis loading (wt%)		Measured post-catalysis loading (wt%)	
	Co	Zr	Co	Zr	Co	Zr
0	0	0	0	0	0	0
0.02	7.07	5.50	0.53	0.42	0.36	0.06
0.04	14.14	11.00	1.01	0.84	0.40	0.09
0.06	21.20	16.50	1.31	1.20	1.01	0.10
0.08	28.27	22.00	1.56	1.34	1.04	0.10
0.10	35.34	27.50	1.97	1.76	2.02	0.11

Supplementary Table 5 | H_2 evolution of 1 cm^2 Al:$SrTiO_3$ PC sheets with various Co-SSP loadings. The Co and Zr loadings are shown in Supplementary Table 2. The photocatalytic experiments were performed under AM1.5G illumination for 22 h at room temperature. The data are presented as mean values ± SD for reactions performed in triplicate (n = 3).

Co-SSP concentration	H_2 (μmol cm^{-2} h^{-1})
0	0.04±0.0007
0.02	0.88±0.09
0.04	1.26±0.14
0.06	1.08±0.07
0.08	1.04±0.003
0.1	0.90±0.09

Supplementary Table 6 | H_2 evolution comparison between Al:$SrTiO_3$ PC sheets with Co-SSP deposited via different methods. The photocatalytic experiments were performed under AM1.5G illumination for 22 h at room temperature. The data are presented as mean values ± SD for reactions performed in triplicate (n = 3).

Co-SSP deposition method	H_2 (μmol cm^{-2} h^{-1})
Spin coating	1.26±0.14
Drop casting	0.18±0.03

Supplementary Table 7 | IQE of the PC sheets at 350 nm with different Co-SSP deposition methods. Glucose was used as the substrate.

Co-SSP deposition method	IQE (%)
Spin coating	3.75
Spray coating	3.80
Drop casting	2.29

Supplementary Table 8 | H_2 evolution of Al:$SrTiO_3$|Co-SSP_S and Co-SSP_S only. The photocatalytic experiments were performed under AM1.5G illumination for 22 h at room temperature. N.D., not detected. The data are presented as mean values ± SD for reactions performed in triplicate (n = 3).

PC sheet	H_2 (μmol cm^{-2} h^{-1})
Al:$SrTiO_3$|Co-SSP_S	1.26±0.14
Co-SSP_S	N.D.

Supplementary Table 9 | H_2 evolution comparison between Al:$SrTiO_3$|Co-SSP_S, Al:$SrTiO_3$ only and Al:$SrTiO_3$|$RhCrO_x$ with glucose and EG as model substrates. The photocatalytic experiments were performed under AM1.5G illumination for 22 h at room temperature. N.D., not detected. The data are presented as mean values ± SD for reactions performed in triplicate (n = 3).

PC sheet	Substrate	H_2 (μmol cm^{-2} h^{-1})
Al:$SrTiO_3$|Co-SSP_S	Glucose	0.67±0.09
	EG (pH 7)	0.30±0.05
	EG (pH 14)	0.12±0.02
Al:$SrTiO_3$	Glucose	0.01±0.001
	EG (pH 7)	N.D.
	EG (pH 14)	N.D.
Al:$SrTiO_3$|$RhCrO_x$	Glucose	0.86±0.12
	EG (pH 7)	0.62±0.08
	EG (pH 14)	0.80±0.06

Supplementary Table 10 | PEIS fitting results. Error bars represent the fitting errors. Measurements were performed in 30 ml stirred electrolyte containing 0.1 M Na_2SO_4 and 0.1 M substrate (TEOA, glucose, or ethylene glycol) under AM1.5G illumination.

Working electrode	Substrate	R_{ct} (Ω cm^2)	C_{SCLJ} (F cm^{-2})	k_{ct} (s^{-1})
Al:$SrTiO_3$	Glucose	13436±253.6	$1.99\times10^{-5}\pm2.63\times10^{-7}$	3.7
Al:$SrTiO_3$|Co-SSP_S	Glucose	1646±42.4	$2.94\times10^{-5}\pm2.04\times10^{-6}$	20.7
Al:$SrTiO_3$	EG	20042±188.6	$1.46\times10^{-5}\pm8.60\times10^{-8}$	3.4
Al:$SrTiO_3$|Co-SSP_S	EG	2723±51.5	$2.29\times10^{-5}\pm8.05\times10^{-7}$	16.0
Al:$SrTiO_3$	TEOA	4472±47.9	$2.79\times10^{-5}\pm3.78\times10^{-7}$	8.0
Al:$SrTiO_3$|Co-SSP_S	TEOA	412.1±2.4	$1.86\times10^{-5}\pm6.30\times10^{-7}$	130.6

Supplementary Table 11 | Time-course H_2 evolution of Al:$SrTiO_3$|Co-SSPs with real-world waste-derived substrates and without substrates. The photocatalytic experiments were performed under AM1.5G illumination for 22 h at room temperature. The data are presented as mean values ± SD for reactions performed in triplicate (n = 3).

Substrate	Time (h)	H_2 (μmol cm^{-2})
Pre-treated cellulose	0	0
	2	1.22±0.22
	4	1.86±0.16
	6	2.80±0.29
	10	3.97±0.27
	22	4.61±0.59
Pre-treated PET	0	0
	2	0.35±0.07
	4	0.66±0.06
	6	0.72±0.23
	10	0.92±0.11
	22	1.51±0.19
H_2O (no substrate)	0	0
	2	0.11±0.04
	4	0.13±0.03
	6	0.17±0.05
	10	0.26±0.13
	22	0.32±0.12

Supplementary Table 12 | Chemical equations and associated Gibbs free energies of glucose and EG reforming.[42,43]

Reaction	Chemical equation	ΔG^0 (kJ mol^{-1})
Glucose reforming	$C_6H_{12}O_6$ (s) + $6H_2O$ (l) → $12H_2$ (g) + $6CO_2$ (g)	−84.7
EG reforming	$C_2H_6O_2$ (l) + $2H_2O$ (l) → $5H_2$ (g) + $2CO_2$ (g)	+9.2

Supplementary Table 13 | H_2 evolution comparison between Al:$SrTiO_3$|Co-SSPs and Al:$SrTiO_3$|$RhCrO_x$ with real-world waste-derived substrates. The photocatalytic experiments were performed under AM1.5G illumination for 22 h at room temperature. The data are presented as mean values ± SD for reactions performed in triplicate (n = 3).

PC sheet	Substrate	H_2 (μmol cm^{-2} h^{-1})
Al:$SrTiO_3$|Co-SSPs	Pre-treated cellulose	0.21±0.03
	Pre-treated PET	0.07±0.01
Al:$SrTiO_3$|$RhCrO_x$	Pre-treated cellulose	0.29±0.02
	Pre-treated PET	0.97±0.11

Supplementary Table 14 | Oxidation products of Al:SrTiO$_3$|Co-SSP$_S$ and Al:SrTiO$_3$|RhCrO$_x$ using real-world waste-derived substrates. The photocatalytic experiments were performed under AM1.5G illumination for 22 h at room temperature. N.D., not detected. Grey boxes indicate that the samples were not tested for the target product. The data are presented as mean values ± SD for reactions performed in triplicate (n = 3).

PC sheet	Substrate	Formate (μmol cm^{-2} h^{-1})	Acetate (μmol cm^{-2} h^{-1})	GAld dimer (μmol cm^{-2} h^{-1})	Glycolate (μmol cm^{-2} h^{-1})
Al:SrTiO$_3$| Co-SSP$_S$	Pre-treated cellulose	0.15±0.02	0.07±0.01		
	Pre-treated PET	N.D.		0.03±0.001	0.08±0.01
Al:SrTiO$_3$| RhCrO$_x$	Pre-treated cellulose	0.18±0.03	0.11±0.01		
	Pre-treated PET	N.D.		0.11±0.003	0.34±0.04

Supplementary Table 15 | Oxidation products of Al:SrTiO$_3$|Co-SSP$_S$ and Al:SrTiO$_3$|RhCrO$_x$ with glucose and EG as model substrates. The photocatalytic experiments were performed under AM1.5G illumination for 22 h at room temperature. N.D., not detected. Grey boxes indicate that the samples were not tested for the target product. The data are presented as mean values ± SD for reactions performed in triplicate (n = 3).

PC sheet	Substrate	Formate (μmol cm^{-2} h^{-1})	Acetate (μmol cm^{-2} h^{-1})	GAld dimer (μmol cm^{-2} h^{-1})	Glycolate (μmol cm^{-2} h^{-1})
Al:SrTiO$_3$| Co-SSP$_S$	Glucose	0.21±0.01	0.43±0.02		
	EG (pH 7)	0.03±0.01		0.05±0.002	N.D.
	EG (pH 14)	0.11±0.04		0.14±0.01	0.08±0.02
Al:SrTiO$_3$| RhCrO$_x$	Glucose	0.23±0.005	0.27±0.02		
	EG (pH 7)	0.02±0.004		0.18±0.03	N.D.
	EG (pH 14)	0.19±0.07		0.13±0.01	0.34±0.02

Supplementary Table 16 | Time-course H_2 evolution of Al:$SrTiO_3$ during photocatalyst reusability tests. Co-SSP was re-deposited onto the Al:$SrTiO_3$|Co-SSP_S PC sheets after each cycle. The photocatalytic experiments were performed under AM1.5G illumination for 22 h at room temperature each cycle. The data are presented as mean values ± SD for reactions performed in triplicate (n = 3).

Substrate	Cycle 1		Cycle 2		Cycle 3	
	Time (h)	H_2 (μmol cm^{-2})	Time (h)	H_2 (μmol cm^{-2})	Time (h)	H_2 (μmol cm^{-2})
Glucose	0	0	22	0	44	0
	2	3.59±0.44	24	4.4±0.95	46	3.63±0.44
	4	7.31±1.64	26	8.36±1.05	48	7.73±0.88
	6	9.50±1.33	28	9.65±1.50	50	10.11±1.49
	10	12.76±1.98	32	11.43±1.21	54	12.96±2.18
	22	14.44±1.51	44	12.04±1.36	66	16.59±2.52
Pre-treated cellulose	0	0	22	0	44	0
	2	1.22±0.22	24	0.63±0.01	46	0.56±0.08
	4	1.86±0.16	26	0.84±0.01	48	0.60±0.04
	6	2.80±0.29	28	0.99±0.01	50	0.56±0.05
	10	3.97±0.27	32	1.15±0.05	54	0.60±0.08
	22	4.61±0.59	44	1.40±0.11	66	0.70±0.07

Supplementary Table 17 | H_2 evolution of Al:$SrTiO_3$|Co-SSP_S under various light intensities using pre-treated cellulose as the substrate. The photocatalytic experiments were performed for 22 h at room temperature. The data are presented as mean values ± SD for reactions performed in triplicate (n = 3).

Light intensity (Sun)	H_2 (μmol cm^{-2} h^{-1})
0.1	0.01±0.006
0.5	0.10±0.01
0.7	0.12±0.004
1	0.21±0.03

Supplementary Table 18 | Oxidation products of Al:$SrTiO_3$|Co-SSP_S under various light intensities using pre-treated cellulose as the substrate. The photocatalytic experiments were performed for 22 h at room temperature. The data are presented as mean values ± SD for reactions performed in triplicate (n = 3).

Light intensity (Sun)	Formate (μmol cm^{-2} h^{-1})	Acetate (μmol cm^{-2} h^{-1})
0.1	0.03±0.003	0.005±0.003
0.5	0.07±0.001	0.05±0.004
0.7	0.12±0.004	0.06±0.008
1	0.15±0.02	0.07±0.01

Supplementary Table 19 | Performance of Al:SrTiO$_3$|Co-SSP$_S$ from 25−70 °C. The photocatalytic experiments were performed in 0.1 M glucose solution under AM1.5G illumination for 6 h with the solution depth fixed at 1.2 cm. The data are presented as mean values ± SD for reactions performed in triplicate (n = 3).

Temperature (°C)	H_2 (μmol cm^{-2} h^{-1})
25	0.79±0.11
40	0.68±0.02
55	0.73±0.08
70	0.68±0.09

Supplementary Table 20 | Loadings of Co and Zr species on Al:SrTiO$_3$|Co-SSP$_M$. The values were determined via ICP-OES.

Spray coated Co-SSP volume (μl cm^{-2})	Measured pre-catalysis loading (wt%)		Measured post-catalysis loading (wt%)	
	Co	Zr	Co	Zr
2.5	2.99	1.21	0.89	0.23
5.0	9.34	5.34	0.90	0.26
7.5	12.46	7.58	0.94	0.15
10.0	16.02	9.54	1.30	0.51
12.5	19.42	11.76	1.69	0.16

Supplementary Table 21 | H_2 evolution of 20.25 cm^2 Al:SrTiO$_3$ PC sheets with various volumes of Co-SSP spray coated. The photocatalytic experiments were performed under AM1.5G illumination for 6 h. The data are presented as mean values ± SD for reactions performed in triplicate (n = 3).

Spray coated volume (μl cm^{-2})	H_2 (μmol cm^{-2} h^{-1})
2.5	0.63±0.17
5.0	0.64±0.16
7.5	0.63±0.25
10.0	0.58±0.08
12.5	0.57±0.03

Supplementary Table 22 | Comparison between H_2 evolution of Al:SrTiO$_3$|Co-SSP$_M$ and Al:SrTiO$_3$|Co-SSP$_S$. The photocatalytic experiments were performed under AM1.5G illumination for 22 h and 6 h, respectively, for the small- and medium-scale PC sheets. The data are presented as mean values ± SD for reactions performed in triplicate (n = 3).

PC sheet	H_2 (μmol cm^{-2} h^{-1})	H_2 (μmol g_{cat}^{-1} h^{-1})
Al:SrTiO$_3$|Co-SSP$_M$	0.63±0.17	2870±780
Al:SrTiO$_3$|Co-SSP$_S$	1.26±0.14	630±69

Supplementary Table 23 | Performance comparison between Al:$SrTiO_3$|Co-SSP_M and 20.25 cm^2 Al:$SrTiO_3$|Co-SSP_S PC sheets. The photocatalytic experiments were performed under AM1.5G illumination for 6 h. The data are presented as mean values ± SD for reactions performed in triplicate (n = 3).

PC sheet	H_2 (μmol cm^{-2} h^{-1})
Al:$SrTiO_3$|Co-SSP_M	0.63±0.17
Al:$SrTiO_3$|Co-SSP_S (20.25 cm^2)	0.81±0.31

Supplementary Table 24 | Performance of Al:$SrTiO_3$|Co-SSP_M from 25−70 °C. The photocatalytic experiments were performed in 0.1 M glucose solution under AM1.5G illumination for 6 h with the solution depth fixed at 1.2 cm. The data are presented as mean values ± SD for reactions performed in triplicate (n = 3).

Temperature (°C)	H_2 (μmol cm^{-2} h^{-1})
25	0.31±0.01
40	0.35±0.05
55	0.33±0.02
70	0.34±0.003

Supplementary Table 25 | Incident sunlight intensity during the outdoor 1 m^2 demonstration.

Date	Substrate	Incident sunlight intensity (mW cm^{-2})		
		Minimum	Maximum	Average
30 August 2024	Pre-treated cellulose	7.1	82.3	50.8±20.2
28 September 2024	Glucose	15.0	93.8	60.7±20.2
4 October 2024	Glucose (photocatalyst recycle)	23.9	92.8	51.4±27.8

Supplementary Table 26 | Product formation from the outdoor 1 m^2 demonstration. The photocatalytic experiments were performed under natural sunlight for 6 h.

Date	Substrate	H_2 (mmol m^{-2})	Formate (mmol m^{-2})	Acetate (mmol m^{-2})
30 August 2024	Pre-treated cellulose	1.51	1.50	0.94
28 September 2024	Glucose	5.24	2.68	1.76
4 October 2024	Glucose (photocatalyst recycle)	4.71	2.53	1.17

Supplementary Table 27 | Total organic carbon from the outdoor 1 m^2 demonstration. Calculated with respect to the substrate glucose as well as the primary oxidation products formate and acetate. The difference in total organic carbon before and after reaction was due to the presence of intermediate oxidation products which were not quantified. The total organic carbon after the reaction on 28 September 2024 is the same as before the reaction on 4 October 2024 because the same feedstock was reused for the photocatalyst reuse experiment.

Date	Substrate	Total organic carbon (ppm)	
		Before reaction	After reaction
30 August 2024	Pre-treated cellulose	790.56	784.74
28 September 2024	Glucose	792.00	764.04
4 October 2024	Glucose (photocatalyst recycle)	764.04	750.98

Supplementary Table 28 | Reports on photocatalyst panel systems.

Photocatalytic system	Panel size	Light source	Substrate	Other conditions	Areal production rate (mmol H_2 m^{-2} h^{-1})	Ref.
**Photocatalytic reforming systems**						
Al:$SrTiO_3$|Co-SSP_S	1 cm^2	Simulated sunlight	TEOA	-	12.5	This work
			Glucose	-	6.74	
			EG	Water	3.00	
				1.0 M KOH	1.20	
			Cellulose	0.1 M $NaHCO_3$; cellulase	2.10	
			PET	1.0 M KOH	0.689	
Al:$SrTiO_3$|Co-SSP_M	20 cm^2	Simulated sunlight	TEOA	-	6.31	This work
Al:$SrTiO_3$|Co-SSP_L	1 m^2	Natural sunlight	Glucose	-	0.873	This work
			Cellulose	0.1 M $NaHCO_3$; cellulase	0.252	
CN_x|Ni_2P	25 cm^2	Simulated sunlight	Cellulose	0.5 M KOH	0.038	41
			MSW	0.5 M KOH	0.150	
			PET	0.5 M KOH	0.052	
			TEOA	-	0.750	
mpg−CN_x|Pt	0.756 m^2	Natural sunlight	TEOA	Nafion binder for catalyst	2.79	44
Melamine-cyanuric acid-derived CN_x|Pt	25 cm^2	100 W LED; 268 mW cm^{-2}	Ethanol	Continuous-flow reaction	0.584	45
Pt-loaded covalent triazine framework	19 cm^2	300 W Xe lamp; 130 mW cm^{-2}	TEOA	-	5.4	46
**Photocatalytic water splitting systems**						
Al:$SrTiO_3$|Rh/ Cr_2O_3/CoOOH	100 m^2	Natural sunlight	Water	Co-immobilisation with Si nanoparticles	22.7	4
Al:$SrTiO_3$| $RhCrO_x$/CoO_y	1 m^2	Natural sunlight	Water	30-minute test	42.3	5
Cr_2O_3/Ru-loaded La,Rh:$SrTiO_3$/ Au/Mo:$BiVO_4$	7.5 cm^2	Simulated sunlight	Water	53°C; 10 kPa	200000	47
Cr_2O_3/Ru-loaded La,Rh:$SrTiO_3$/ C/Mo:$BiVO_4$	9 cm^2	Simulated sunlight	Water	53°C; 91 kPa	100	48
Cr_2O_3/Ru-loaded Rh:$SrTiO_3$/ITO/ Mo:$BiVO_4$	6.25 cm^2	Simulated sunlight	Water	15°C; 91 kPa	50	49
$RhCrO_x$-loaded $LaMg_{1/3}Ta_{2/3}O_2N$/Au/ Mo:$BiVO_4$	9 cm^2	300 W Xe lamp	Water	Amorphous TiO_2 protective layer	1.25	50
$(Ga_{1-x}Zn_x)(N_{1-x}O_x)$| $Rh_{2-y}Cr_yO_3$	25 cm^2	300 W Xe lamp	Water	Co-immobilisation with Si nanoparticles	45.7	51

Al:$SrTiO_3$| $RhCrO_x$	25 cm^2	Simulated sunlight	Seawater	Catalyst supported on floating solar vapour generator	9.87	52

TEOA = Triethanolamine
EG = Ethylene glycol
PET = Polyethylene terephthalate
MSW = Municipal solid waste

Supplementary Table 29 | Performance comparison of real and hypothetical single-light absorber PC systems. The performance of the systems is compared in terms of their active area, areal production rate and stability.

Photocatalytic system	Substrate	Active area (m^2)	Areal production rate (mmol H_2 m^{-2} h^{-1})	Stability (h)	Ref
Al:$SrTiO_3$|CO-SSP_S	Glucose	0.0001	6.74	22	This work
Al:$SrTiO_3$|CO-SSP_L	Glucose	1	0.873	6	This work
**Hypothetical systems**					
TiO_2|Fe_2O_3	Water	70540	590	43800	33
CN_x|Ni_2P	MSW	400	1490	87600	34
TiO_2|Pt	PET	6000	2110	175200	2
TiO_2|Pt	Unspecified organics	6518	153	8760	35
**Real systems**					
CN_x|Ni_2P	Cellulose	0.0025	0.038	20	41
	MSW		0.150		
	PET		0.052		
	TEOA		0.750		
mpg-CN_x|Pt	TEOA	0.756	2.79	720	44
Melamine-cyanuric acid-derived CN_x|Pt	Ethanol	0.0025	0.584	4	45
Pt/IrO_2−loaded 3D CN_x nanosheets	Water	0.0001	9.55	100	53
CN_x|Ni/Ag	Ethanol	0.0005	3.54	7	54
CN_x|NiS	TEOA	0.0028	17.2	4	55
Pt-loaded covalent triazine framework	TEOA	0.0019	5.4	24	46
Al:$SrTiO_3$|Rh/ Cr_2O_3/CoOOH	Water	100	22.7	4320	4
Al:$SrTiO_3$| $RhCrO_x$/CoO_y	Water	1	42.3	1000	5
($Ga_{1-x}Zn_x$) ($N_{1-x}O_x$)| $Rh_{2-y}Cr_yO_3$	Water	0.0025	45.7	7	51
Al:$SrTiO_3$| $RhCrO_x$	Seawater	0.0025	9.87	154	52
TiO_2|Pt	Water	0.0011	88.32	20	56
TiO_2|NiCu	Ethanol	0.0000785	2.50	30	57

MSW = Municipal solid waste
PET = Polyethylene terephthalate
TEOA = Triethanolamine

Supplementary Table 30 | Values used in the technoeconomic analysis of the 1 m^2-scale system.[a,b]

Component	Cost (£)	Per unit	Ref
**Capital costs[c]**			
Acrylic cell chamber	57.05	m^2	[58]
Gasket	25.13	m^2	[59]
Aluminium support frame	63.30	m^2	[60]
Steel crossbeam support	8.94	m	[61]
Timber base	30.22	m^3	[62]
Aluminium rectangular tube for reactor base	13.80	m	[63]
Steel tube for reactor base	3.95	m	[64]
0.25 m^2 glass panels	22.20	unit	[65]
Toggle clamps	2.95	unit	[66]
Tube adaptors	1.76	unit	[67]
PVC tubing	0.48	m	[68]
Miscellaneous (fittings, bearings, wheels, etc)	93.08	-	-
Installation labour	29.30	m^2	[69]
**Operation/consumables costs**			
Al:$SrTiO_3$	0.011	g	Supplementary Table 31 (below)
Co-SSP	0.24	g	
Cellulase	0.0077	g	[70]
Cellulose	0.0020	g	[71]
Glucose	0.0011	g	[72]
$NaHCO_3$	0.00023	g	[73]
H_2O	0.0011	L	[74]
N_2 (for reactor purging)	0.0050	L	[75]
Energy (for catalyst synthesis)	0.22	kWh	[76]

[a] For accuracy, the scope of the technoeconomic analysis is limited to the actual operation of the photoreactor, i.e. the production cost of H_2 was calculated based on the volume of H_2 produced and the associated capital and operational costs in the actual as-conducted experiment.
[b] Currency conversion is based on rates on 11 September 2024.
[c] Capital costs are amortised over the estimated useful lifetime of the photoreactor, i.e. 100 days.

Supplementary Table 31 | Values used in calculating the price per unit of catalysts.

Chemical	Cost (£)	Per unit	Ref
**Al:$SrTiO_3$**			
Al_2O_3	0.0065	g	[77]
$SrTiO_3$	0.0019	g	[78]
$SrCl_2$•$6H_2O$	0.00061	g	[79]
Total	0.011	g	-
**Co-SSP**			
$Zr(O^nPr)_4$, 70 wt% in n-propanol	0.011	g	[80]
$Co(acac)_2$	0.0015	g	[81]
Toluene	0.0035	ml	[82]
Hexane	0.0054	ml	[83]
Tetrahydrofuran	0.0011	ml	[84]
Total	0.24	g	-

Supplementary Table 32 | Individual component contributions to capital and operation/consumables costs.

Component	Quantity	Cost (£)
**Capital costs**		
Acrylic cell chamber	3.00	170.99
Gasket	0.14	3.56
Aluminium support frame	0.21	13.44
Steel crossbeam support	2.36	21.10
Timber base	0.02	0.56
Aluminium rectangular tube for reactor base	4.20	57.96
Steel tube for reactor base	1.40	5.53
0.25 m^2 glass panels	4.00	88.80
Toggle clamps	40	118.00
Tube adaptors	10	17.60
PVC tubing	4.72	2.27
Miscellaneous (fittings, bearings, wheels, etc)	-	93.08
Installation labour	1.69	49.52
Total	-	642.40
**Operation/consumables costs (Pre−treated cellulose as substrate)**		
$Al:SrTiO_3$	1.82	0.020
Co-SSP	1.42	0.33
Cellulase	125.00	0.96
Cellulose	625.00	1.25
Glucose	49.54	0.054
$NaHCO_3$	210.03	0.048
H_2O	25.00	0.028
N_2 (for reactor purging)	50.00	0.25
Energy[a]	8.47	1.86
Total	-	4.81
**Operation/consumables costs (Glucose as substrate)**		
$Al:SrTiO_3$	1.82	0.020
Co-SSP	1.42	0.33
Glucose	49.54	0.054
H_2O	25.00	0.028
N_2 (for reactor purging)	50.00	0.25
Energy[a]	8.47	1.86
Total	-	2.55
**Operation/consumables (Glucose as substrate; photocatalyst recycle)[b]**		
$Al:SrTiO_3$	1.82	0.020
Co-SSP	2.84	0.67
Glucose	49.54	0.054
H_2O	25.00	0.028
N_2 (for reactor purging)	50.00	0.25
Energy[a]	8.47	1.86
Total	-	2.88

[a] Energy consumed during PC synthesis. Measured using an energy meter (Supplementary Figure 61).

[b] Operation/consumables costs calculated based on cumulative data from experiments performed on 28 September and 4 October 2024 with photocatalyst recycling and co-catalyst re-deposition.

Supplementary Table 33 | Cost of H_2 under several conditions. Data obtained from 1 m^2 demonstrations.

Condition	H_2 (mmol)	H_2 price (£ $mmol^{-1}$)
Cellulose[a]	1.51	7.44
Glucose[b]	5.24	1.71
Glucose; photocatalyst reuse[c]	9.96[d]	0.93

[a] Experiment performed on 30 August 2024.
[b] Experiment performed on 28 September 2024.
[c] Experiment performed on 04 October 2024.
[d] Sum of H_2 produced from the two experiments performed on 28 September and 04 October 2024. The Al:$SrTiO_3$ panels were reused in the second experiment with Co-SSP redeposited.

Supplementary Table 34 | Parameters used in "pessimistic", "base" and "optimistic" cases in the sensitivity analysis of the 1 m^2-scale system. The base case parameters are the real conditions in which the 1 m^2 demonstration were performed (experiments performed on 28 September and 4 October 2024 with photocatalyst recycling and co-catalyst re-deposition).

Variable	Unit	Pessimistic case	Base case	Optimistic case
Photocatalyst reuse	days	1	2	10
Light intensity[a]	suns	0.1	0.56[b]	2 (concentrated)
Daylight hours	hours	4	6	12
Reactor lifetime	days	50	100	200
Substrate source	-	Cellulose	Glucose	Wastewater[c]

[a] In the pessimistic case, H_2 production was interpolated from small-scale experiments performed under various light intensities. In the optimistic case, H_2 production beyond light intensities of 1 sun was assumed to scale with the square root of light intensity.[85]
[b] Average measured light intensity over the duration of the 1 m^2 demonstration.
[c] Such as sugar-rich effluent from the sugar industry.[86]

Supplementary Table 35 | Results of the sensitivity analysis depicted in Figure 5f. Calculated based on the parameters described in Supplementary Table 34.

Variable	H_2 price (£ $mmol^{-1}$)		
	Pessimistic case	Base case	Optimistic case
Catalyst reuse	1.71	0.93	0.24
Light intensity	5.23	0.93	0.37
Daylight hours	1.40	0.93	0.47
Reactor lifetime	1.58	0.93	0.61
Substrate source	3.94	0.93	0.93

Supplementary Table 36 | Band position of Al:SrTiO$_3$ and oxidation or reduction potential of relevant chemical reactions. The values are tabulated with respect to RHE at pH 7. N/A, not applicable.

Light absorber	Valence band maximum (V_{RHE})	Conduction band minimum (V_{RHE})
Al:SrTiO$_3$	+2.15	−1.03
Chemical reaction	Oxidation potential (V_{RHE})	Reduction potential (V_{RHE})
Glucose oxidation	−0.43	N/A
EG oxidation	−0.40	N/A
H_2 evolution	N/A	−0.41

Supplementary Table 37 | Loadings of Rh and Cr species on Al:SrTiO$_3$|RhCrO$_x$ before and after catalysis. The values were determined via ICP-OES before and after 22 h experiments.

Sample	Pre-catalysis loading (wt%)		Post-catalysis loading (wt%)	
	Rh	Cr	Rh	Cr
Al:SrTiO$_3$|RhCrO$_x$	0.10	0.11	0.09	0.06

Supplementary Table 38 | H_2 evolution comparison between Al:SrTiO$_3$|Co-SSP$_S$ and an Al:SrTiO$_3$|RhCrO$_x$ PC system with RhCrO$_x$ deposited using spin coating. While the benchmark RhCrO$_x$ co-catalyst is typically loaded using impregnation, spin coating was used in this case to study the performance of the co-catalysts using the same deposition technique. The photocatalytic experiments were performed under AM1.5G illumination for 22 h at room temperature. The data are presented as mean values ± SD for reactions performed in triplicate (n = 3).

PC sheet	Substrate	H_2 (μmol cm^{-2} h^{-1})
Al:SrTiO$_3$|Co-SSP$_S$	Pre-treated cellulose	0.21±0.03
	Pre-treated PET	0.07±0.01
Al:SrTiO$_3$|RhCrO$_x$ (spin coated)	Pre-treated cellulose	0.05±0.001
	Pre-treated PET	0.14±0.02

Supplementary Table 39 | H_2 evolution comparison between Al:SrTiO$_3$ PC sheets using Co-SSP, CoO, ZrO$_2$, and CoCl$_2$ as co-catalysts. The metal oxides were deposited onto the PC sheets with Nafion as a binder without annealing, while the CoCl$_2$ was deposited onto the PC sheet without a binder and without annealing.The photocatalytic experiments were performed under AM1.5G illumination for 22 h at room temperature. The data are presented as mean values ± SD for reactions performed in triplicate (n = 3).

Co-catalyst	H_2 (μmol cm^{-2} h^{-1})
Co-SSP	1.26±0.14
CoO	0.82±0.11
ZrO$_2$	0.01±0.01
CoCl$_2$	0.13±0.02

Supplementary Table 40 | Long term photocatalytic experiments with Al:SrTiO$_3$|Co-SSPs in reaction solution containing low concentrations (~0.1 mM) of Co^{2+} ions. The Co^{2+} ions in the reaction solution resulted from leached Co from a prior photocatalytic experiment also using an Al:SrTiO$_3$|Co-SSP PC sheet. For clarity, 0 h is denoted as the start of the second photocatalytic cycle where the used PC sheet was replaced with a fresh PC sheet. The photocatalytic experiments were performed under AM1.5G illumination for a total of 88 h at room temperature using glucose as the substrate. The data are presented as mean values ± SD for reactions performed in triplicate (n = 3).

Time (h)	H_2 (μmol cm^{-2} h^{-1})
−22	0
−20	3.23±0.41
−18	5.98±0.59
−16	8.13±0.52
0	15.08±0.32
0	0
2	2.30±0.48
4	4.01±0.77
6	6.09±0.93
22	16.0±1.89
28	18.6±2.23
44	26.1±3.84
66	33.5±5.42

Supplementary Table 41 | Long term photocatalytic experiments with Al:SrTiO$_3$|Co-SSPs, Al:SrTiO$_3$|Co-SSPs with a Nafion binder, and Al:SrTiO$_3$|Co-SSPs with annealing. The photocatalytic experiments were performed under AM1.5G illumination for 66 h at room temperature using glucose as the substrate. The data are presented as mean values ± SD for reactions performed in triplicate (n = 3).

Time (h)	H_2 (μmol cm^{-2} h^{-1})		
	Al:SrTiO$_3$|Co-SSPs	Al:SrTiO$_3$|Co-SSPs with binder	Al:SrTiO$_3$|Co-SSPs with annealing
0	0	0	0
2	3.50±0.65	4.80±0.47	2.85±0.45
4	6.73±0.90	7.17±0.60	5.22±0.66
6	9.89±2.08	9.37±0.70	6.87±0.84
22	15.72±0.75	16.87±1.90	15.10±0.36
28	16.50±0.65	18.02±2.27	16.72±0.28
44	17.61±1.34	19.52±3.63	22.33±1.73
66	17.44±1.46	20.21±4.41	26.73±2.23

Supplementary Table 42 | Loadings of Co and Zr species on Al:$SrTiO_3$|Co-SSPs when photocatalytic experiments were conducted in the presence of low concentrations of Co^{2+} and Zr^{4+} ions or only Zr^{4+} ions in the reaction solution. The Co^{2+} ions in the reaction solution resulted from leached Co from a prior photocatalytic experiment also using an Al:$SrTiO_3$|Co-SSP PC sheet. The values were determined via ICP-OES after the 66 h long-term experiments.

Ions present	Element (wt%)	
	Co	Zr
Co^{2+} and Zr^{4+}	0.86	0.34
Zr^{4+} only	0.09	0.33

Supplementary Table 43 | Long term photocatalytic experiments with Al:$SrTiO_3$|Co-SSPs in reaction solution containing 0.1 mM Zr^{4+} ions. The photocatalytic experiments were performed under AM1.5G illumination for a total of 66 h at room temperature using glucose as the substrate. The data are presented as mean values ± SD for reactions performed in triplicate (n = 3).

Time (h)	H_2 (μmol cm^{-2} h^{-1})
0	0
2	2.39±0.45
4	4.51±0.61
6	6.29±0.51
22	13.14±0.13
28	14.81±0.29
44	18.32±1.10
66	21.70±2.45

Supplementary Table 44 | Loadings of Co, Zr, Al, Sr and Ti species on Al:$SrTiO_3$|Co-SSPs without the use of binder and annealing, with a Nafion binder, and with annealing. The values were determined via ICP-OES after the 66 h long-term experiments.

Sample	Element (wt%)				
	Co	Zr	Al	Sr	Ti
Al:$SrTiO_3$|Co-SSPs	0.01	0.10	0.28	48	26
Al:$SrTiO_3$|Co-SSPs with binder	0.19	0.29	0.28	48	26
Al:$SrTiO_3$|Co-SSPs with annealing	0.28	0.80	0.27	48	26

Supplementary Table 45 | H_2 evolution of Al:$SrTiO_3$|Co-SSPs with pre-treated cellulose as well as high and low concentrations of glucose as substrate. The photocatalytic experiments were performed under AM1.5G illumination for 22 h at room temperature. The data are presented as mean values ± SD for reactions performed in triplicate (n = 3).

Substrate	H_2 (μmol cm^{-2} h^{-1})
0.1 M glucose	0.67±0.09
0.011 M glucose	0.57±0.07
Pre-treated cellulose	0.21±0.03

Supplementary Table 46 | H_2 evolution from the 12 h medium-scale outdoor experiments using Al:$SrTiO_3$|Co-SSP_M. Glucose was used as the substrate.

Time	H_2 (μmol cm^{-2})
9:00	0
12:00	30.1
15:00	78.6
18:00	90.4
21:00	96.7

Supplementary Table 47 | H_2 evolution from the overnight medium-scale outdoor experiments using Al:$SrTiO_3$|Co-SSP_M with the PC sheet left in the reaction solution overnight. Glucose was used as the substrate.

Time	H_2 (μmol cm^{-2})
9:00	0
12:00	23.1
15:00	69.8
NIGHT	
9:00	0
12:00	1.8
15:00	3.2

Supplementary Table 48 | H_2 evolution from the overnight medium-scale outdoor experiments using Al:$SrTiO_3$|Co-SSP_M with the PC sheet stored outside the reaction solution (i.e. in dry conditions) for 10 days in between each outdoor experiment. Glucose was used as the substrate.

Time	H_2 (μmol cm^{-2})
9:00	0
12:00	17.7
15:00	65.6
10 NIGHTS	
9:00	0
12:00	39.2
15:00	54.9

Supplementary Table 49 | Performance comparison between Al:SrTiO$_3$|Co-SSP$_S$ and Al:SrTiO$_3$|Co-SSP$_M$ PC sheets under standard conditions as well as with solution depth and temperature fixed at 1.2 cm and 40 °C, respectively. Standard conditions refer to a solution depth of ~1.4 and ~0.9 cm for the small (1 cm^2) and medium (20.25 cm^2) scale experiments, respectively, at room temperature. The photocatalytic experiments were performed in 0.1 M glucose solution under AM1.5G illumination for 22 h and 6 h for the small and medium scale experiments, respectively. The data are presented as mean values ± SD for reactions performed in triplicate (n = 2).

PC sheet	Conditions	H_2 (μmol cm^{-2} h^{-1})
Al:SrTiO$_3$|Co-SSP$_S$	Standard	0.67±0.09
	Solution depth 1.2 cm; 40 °C	0.68±0.02
Al:SrTiO$_3$|Co-SSP$_M$	Standard	0.32±0.05
	Solution depth 1.2 cm; 40 °C	0.35±0.05

Supplementary References

1. Lam, E. *et al.* Comproportionation of CO_2 and Cellulose to Formate Using a Floating Semiconductor-Enzyme Photoreforming Catalyst. *Angew. Chemie Int. Ed.* **62**, e202215894 (2023).

2. Bhattacharjee, S. *et al.* Chemoenzymatic Photoreforming: A Sustainable Approach for Solar Fuel Generation from Plastic Feedstocks. *J. Am. Chem. Soc.* **145**, 20355–20364 (2023).

3. Takata, T. *et al.* Photocatalytic water splitting with a quantum efficiency of almost unity. *Nature* **581**, (2020).

4. Nishiyama, H. *et al.* Photocatalytic solar hydrogen production from water on a 100-m^2 scale. *Nature* **598**, 304–307 (2021).

5. Goto, Y. *et al.* A Particulate Photocatalyst Water-Splitting Panel for Large-Scale Solar Hydrogen Generation A Particulate Photocatalyst Water-Splitting Panel for Large-Scale Solar Hydrogen Generation. *Joule* **2**, 509–520 (2018).

6. Kanan, M. W. & Nocera, D. G. In Situ Formation of an Oxygen-Evolving Catalyst in Neutral Water Containing Phosphate and Co^{2+}. *Science (80-.).* **321**, 1072–1075 (2008).

7. Simondson, D. *et al.* Stable Acidic Water Oxidation with a Cobalt–Iron–Lead Oxide Catalyst Operating via a Cobalt-Selective Self-Healing Mechanism. *Angew. Chemie Int. Ed.* **60**, 15821–15826 (2021).

8. Gupta, R. & Lee, Y. Y. Mechanism of cellulase reaction on pure cellulosic substrates. *Biotechnol. Bioeng.* **102**, 1570–1581 (2009).

9. Ren, P. *et al.* Stepwise photoassisted decomposition of carbohydrates to H_2. *Joule* **7**, 333–349 (2023).

10. Morales-delaRosa, S., Campos-Martin, J. M. & Fierro, J. L. G. Optimization of the process of chemical hydrolysis of cellulose to glucose. *Cellulose* **21**, 2397–2407 (2014).

11. Pang, J., Wang, A., Zheng, M. & Zhang, T. Hydrolysis of cellulose into glucose over carbons sulfonated at elevated temperatures. *Chem. Commun.* **46**, 6935 (2010).

12. Zhang, L., Wang, W., Zeng, S., Su, Y. & Hao, H. Enhanced H_2 evolution from photocatalytic cellulose conversion based on graphitic carbon layers on TiO_2/NiO_x. *Green Chem.* **20**, 3008–3013 (2018).

13. Zhao, H. *et al.* Mechanistic understanding of cellulose β-1,4-glycosidic cleavage via photocatalysis. *Appl. Catal. B Environ.* **302**, 120872 (2022).

14. Zhao, H. *et al.* Confined synthesis of $BiVO_4$ nanodot and ZnO cluster co-decorated 3DOM TiO_2 for formic acid production from the xylan-based hemicellulose photorefinery. *Green Chem.* **23**, 8124–8130 (2021).

15. Machado, A. E. H. *et al.* Photocatalytic degradation of lignin and lignin models, using titanium dioxide: the role of the hydroxyl radical. *Chemosphere* **40**, 115–124 (2000).

16. Chong, R. *et al.* Selective conversion of aqueous glucose to value-added sugar aldose on TiO_2-based photocatalysts. *J. Catal.* **314**, 101–108 (2014).

17. Kupiainen, L., Ahola, J. & Tanskanen, J. Distinct Effect of Formic and Sulfuric Acids on Cellulose Hydrolysis at High Temperature. *Ind. Eng. Chem. Res.* **51**, 3295–3300 (2012).

18. Iranmahboob, J., Nadim, F. & Monemi, S. Optimizing acid-hydrolysis: a critical step for production of ethanol from mixed wood chips. *Biomass and Bioenergy* **22**, 401–404 (2002).

19. Chu, C.-Y., Wu, S.-Y., Tsai, C.-Y. & Lin, C.-Y. Kinetics of cotton cellulose hydrolysis using concentrated acid and fermentative hydrogen production from hydrolysate. *Int. J. Hydrogen Energy* **36**, 8743–8750 (2011).

20. Shahbazi, A. & Zhang, B. Dilute and concentrated acid hydrolysis of lignocellulosic biomass. in *Bioalcohol Production* 143–158 (Elsevier, 2010). doi:10.1533/9781845699611.2.143.

21. Lam, E. *et al.* Comproportionation of CO_2 and Cellulose to Formate Using a Floating Semiconductor-Enzyme Photoreforming Catalyst. *Angew. Chemie Int. Ed.* **62**, e202215894 (2023).

22. Kuhad, R. C. *et al.* Revisiting cellulase production and redefining current strategies based on major challenges. *Renew. Sustain. Energy Rev.* **55**, 249–272 (2016).

23. Lee, S.-M. & Koo, Y.-M. Pilot-scale Production of Cellulase Using Trichoderma reesei Rut C-30 in Fed-Batch Mode. *J. Microbiol. Biotechnol.* **11**, 229–233 (2001).

24. Lin, X., Xue, X. & Du, J. Electrochemical glucose-to-formic acid conversion coupled with alkaline hydrogen production over nanostructured $CuCo_2O_4$ catalysts. *J. Mater. Chem. A* **12**, 32095–32103 (2024).

25. Basu, D. & Basu, S. A study on direct glucose and fructose alkaline fuel cell. *Electrochim. Acta* **55**, 5775–5779 (2010).

26. Kulkarni, A. A. & Joshi, J. B. Bubble Formation and Bubble Rise Velocity in Gas−Liquid Systems: A Review. *Ind. Eng. Chem. Res.* **44**, 5873–5931 (2005).

27. Chirife, J. & Buera, M. P. A simple model for predicting the viscosity of sugar and oligosaccharide solutions. *J. Food Eng.* **33**, 221–226 (1997).

28. Cespi, D. *et al.* LCA of precious metals recovery: Modelling the secondary supply of gold, silver, platinum, palladium and rhodium from an integrated refining plant. *Clean. Environ. Syst.* **19**, 100310 (2025).

29. Veziroglu, S. *et al.* Photocatalytic deposition of noble metals on 0D, 1D, and 2D TiO_2 structures: a review. *Nanoscale Adv.* **6**, 6096–6108 (2024).

30. Faverge, T. *et al.* In Situ Investigation of d -Glucose Oxidation into Value-Added Products on Au, Pt, and Pd under Alkaline Conditions: A Comparative Study. *ACS Catal.* **13**, 2657–2669 (2023).

31. Yan, L. *et al.* Efficient and poison-tolerant Pd Au/C binary electrocatalysts for glucose electrooxidation in alkaline medium. *Appl. Catal. B Environ.* **150–151**, 268–274 (2014).

32. Sanwald, K. E. *et al.* Kinetic Coupling of Water Splitting and Photoreforming on SrTiO3-Based Photocatalysts. *ACS Catal.* **8**, 2902–2913 (2018).

33. Pinaud, B. A. *et al.* Technical and economic feasibility of centralized facilities for solar hydrogen production via photocatalysis and photoelectrochemistry. *Energy Environ. Sci.* **6**, 1983 (2013).

34. Uekert, T., Pichler, C. M., Schubert, T. & Reisner, E. Solar-driven reforming of solid waste for a sustainable future. *Nat. Sustain.* **4**, 383–391 (2021).

35. Toe, C. Y., Pan, J., Scott, J. & Amal, R. Identifying Key Design Criteria for Large-Scale Photocatalytic Hydrogen Generation from Engineering and Economic Perspectives. *ACS ES&T Eng.* **2**, 1130–1143 (2022).

36. Liu, J. *et al.* Metal-free efficient photocatalyst for stable visible water splitting via a two-electron pathway. *Science (80-.).* **347**, 970–974 (2015).

37. Kibria, M. G. *et al.* Visible light-driven efficient overall water splitting using p-type metal-nitride nanowire arrays. *Nat. Commun.* **6**, 6797 (2015).

38. Liao, L. *et al.* Efficient solar water-splitting using a nanocrystalline CoO photocatalyst. *Nat. Nanotechnol.* **9**, 69–73 (2014).

39. Fan, J. *et al.* Microwave-enhanced formation of glucose from cellulosic waste. *Chem. Eng. Process. Process Intensif.* **71**, 37–42 (2013).

40. Dussan, K. J., Silva, D. D. V., Moraes, E. J. C., Arruda, P. V. & Felipe, M. G. A. Dilute-acid Hydrolysis of Cellulose to Glucose from Sugarcane Bagasse. *Chem. Eng. Trans.* **38**, 433–438 (2014).

41. Uekert, T., Bajada, M. A., Schubert, T., Pichler, C. M. & Reisner, E. Scalable Photocatalyst Panels for Photoreforming of Plastic, Biomass and Mixed Waste in Flow. *ChemSusChem* **14**, 4190–4197 (2021).

42. Uekert, T., Kasap, H. & Reisner, E. Photoreforming of Nonrecyclable Plastic Waste over a Carbon Nitride/Nickel Phosphide Catalyst. *J. Am. Chem. Soc.* **141**, 15201–15210 (2019).

43. Puga, A. V. Photocatalytic production of hydrogen from biomass-derived feedstocks. *Coord. Chem. Rev.* **315**, 1–66 (2016).

44. Schröder, M. *et al.* Hydrogen Evolution Reaction in a Large-Scale Reactor using a Carbon Nitride Photocatalyst under Natural Sunlight Irradiation. *Energy Technol.* **3**, 1014–1017 (2015).

45. Battula, V. R. *et al.* Binder-Free Carbon Nitride Panels for Continuous-Flow Photocatalysis. *ACS Catal.* **14**, 11666–11674 (2024).

46. Hu, X., Zhan, Z., Zhang, J., Hussain, I. & Tan, B. Immobilized covalent triazine frameworks films as effective photocatalysts for hydrogen evolution reaction. *Nat. Commun.* **12**, 6596 (2021).

47. Wang, Q. *et al.* Scalable water splitting on particulate photocatalyst sheets with a solar-to-hydrogen energy conversion efficiency exceeding 1%. *Nat. Mater.* **15**, 611–615 (2016).

48. Wang, Q. *et al.* Particulate Photocatalyst Sheets Based on Carbon Conductor Layer for Efficient Z-Scheme Pure-Water Splitting at Ambient Pressure. *J. Am. Chem. Soc.* **139**, 1675–1683 (2017).

49. Wang, Q. *et al.* Printable Photocatalyst Sheets Incorporating a Transparent Conductive Mediator for Z-Scheme Water Splitting. *Joule* **2**, 2667–2680 (2018).

50. Pan, Z. *et al.* Photocatalyst Sheets Composed of Particulate $LaMg_{1/3}Ta_{2/3}O_2N$ and Mo-Doped $BiVO_4$ for Z-Scheme Water Splitting under Visible Light. *ACS Catal.* **6**, 7188–7196 (2016).

51. Xiong, A. *et al.* Fabrication of photocatalyst panels and the factors determining their activity for water splitting. *Catal. Sci. Technol.* **4**, 325–328 (2014).

52. Pornrungroj, C. *et al.* Hybrid photothermal–photocatalyst sheets for solar-driven overall water splitting coupled to water purification. *Nat. Water* **1**, 952–960 (2023).

53. Chen, X. *et al.* Three-dimensional porous g-C_3N_4 for highly efficient photocatalytic overall water splitting. *Nano Energy* **59**, 644–650 (2019).

54. Chai, Z., Mattsson, A., Tesfamhret, Y., Österlund, L. & Zhu, J. Ni–Ag Nanostructure-Modified Graphitic Carbon Nitride for Enhanced Performance of Solar-Driven Hydrogen Production from Ethanol. *ACS Appl. Energy Mater.* **3**, 10131–10138 (2020).

55. Hong, J., Wang, Y., Wang, Y., Zhang, W. & Xu, R. Noble-Metal-Free NiS/C_3N_4 for Efficient Photocatalytic Hydrogen Evolution from Water. *ChemSusChem* **6**, 2263–2268 (2013).

56. Yang, L. *et al.* Surface Water Loading on Titanium Dioxide Modulates Photocatalytic Water Splitting. *Cell Reports Phys. Sci.* **1**, 100013 (2020).

57. Spanu, D. *et al.* Templated Dewetting–Alloying of NiCu Bilayers on TiO_2 Nanotubes Enables Efficient Noble-Metal-Free Photocatalytic H 2 Evolution. *ACS Catal.* **8**, 5298–5305 (2018).

58. Plastic Sheets Direct. 5 mm Clear Acrylic. https://www.plasticsheetsdirect.co.uk/product/5mm-clear-acrylic/

59. Rubber Co. Heavy duty rubber sheet. https://rubberco.co.uk/products/commercial-rubber-sheet-linear-meter

60. Aluminium Online. Aluminium Plain Sheet. https://www.aluminium-online.co.uk/product-category/sheet/aluminium-sheet/

61. KI Metals. Mild steel U channel. https://kimetals.co.uk/materials/mild-steel/mild-steel-channel/kim41734/

62. The Research Agency of the Forrestry Commission. *Timber Price Indices*. (2024).

63. Aluminium Warehouse. Aluminium rectangular tube. https://www.aluminiumwarehouse.co.uk/products/40-mm-x-20-mm-x-2-mm-aluminium-rectangular-tube

64. Metals4u. Mild steel tube. https://www.metals4u.co.uk/materials/mild-steel/mild-steel-tube/tube/2337-p

65. The Glass Warehouse. Frosted Satin Glass. https://www.theglasswarehouse.co.uk/frosted-satin-glass/

66. Adafruit Industries LLC. Toggle clamp - rubber tip. https://www.digikey.co.uk/en/products/detail/adafruit-industries-llc/2456/7244953

67. RS Pro. Straight tube-to-tube adaptor. https://uk.rs-online.com/web/p/pneumatic-fittings/9160886

68. Auto Silicone Hoses. PVC Unreinforced Tube. https://www.autosiliconehoses.com/pvc-tube-6mm-1-4-inch-metre-clear.html

69. Shaner, M. R., Atwater, H. A., Lewis, N. S. & McFarland, E. W. A comparative technoeconomic analysis of renewable hydrogen production using solar energy. *Energy Environ. Sci.* **9**, 2354–2371 (2016).

70. Natural Micron Chem Tech Co. Ltd. Cellulase. https://www.alibaba.com/product-detail/High-Quality-Cellulase-CAS-9012-54_11000006785562.html

71. Foodchem International Corporation. Microcrystalline cellulose. https://www.alibaba.com/product-detail/25KG-BAG-Wholesale-Food-Grade-Microcrystalline_60508430959.html

72. Hebei Chuanghai Biotechnology Co. Ltd. D(+)-Glucose. https://www.alibaba.com/product-detail/Food-grade-99-D-Glucose-CAS_1601185005126.html

73. Qingdao Hot Chemicals Co. Ltd. Sodium bicarbonate, 99%. https://www.alibaba.com/product-detail/Factory-Supply-Sodium-Bicarbonate-99-min_1600388020001.html

74. Cambridge Water. Metered water charges 2024-2025. https://www.cambridge-water.co.uk/household/my-bills-and-payments/my-bill-explained/metered-charges-explained/metered-charges

75. BOC Online UK. Nitrogen (Oxygen Free) 230bar Cylinder. https://www.boconline.co.uk/shop/en/uk/nitrogen-oxygen-free-230-bar-cylinder

76. Ofgem. Energy price cap. https://www.ofgem.gov.uk/energy-price-cap

77. Luoyang Tongrun Info Technology Co. Ltd. Al_2O_3 powder, 50nm. https://www.alibaba.com/product-detail/99-999-High-Purity-Gamma-Alumina_1601242621394.html

78. Jinan Yudong Trading Co. Ltd. $SrTiO_3$ powder nanoparticles. https://www.alibaba.com/product-detail/Strontium-Titanate-Powder-Nanoparticles-Srtio3-Nanopowder_1601169857614.html

79. Jinan Jinbang Chemical Co. Ltd. $SrCl_2.6H_2O$, 99%.

80. Guangzhou Yaoguang Technology Co. Ltd. Zirconium n-propoxide.

81. Qingdao Jinyu Chemical Co. Ltd. Cobalt(II) acetylacetonate, 99%. https://www.alibaba.com/product-detail/professional-supplier-Cobalt-Acetylacetonate-Cobalt-II_1600939867071.html

82. ReAgent. Toluene. https://www.chemicals.co.uk/toluene.

83. Alliance Chemical. Hexane ACS reagent grade. https://alliancechemical.com/products/hexane-acs-reagent-grade

84. Dongying Eastchem Co. Ltd. Tetrahydrofuran, 99%. https://www.alibaba.com/product-detail/china-supplier-for-99-9-

chemical_1601112266777.html

85. Nosaka, Y. & Nosaka, A. Y. Langmuir–Hinshelwood and Light-Intensity Dependence Analyses of Photocatalytic Oxidation Rates by Two-Dimensional-Ladder Kinetic Simulation. *J. Phys. Chem. C* **122**, 28748–28756 (2018).

86. Nouhou Moussa, A. W., Sawadogo, B., Konate, Y., Sidibe, S. dit S. & Heran, M. Critical State of the Art of Sugarcane Industry Wastewater Treatment Technologies and Perspectives for Sustainability. *Membranes (Basel).* **13**, 709 (2023).

End of Supplementary Information
