# Supplementary figures and images for: Photoreforming of solid waste on 1 m2 scale using single-source precursor-derived co-catalyst films
Source: Nat Chem Eng. 2026 Jun 24;3(6):351–62. doi: 10.1038/s44286-026-00406-y (PMC13293892; doi:10.1038/s44286-026-00406-y)

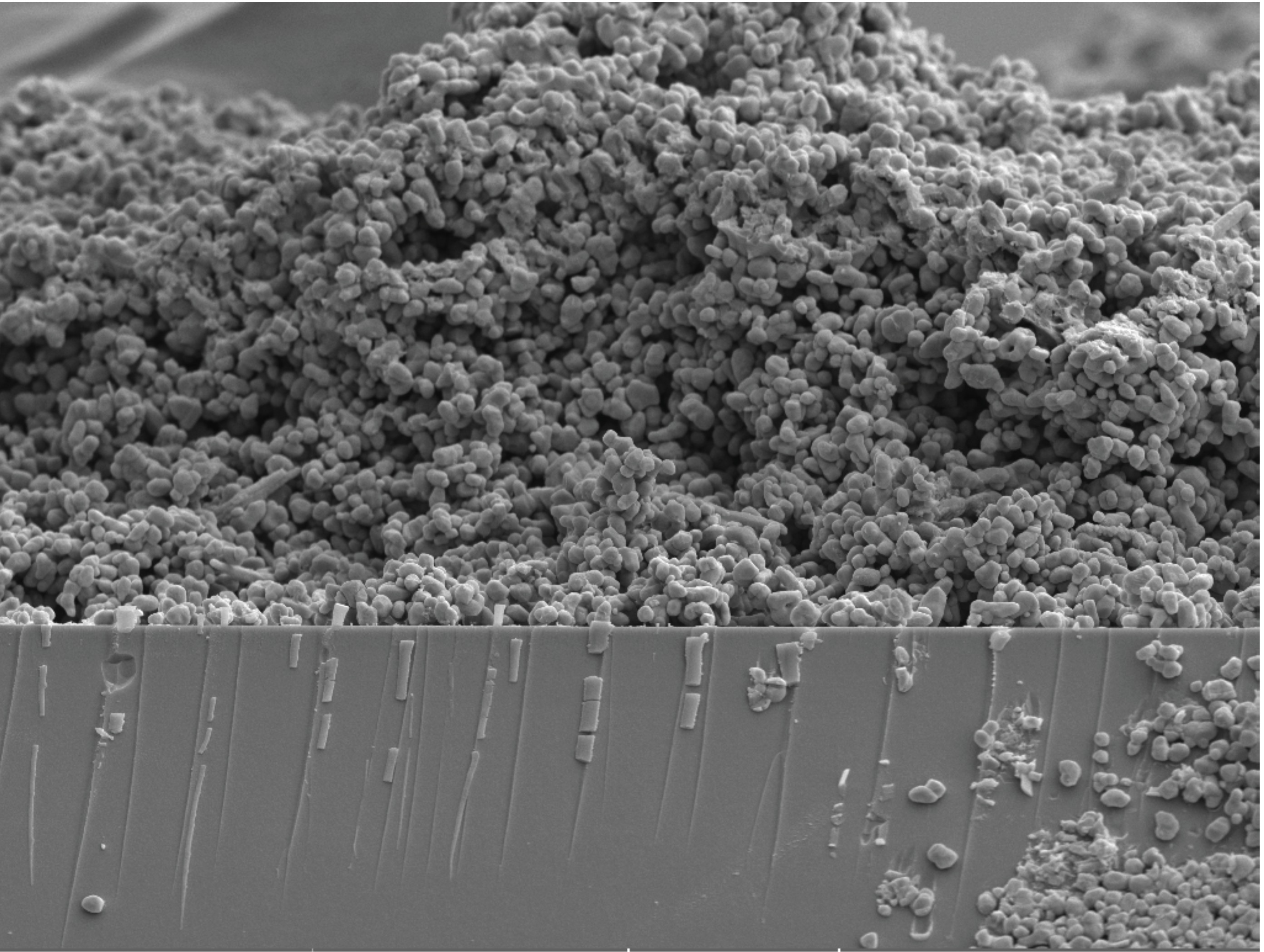

Supplement: Supplementary file 4 — Source data for Figs. 1–5. [file 44286_2026_406_MOESM4_ESM.zip › Source data main figures/Figure 4/4b.jpg]

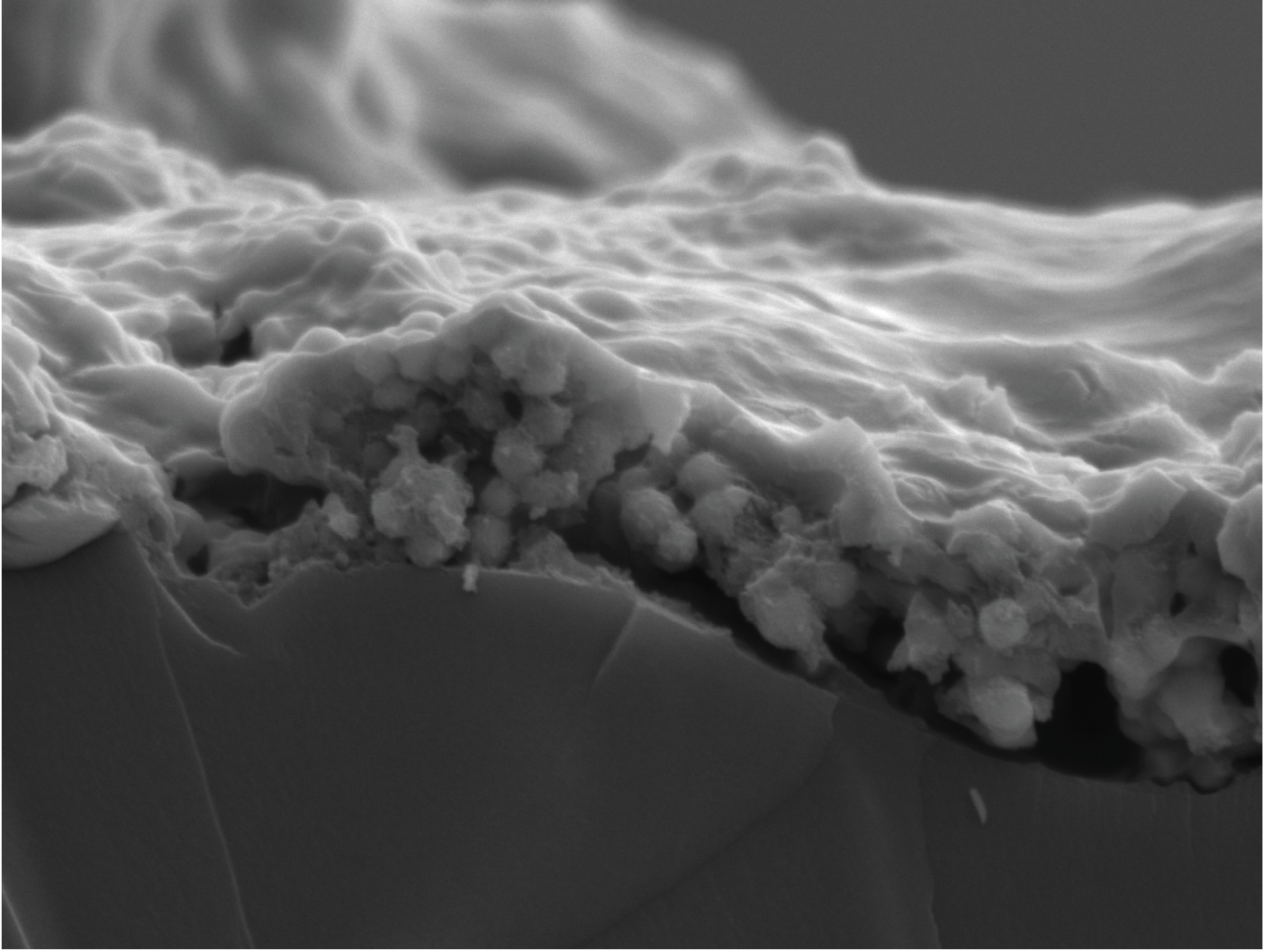

Supplement: Supplementary file 4 — Source data for Figs. 1–5. [file 44286_2026_406_MOESM4_ESM.zip › Source data main figures/Figure 4/4c.jpg]

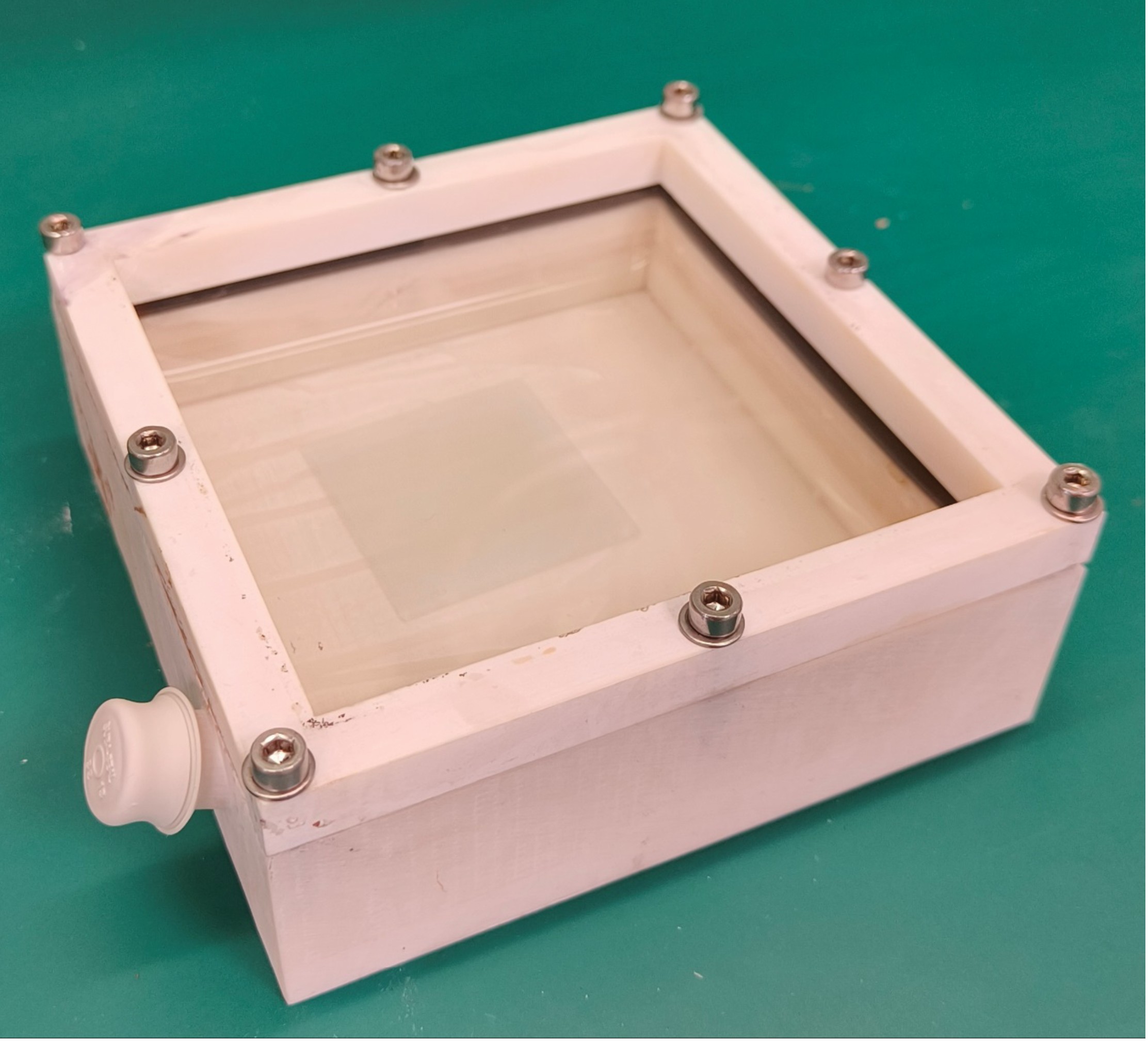

Supplement: Supplementary file 4 — Source data for Figs. 1–5. [file 44286_2026_406_MOESM4_ESM.zip › Source data main figures/Figure 4/4a.jpg]

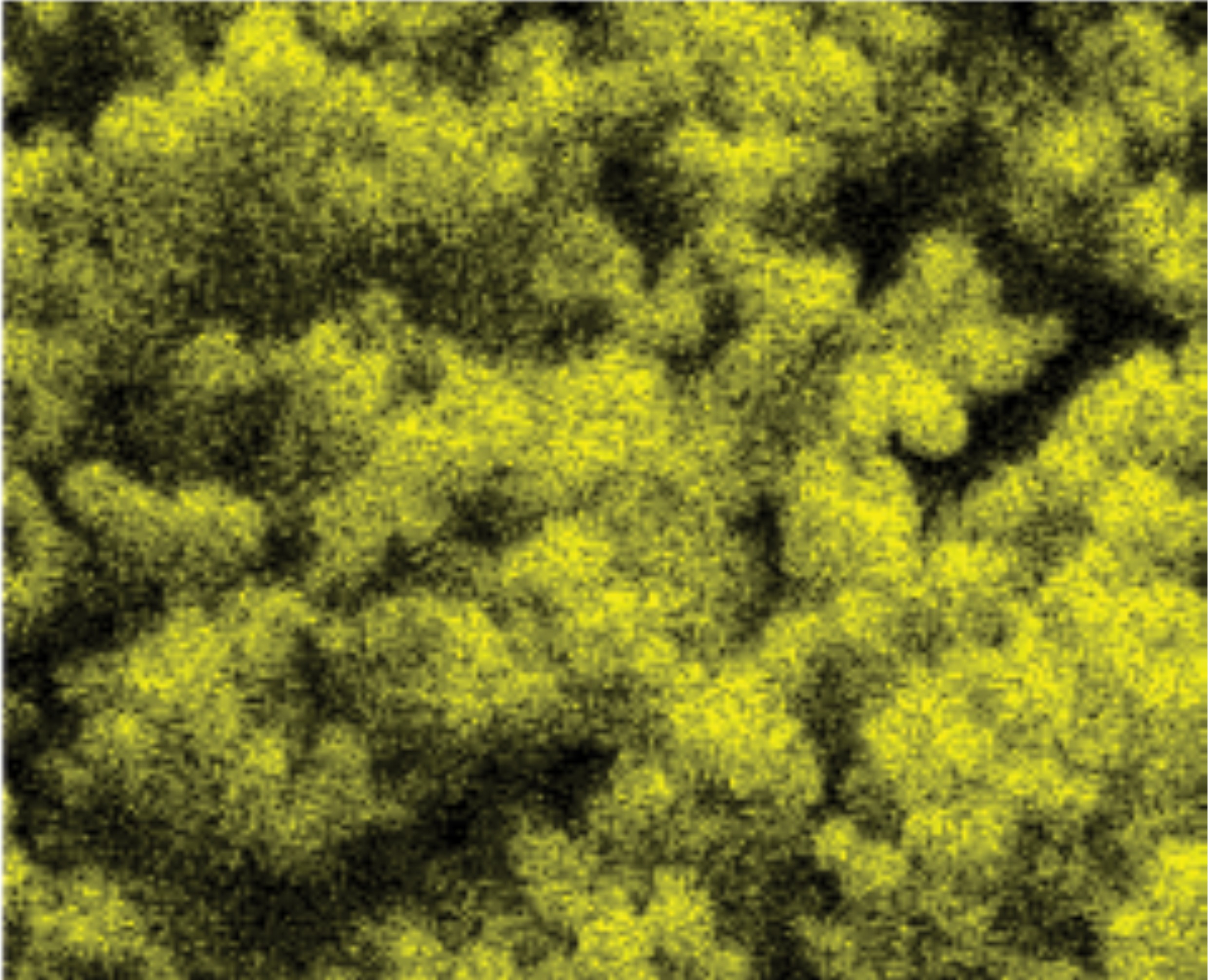

Supplement: Supplementary file 4 — Source data for Figs. 1–5. [file 44286_2026_406_MOESM4_ESM.zip › Source data main figures/Figure 2/2h.jpg]

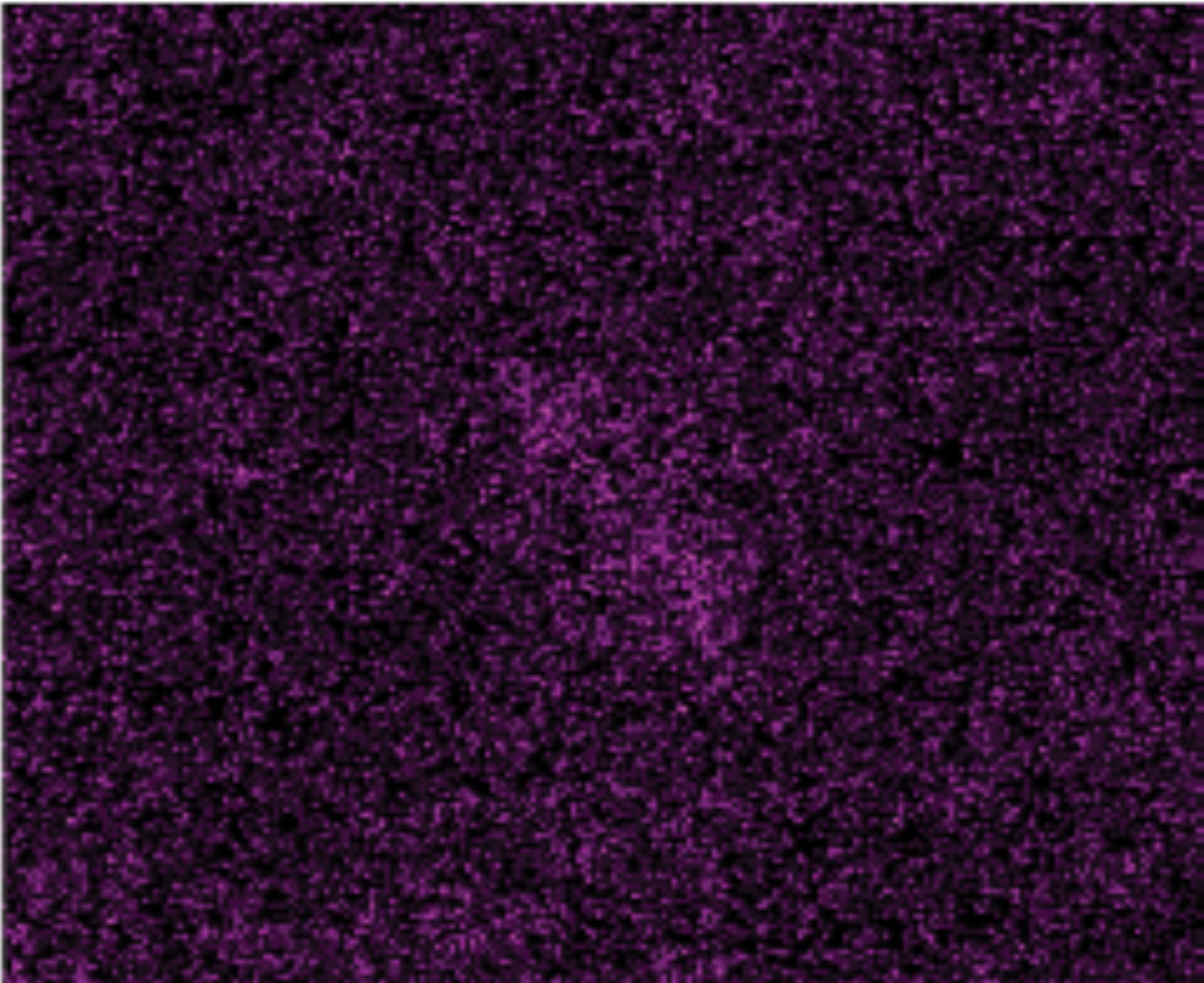

Supplement: Supplementary file 4 — Source data for Figs. 1–5. [file 44286_2026_406_MOESM4_ESM.zip › Source data main figures/Figure 2/2i.jpg]

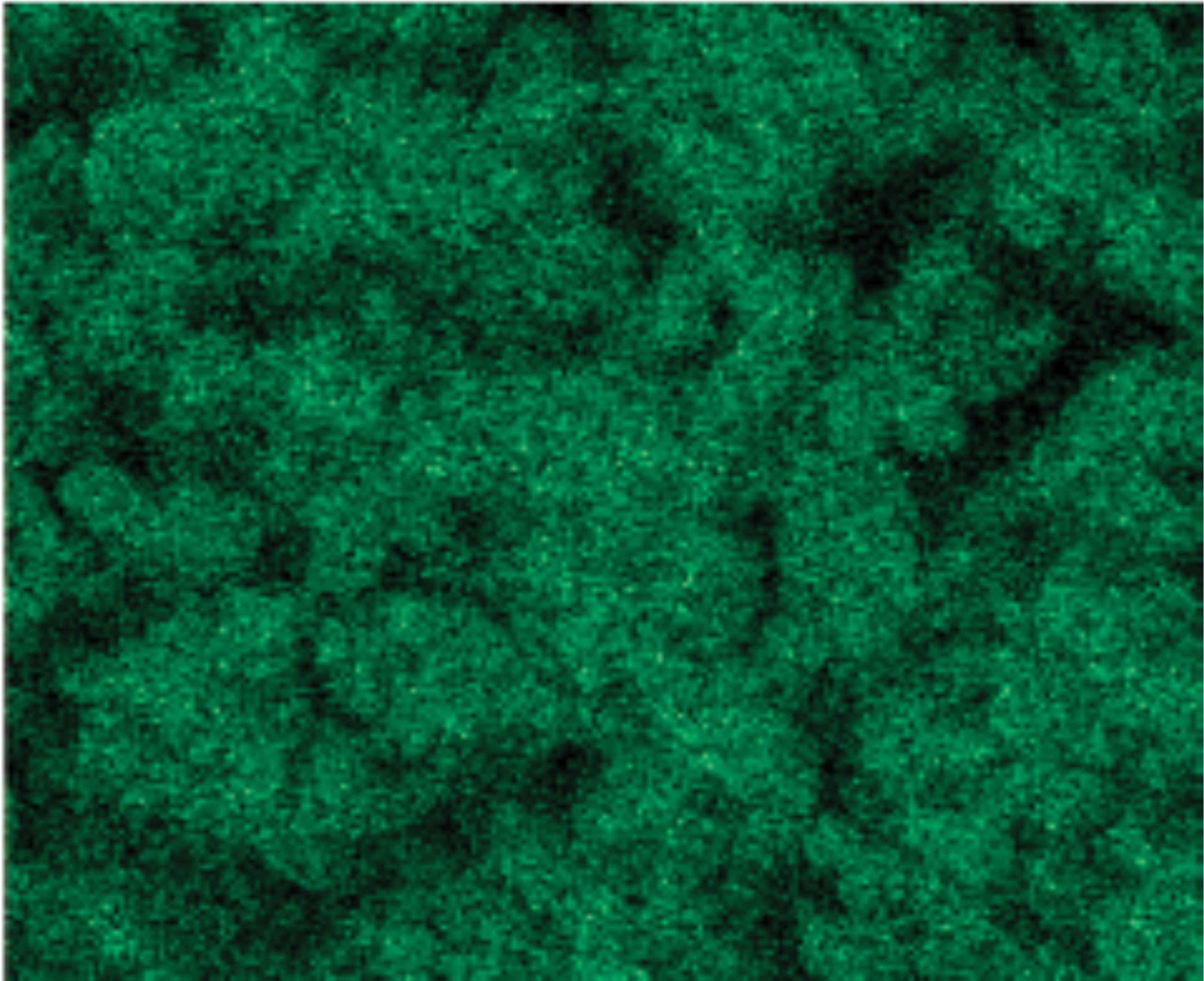

Supplement: Supplementary file 4 — Source data for Figs. 1–5. [file 44286_2026_406_MOESM4_ESM.zip › Source data main figures/Figure 2/2j.jpg]

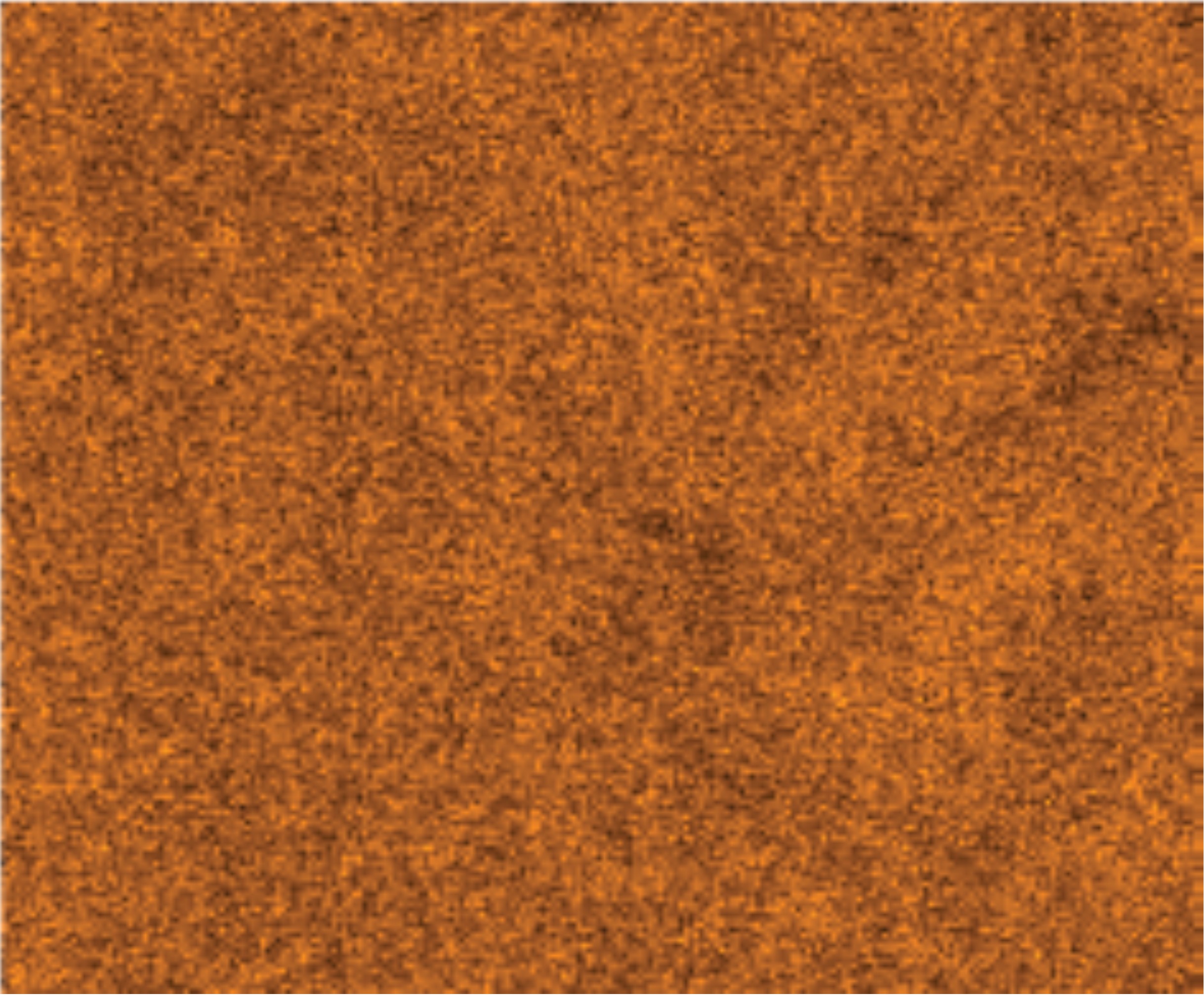

Supplement: Supplementary file 4 — Source data for Figs. 1–5. [file 44286_2026_406_MOESM4_ESM.zip › Source data main figures/Figure 2/2g.jpg]

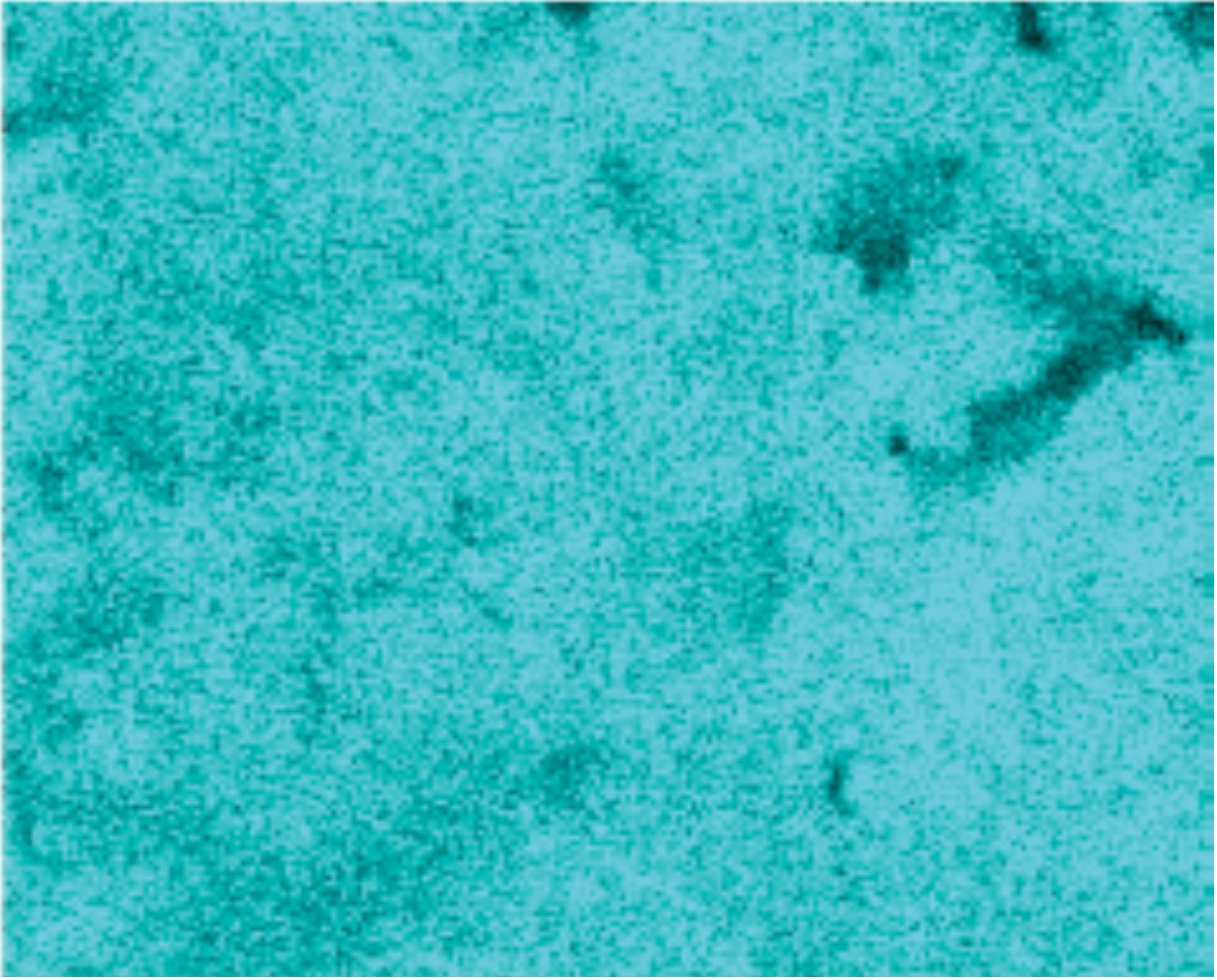

Supplement: Supplementary file 4 — Source data for Figs. 1–5. [file 44286_2026_406_MOESM4_ESM.zip › Source data main figures/Figure 2/2f.jpg]

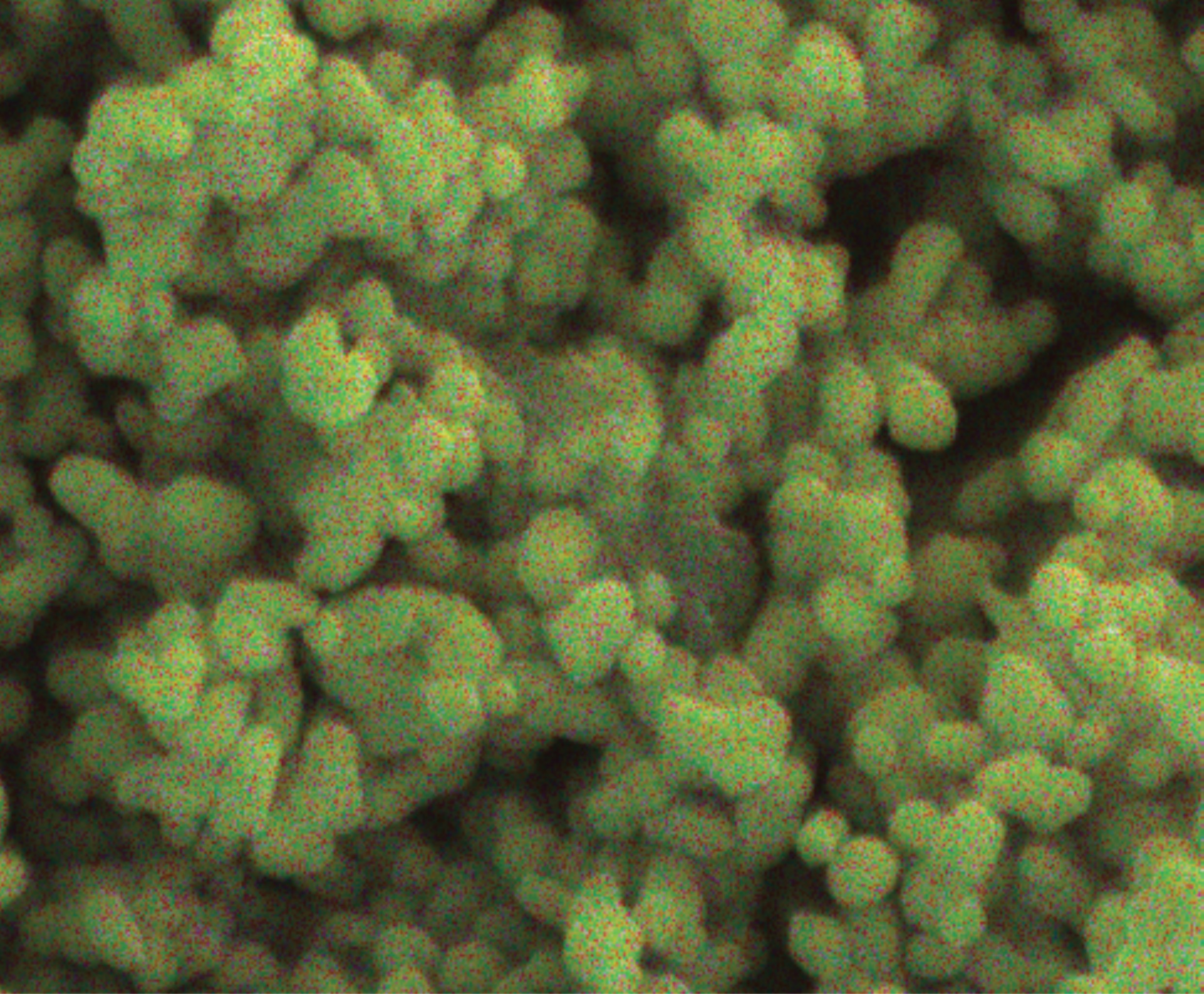

Supplement: Supplementary file 4 — Source data for Figs. 1–5. [file 44286_2026_406_MOESM4_ESM.zip › Source data main figures/Figure 2/2d.jpg]

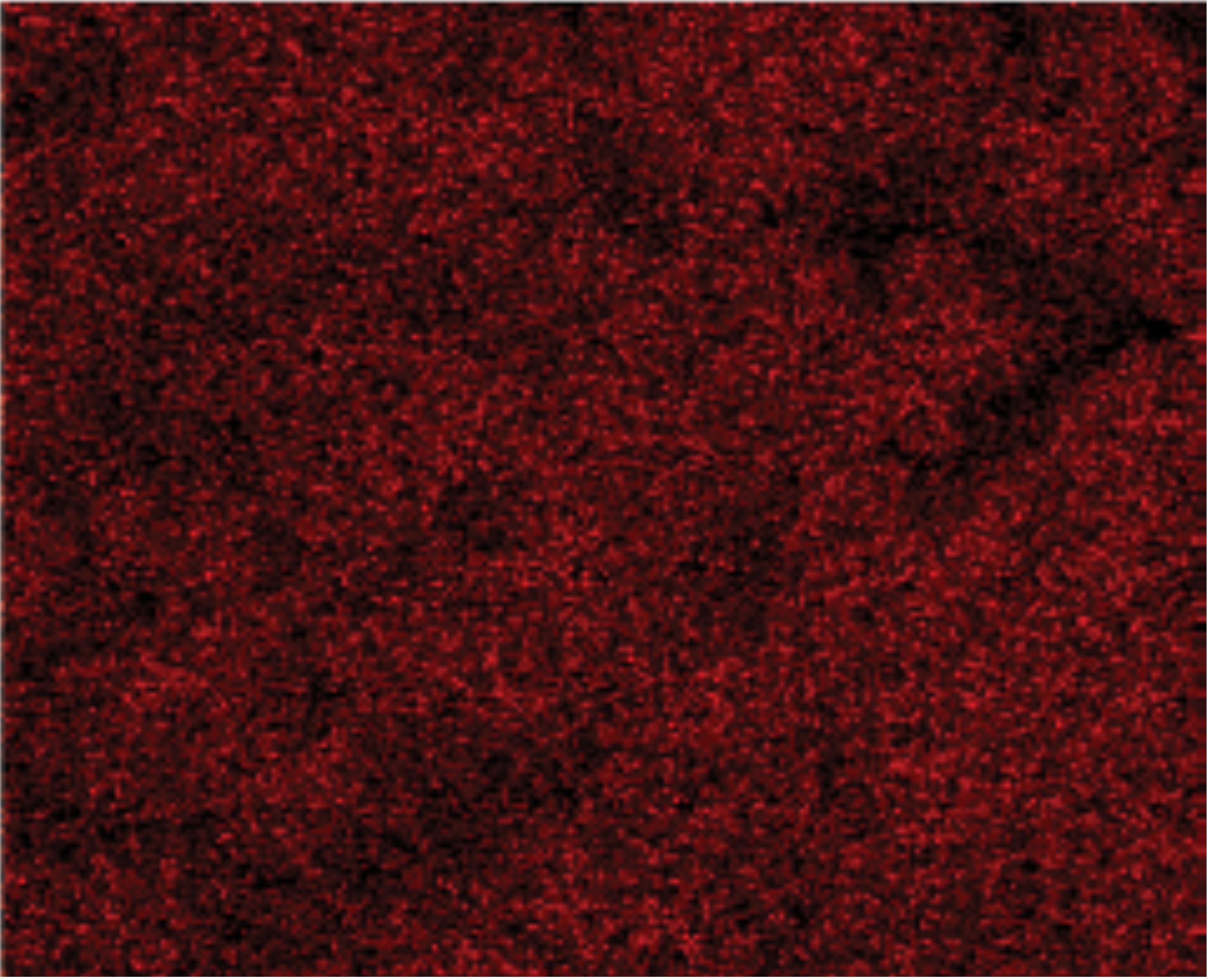

Supplement: Supplementary file 4 — Source data for Figs. 1–5. [file 44286_2026_406_MOESM4_ESM.zip › Source data main figures/Figure 2/2e.jpg]

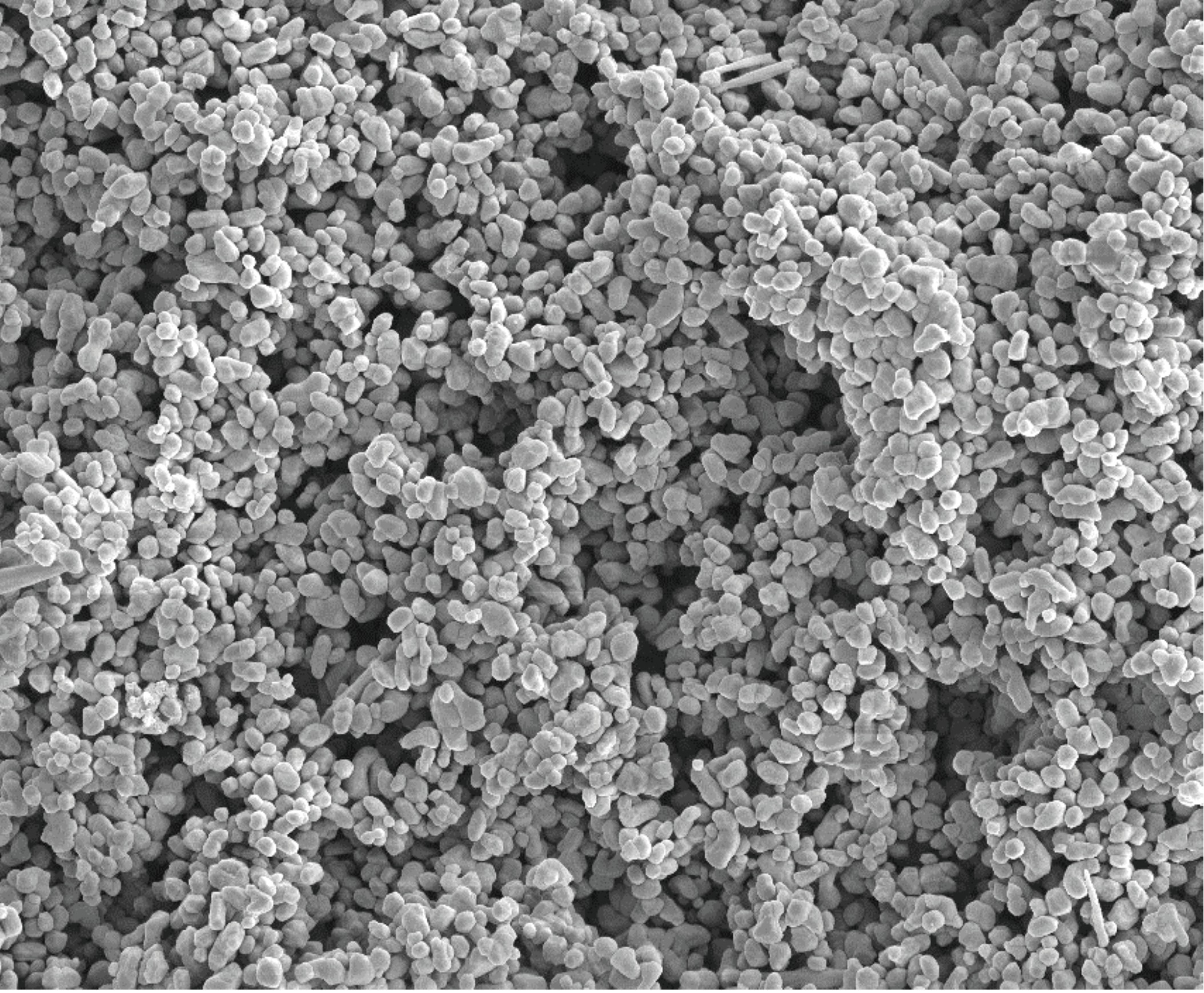

Supplement: Supplementary file 4 — Source data for Figs. 1–5. [file 44286_2026_406_MOESM4_ESM.zip › Source data main figures/Figure 2/2c.jpg]

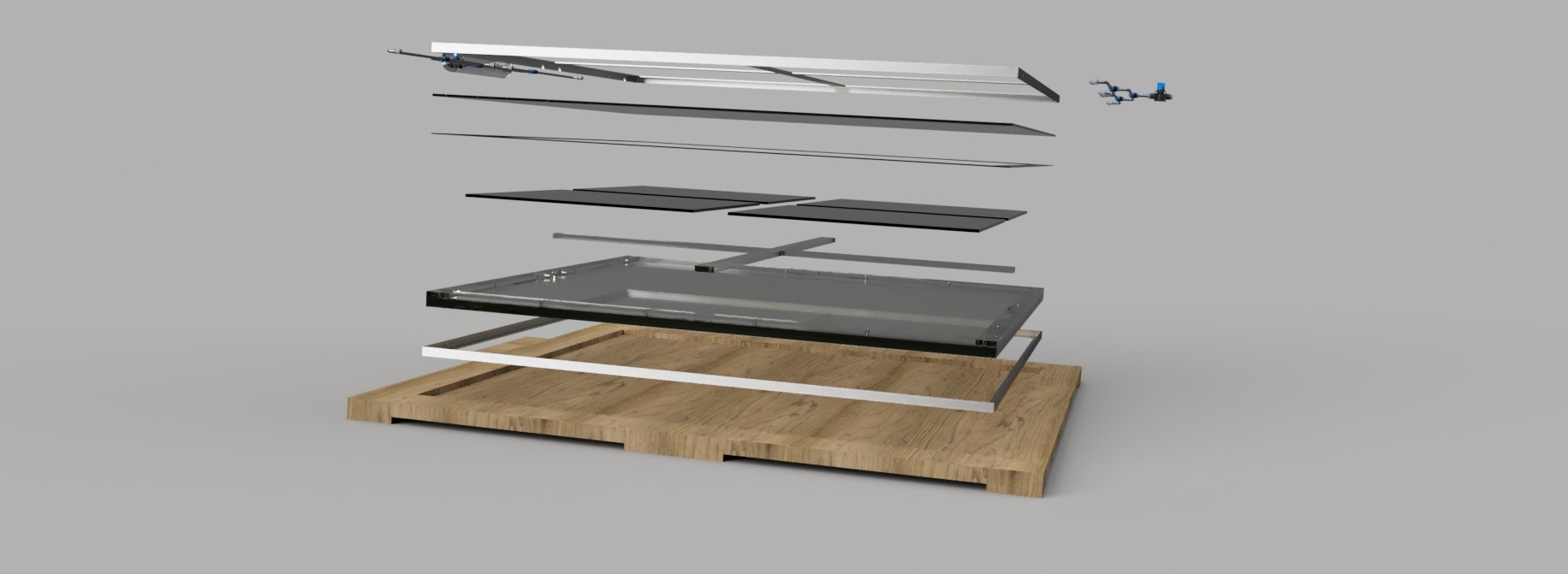

Supplement: Supplementary file 4 — Source data for Figs. 1–5. [file 44286_2026_406_MOESM4_ESM.zip › Source data main figures/Figure 5/3b.jpg]

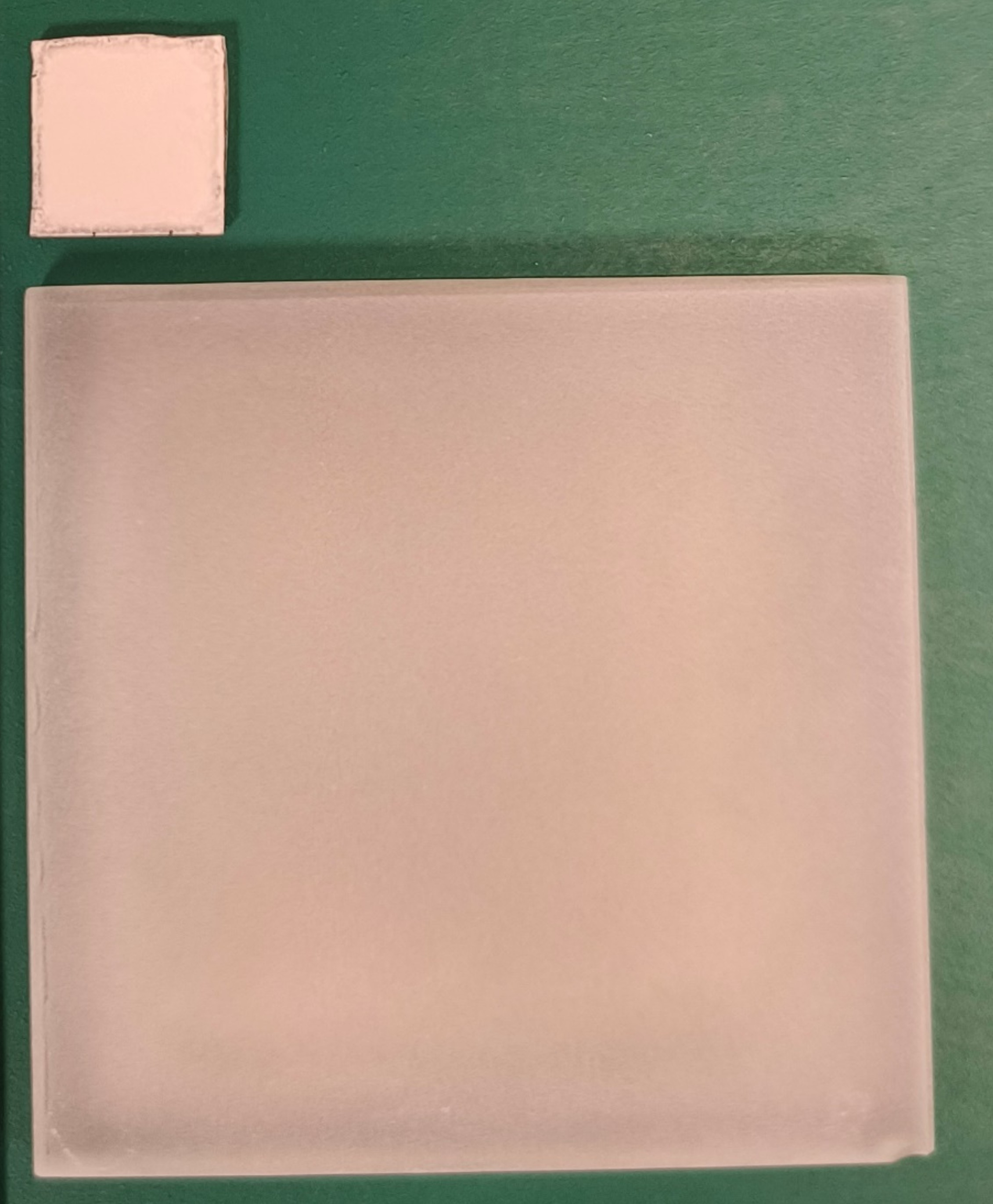

Supplement: Supplementary file 4 — Source data for Figs. 1–5. [file 44286_2026_406_MOESM4_ESM.zip › Source data main figures/Figure 1/1c/Photo 1.jpg]

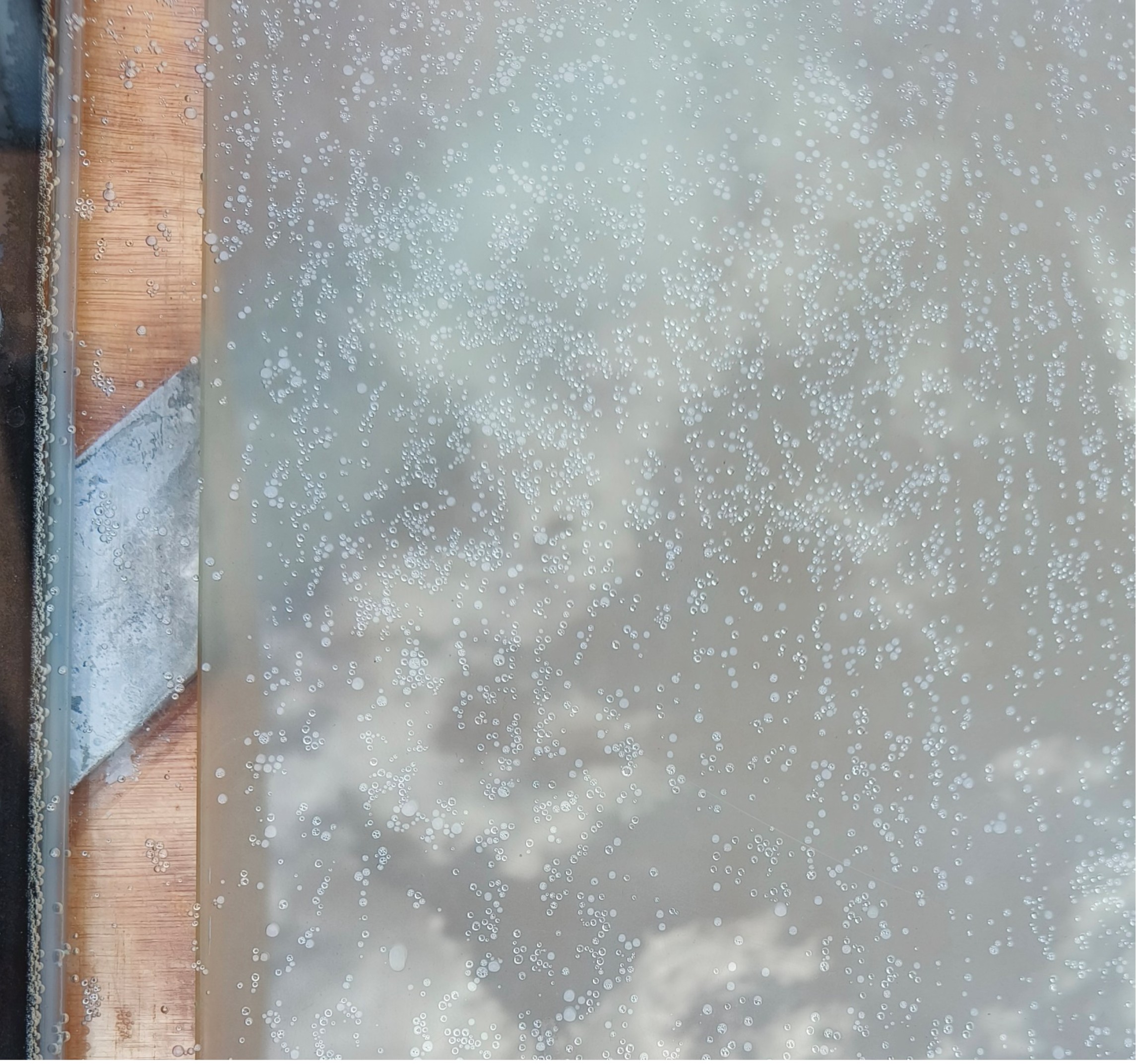

Supplement: Supplementary file 4 — Source data for Figs. 1–5. [file 44286_2026_406_MOESM4_ESM.zip › Source data main figures/Figure 5/3a/Photo 2.jpg]

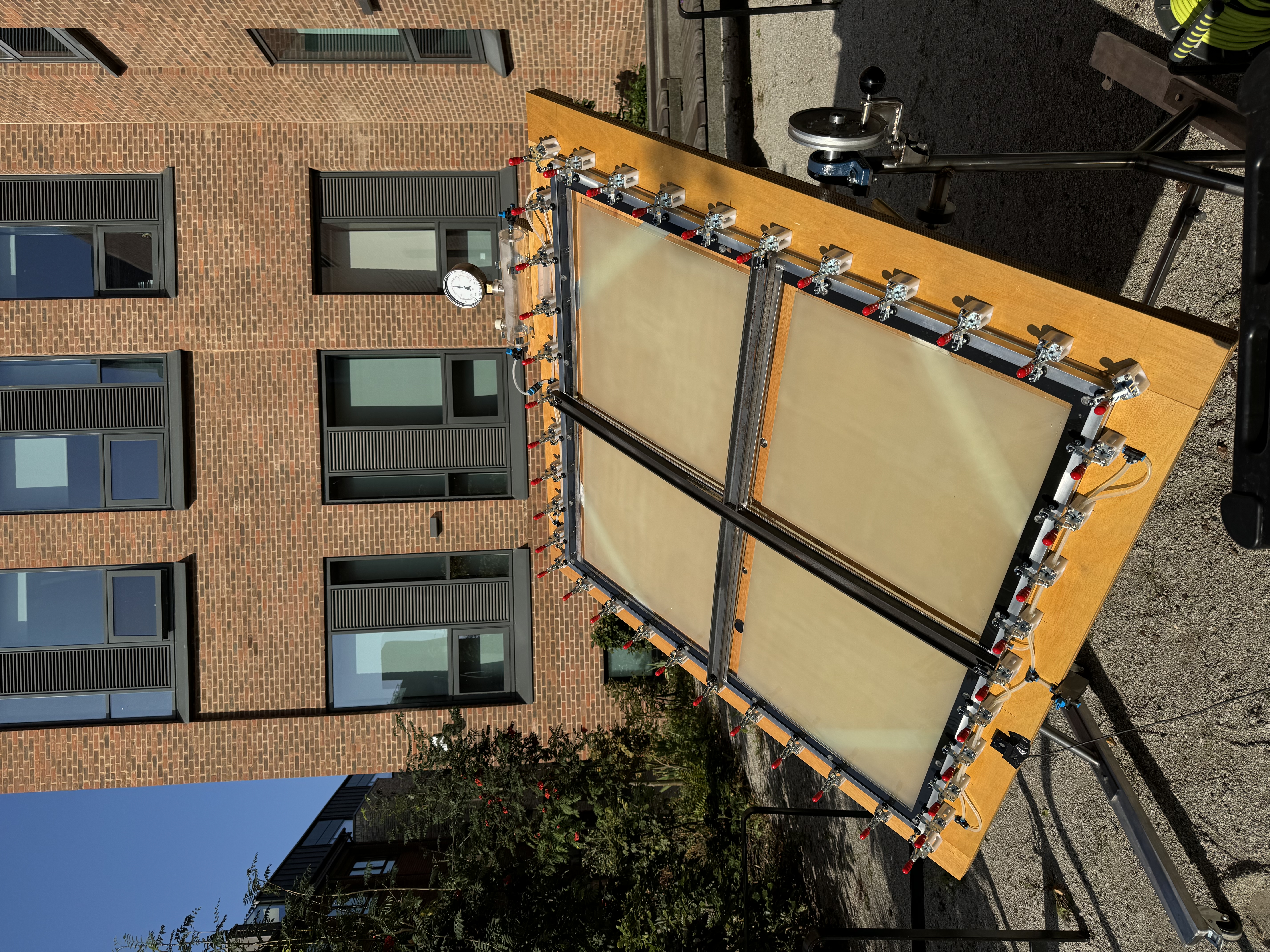

Supplement: Supplementary file 4 — Source data for Figs. 1–5. [file 44286_2026_406_MOESM4_ESM.zip › Source data main figures/Figure 5/3a/Photo 1.jpg]
